# Supplementary material for: P-TEFb Activation by RBM7 Shapes a Pro-survival Transcriptional Response to Genotoxic Stress
Source: Mol Cell. 2019 Apr 18;74(2):254–267.e10. doi: 10.1016/j.molcel.2019.01.033 (PMC6482433; doi:10.1016/j.molcel.2019.01.033)
Supplement: Document S1. Figures S1–S6 and Table S4 [file mmc1.pdf]

Molecular Cell, Volume *74*

Supplemental Information

P-TEFb Activation by RBM7 Shapes a Pro-survival Transcriptional Response to Genotoxic Stress

Andrii Bugai, Alexandre J.C. Quaresma, Caroline C. Friedel, Tina Lenasi, Robert Düster, Christopher R. Sibley, Koh Fujinaga, Petra Kukanja, Thomas Hennig, Melanie Blasius, Matthias Geyer, Jernej Ule, Lars Dölken, and Matjaž Barborič

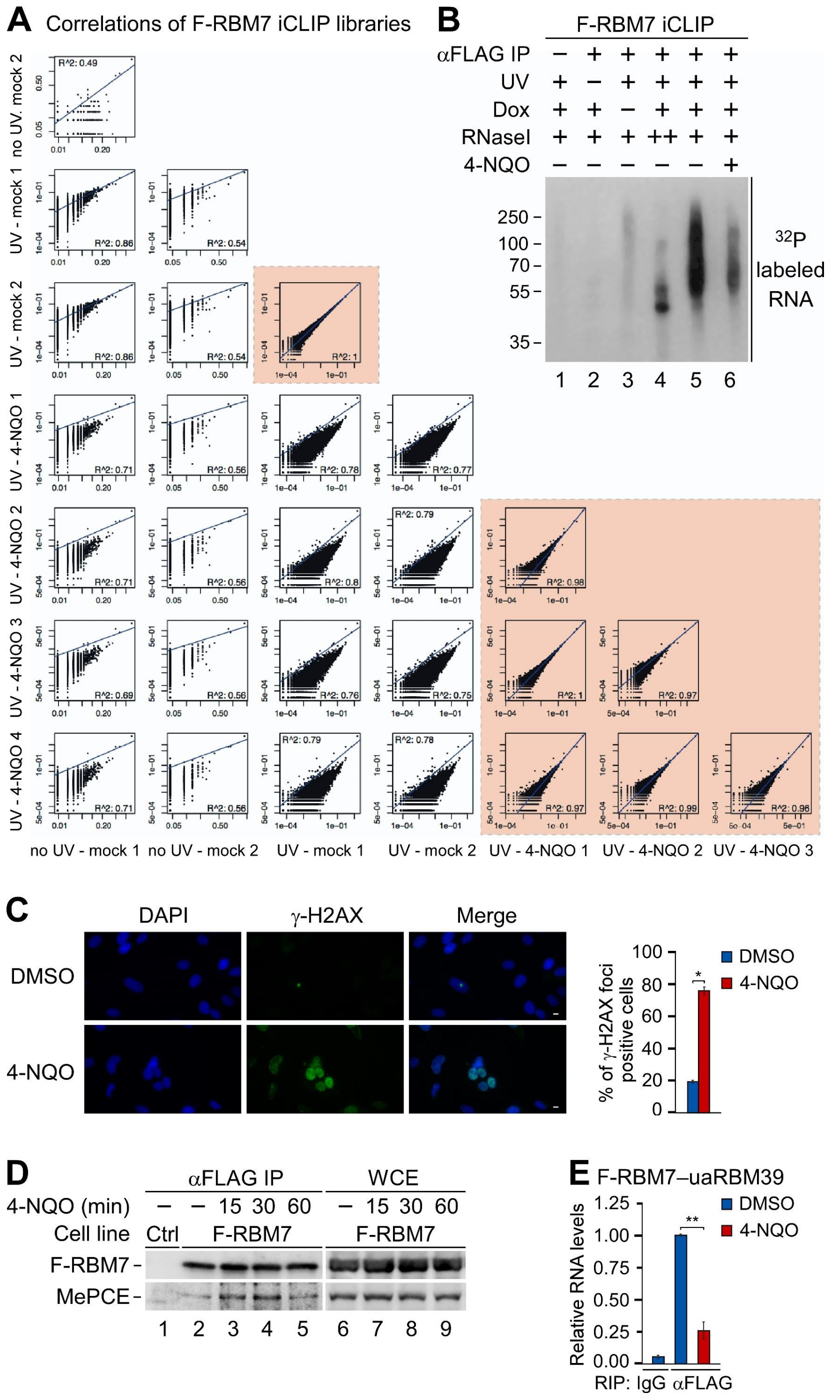

Figure S1│ F-RBM7 iCLIP. Related to Figure 1.

(A) Correlations between raw sequencing data of the indicated replicates of F-RBM7 iCLIP libraries.
(B) Autoradiogram of 32P-labelled RNA crosslinked to F-RBM7 in iCLIP. Prominent RNA–protein complexes are seen in the FLAG-M2 immuno-purifications (aFLAG IP) from HEK 293 cells expressing F-RBM7 upon induction by doxycycline (Dox) but not from control cells. Numbers on the left indicate molecular weight in kilodaltons.
(C) (Left) Representative DAPI and anti-g-H2AX staining images of HeLa cells are shown. Cells were treated with DMSO and 4-NQO as indicated. (Right) Quantification of g-H2AX foci-positive HeLa cells. The graph shows the percentage of cells with at least one nuclear g-H2AX foci. Values are plotted as the mean ± s.e.m. (n = 2). *, P < 0.01. Size bar = 10 mm.
(D) CoIP of F-RBM7 with MePCE from WCE of HEK 293 cells. Conditions with (in minutes) and without (-) 4-NQO are shown.
(E) RIP-qPCR uaRBM39 in F-RBM7 IP from HEK 293 WCE. Conditions with (red bars) and without (blue bars) 4-NQO (0.5 hr) are shown. Results are presented as the mean ± s.e.m. (n = 3). **, P < 0.01.

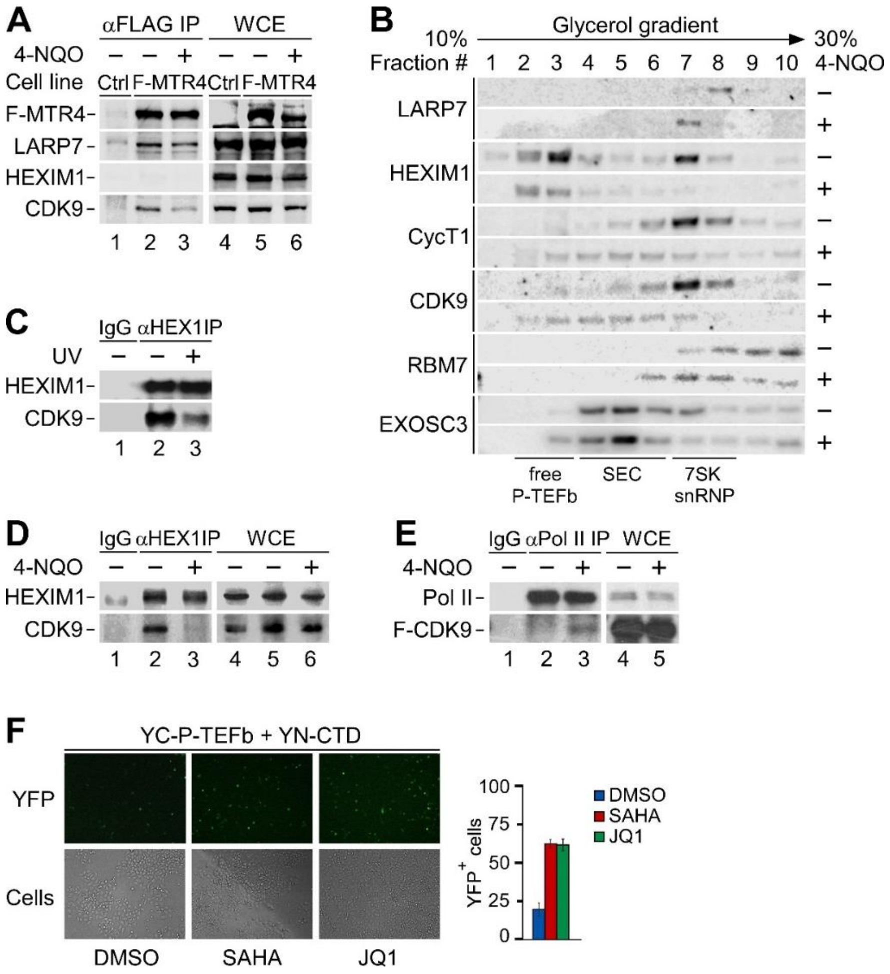

Figure S2│Genotoxic stress induces the relocation of P-TEFb from 7SK snRNP to Pol II. Related to Figure 2.

(A) CoIP of F-MTR4 with 7SK snRNP from WCE of HEK 293 cells. Conditions with (+) and without (-) 4-NQO are shown.
(B) Glycerol gradient (10 – 30 %) sedimentation analysis of WCE from HeLa cells. Conditions with (+) and without (-) 4-NQO are shown. The indicated proteins in collected fractions were detected by Western blotting. Fractions that contain P-TEFb in 7SK snRNP, super elongation complexes (SEC), and outside of these complexes are highlighted.
(C,D) CoIP of HEXIM1 with CDK9 from WCE of HeLa (C) and HFF-1 (D) cells. Conditions with (+) and without (-) UV are shown.
(E) CoIP of Pol II with F-CDK9 from WCE of HEK 293 cells. Conditions with (+) and without (-) 4-NQO are shown.
(F) V-PAC assay in HeLa cells expressing YC-P-TEFb and YN-CTD chimera. (Left) Representative YFP fluorescence (YFP) and phase contrast (Cells) images of cells are shown. Cell treatments are shown below the images. (Right) Quantification of YFP-positive cells that were treated as indicated. Values are plotted as the mean ± s.e.m. (n = 2).

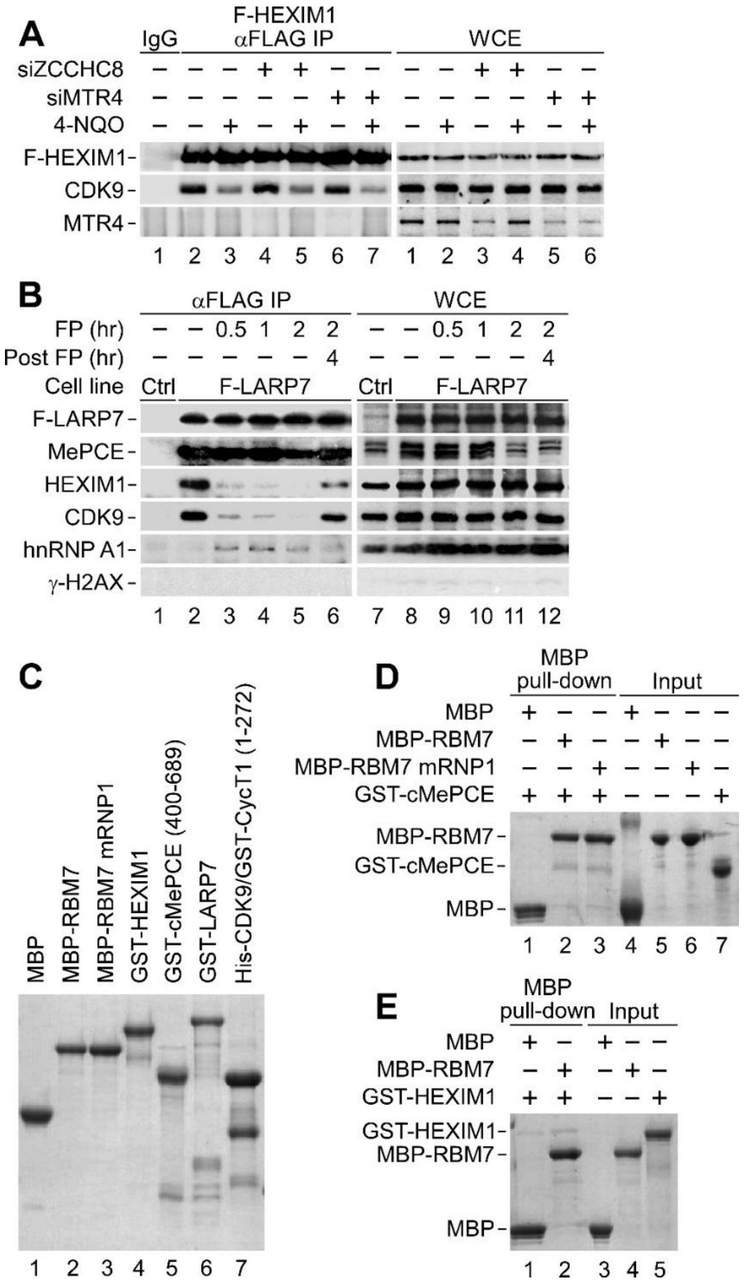

Figure S3│ RBM7 releases P-TEFb from the core of 7SK snRNP upon genotoxic stress. Related to Figure 4.

(A) CoIP of F-HEXIM1 with CDK9 from WCE of HEK 293 cells. Conditions with control (-), ZCCHC8 and MTR4 siRNA (+) are shown. The cells were treated (+) or not (-) with 4-NQO.
(B) Co-IP of F-LARP7 with 7SK snRNP and g-H2AX from WCE of HEK 293 cells. Conditions with (in hours) and without (-) FP are shown.
(C) Coomassie-stained gel of recombinant proteins used in *in vitro* MBP pull-down assays.
(D,E) Coomassie-stained gels of *in vitro* MBP pull-down assays of MBP-RBM7 proteins with GST-cMePCE (D) and GST-HEXIM1 (E).

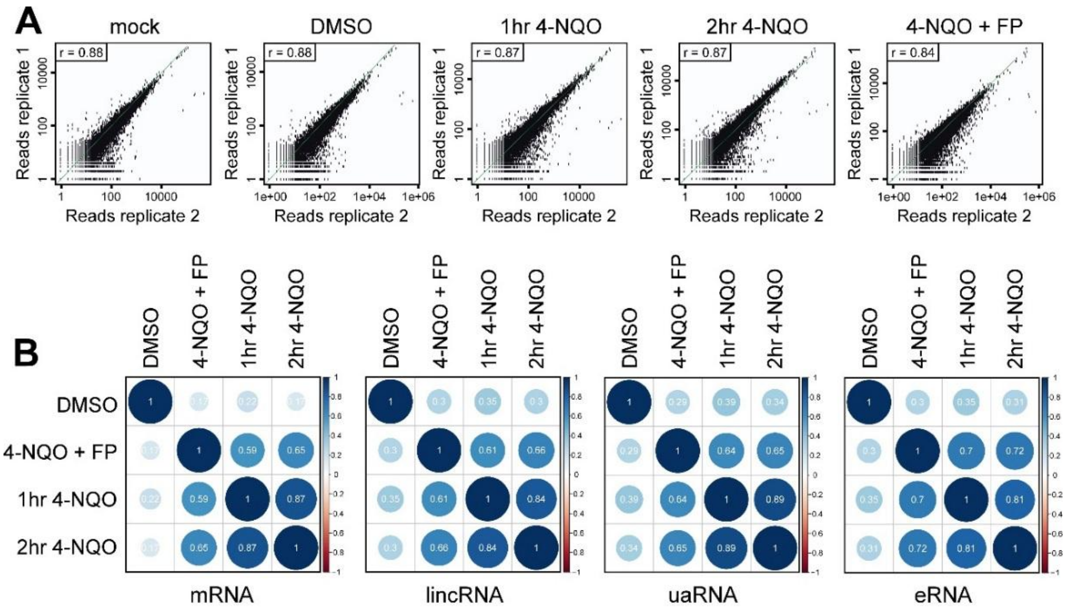

C

Enriched TF Binding Motif	High Confidence TF	Enrichment Score	Enriched Genes
dbcorrdb__NFYB__ENCSR000EGQ_1__m1 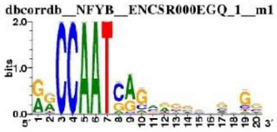	NF-YB	4,02	109
cisbp__M6174 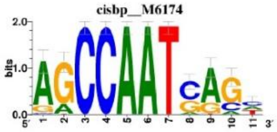	C/EBPζ	3,97	75
cisbp__M3089 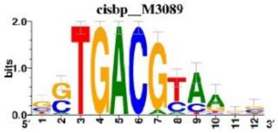	CREB1	3,97	90
swissregulon__hs__ATF4.p2 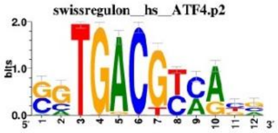	ATF4	3,95	120
dbcorrdb__FOS__ENCSR000EYZ_1__m1 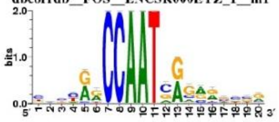	FOS	3,63	125
cisbp__M0311 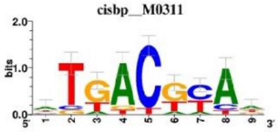	JUN	3,60	56

Figure S4│ Active P-TEFb is vital for Pol II transcriptional response to genotoxic stress. Related to Figure 5.

(A) Scatterplots comparing raw read counts for protein-coding and lincRNA genes from 4sU-seq data sets (n = 2) are shown and spearman correlation (*r*) is indicated. HeLa cells were treated as indicated on top of each graph.
(B) Spearman correlation between log2 fold-change values from 4sU-seq data sets (n = 2) are shown. HeLa cells were treated as indicated. Type of RNA transcript is shown below the graphs.
(C) Top transcription factor binding motifs within 5kb window around TSS of the 4FP gene set as revealed by RcisTarget. For each enriched motif, the annotated high confidence TF, normalized enrichment score, and number of genes highly ranked to the motif are shown.

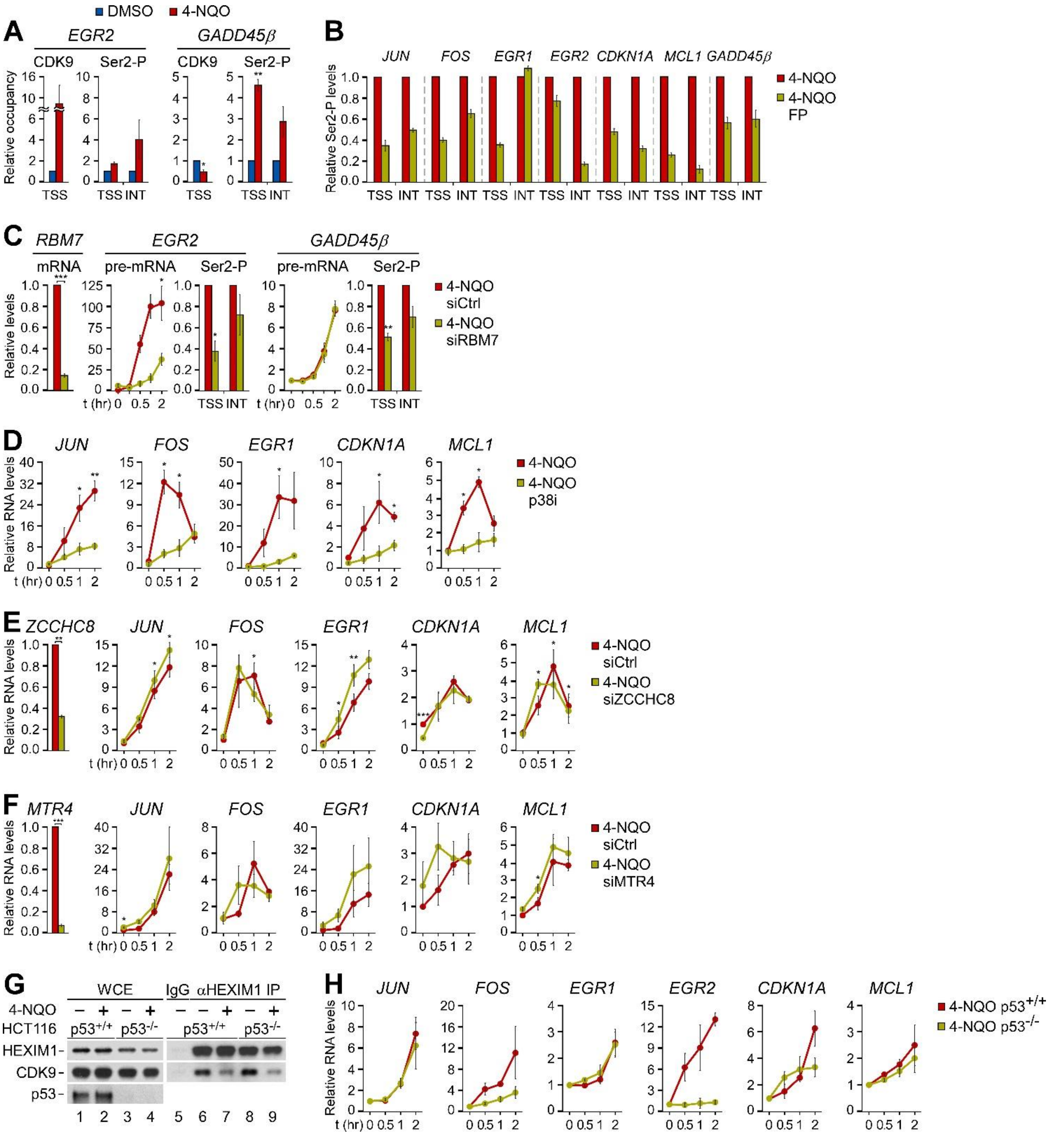

Figure S5│ RBM7 and 7SK snRNP are critical for the induction of P-TEFb-dependent DDR genes. Related to Figure 6.

(A) ChIP-qPCR of the occupancy of CDK9 and Ser2-P relative to Pol II at transcription start site (TSS) and in the middle of gene interior (INT) of the indicated DDR genes. Conditions with (red bars) and without (blue bars) 4-NQO are shown. Results were normalized to the DMSO control and are presented as the mean ± s.e.m. (n = 3). *, $P < 0.05$; **, $P < 0.01$, determined by Student's *t* test.
(B) ChIP-qPCR of the levels of Ser2-P relative to Pol II at transcription start site (TSS) and in the middle of gene interior (INT) of the indicated DDR genes. Conditions with 4-NQO (red bars) and with 4-NQO together with FP (yellow bars) are shown. Results were normalized to the 4-NQO values that were set to 1 and are presented as the mean ± s.e.m. Data shown are from one representative experiment (n = 3 technical replicates).
(C) RT-qPCR (left) of unspliced transcripts (pre-mRNA) of the indicated DDR genes and ChIP-qPCR (right) of the levels of Ser2-P relative to Pol II at transcription start site (TSS) and in the middle of gene interior (INT) of the indicated DDR genes

in control (siCtrl; red) and *RBM7* knockdown (siRBM7 #2; yellow) 4-NQO-treated HeLa cells. In RT-qPCR assays, the cells were exposed to 4-NQO for 15 min, 0.5 hr, 1 hr and 2 hr as indicated, and results were normalized to the untreated control and are presented as the mean ± s.e.m. (n = 3). *, P < 0.05; **, P < 0.01; ***, P < 0.001, determined by Student's *t* test. ChIP-qPCR results were normalized to the control values that were set to 1 and are presented as the mean ± s.e.m. (n = 3). *, P < 0.05; **, P < 0.01, determined by Student's *t* test. Efficacy of the *RBM7* knockdown is shown on the left. *** P < 0.001, determined by Student's *t* test.

(D) RT-qPCR of unspliced transcripts of the indicated DDR genes in 4-NQO-treated HeLa cells which were pre-treated or not with the p38 inhibitor (p38i) SB203580 for 1 hr. The cells were exposed to 4-NQO for 0.5 hr, 1 hr and 2 hr as indicated, and results were normalized to the untreated control and are presented as the mean ± s.e.m. (n = 3). *, P < 0.05; **, P < 0.01, determined by Student's *t* test.

(E,F) RT-qPCR of unspliced transcripts of the indicated DDR genes in control (siCtrl; red) and *ZCCHC8* (E; siZCCHC8, yellow) or *MTR4* (F; siMTR4, yellow) knockdown 4-NQO-treated HeLa cells. The cells were exposed to 4-NQO for 0.5 hr, 1 hr and 2 hr as indicated, and results were normalized to the untreated control and are presented as the mean ± s.e.m. (n = 3). *, P < 0.05; **, P < 0.01; ***, P < 0.001, determined by Student's *t* test. Efficacy of the *ZCCHC8* and *MTR4* knockdowns are shown on the left. **, P < 0.01; ***, P < 0.001, determined by Student's *t* test.

(G) CoIP of HEXIM1 with CDK9 from WCE of HCT116 $p53^{+/+}$ and HCT116 $p53^{-/-}$ cell lines. Conditions with (+) and without (-) UV are shown.

(H) RT-qPCR of unspliced transcripts of the indicated DDR genes in 4-NQO-treated HCT116 $p53^{+/+}$ (red) and HCT116 $p53^{-/-}$ (yellow) cell lines. The cells were exposed to 4-NQO for 0.5 hr, 1 hr and 2 hr as indicated, and results were normalized to the respective untreated control and are presented as the mean ± s.e.m. (n = 3).

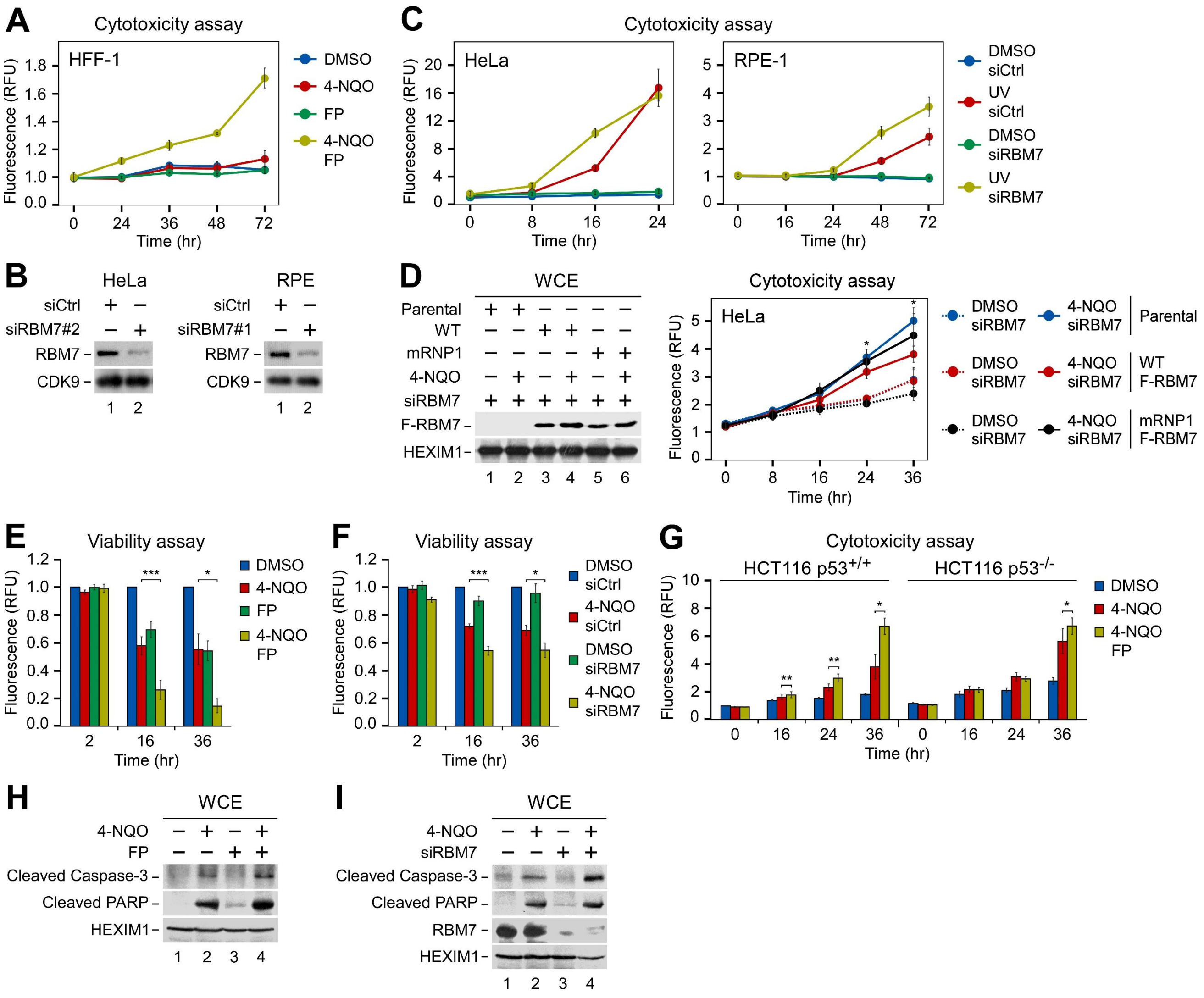

Figure S6│ P-TEFb and RBM7 promote cell viability upon genotoxic stress. Related to Figure 7.

(A,C) Hypersensitivity of HFF-1, HeLa and RPE-1 cells to genotoxic stress upon FP treatment (A) and RBM7 depletion (C). The cells were treated as indicated by the legends and examined at the time points indicated below the graphs. Two independent siRNAs (siRBM7 #2, HeLa cells; siRBM7 #1, RPE-1 cells) were used to deplete RBM7. Cytotoxicity results are presented as fluorescence values relative to the untreated control and plotted as the mean ± s.e.m. (n = 2).
(B) Western blotting analysis of RBM7 depletion in HeLa and RPE-1 cells using RNAi.
(D) Expression of F-RBM7 increases survival of 4-NQO-treated HeLa cells with depleted levels of endogenous RBM7. The parental, WT and mRNP1 F-RBM7-expressing HeLa cells were treated as indicated by the legends and examined at the time points indicated below the graphs. siRBM7 #2 was used to deplete RBM7. Cytotoxicity results are presented as fluorescence values relative to the untreated control and plotted as the mean ± s.e.m. (n = 3). *, $P < 0.05$, determined by Student's *t* test using 4-NQO siRBM7 data sets from parental and F-RBM7 WT HeLa cells. Levels of the F-RBM7 proteins in *RBM7* knockdown cells are shown on the left.
(E,F) FP treatment (E) and RBM7 depletion (F) decrease viability of 4-NQO-treated HeLa cells. The cells were treated as indicated by the legends and examined at the time points indicated below the graphs. siRBM7 #2 was used to deplete RBM7. Viability results are presented as fluorescence values relative to the untreated control and plotted as the mean ± s.e.m. (n = 3). *, $P < 0.05$; ***, $P < 0.001$, determined by Student's *t* test.
(G) Hypersensitivity of HCT116 $p53^{+/+}$ cells to genotoxic stress upon FP treatment. HCT116 cell lines were treated as indicated by the legend and examined at the time points indicated below the graphs. Cytotoxicity results are presented as fluorescence values relative to the untreated control in HCT116 $p53^{+/+}$ cells and plotted as the mean ± s.e.m. (n = 3) *, $P < 0.05$; **, $P < 0.01$, determined by Student's *t* test.
(H,I) Western blotting analysis of Caspase-3 and PARP cleavage using the cleaved products-specific antibodies. HeLa cells were treated with DMSO, 4-NQO, and FP for eight hours as indicated. siRBM7 #2 was used to deplete RBM7.

Table S4. Specificity of CDK9, Pol II and Ser2-P ChIP-qPCR assays. Related to Figure 6.

Enrichments of CDK9 ChIP values over IgG

Ab - Treatment	*JUN* TSS	*FOS* TSS	*EGR1* TSS	*EGR2* TSS	*CDKN1A* TSS	*MCL1* TSS	*GADD45β* TSS
CDK9 - DMSO	10,4	10,8	3,8	1,5	24,7	38,4	21,9
CDK9 - 4-NQO	31,2	16,0	267,3	6,6	3,1	60,9	18,0

Enrichments of CDK9 ChIP values over *FOS* intergenic region

Ab - Treatment	*JUN* TSS	*FOS* TSS	*EGR1* TSS	*EGR2* TSS	*CDKN1A* TSS	*MCL1* TSS	*GADD45β* TSS
CDK9 - DMSO	2,6	7,0	1,3	0,5	1,4	4,4	11,2
CDK9 - 4-NQO	7,5	17,1	12,3	2,2	2,0	8,8	5,5

Enrichments of Pol II and Ser2-P ChIP values over IgG

	JUN		*FOS*		*EGR1*		*EGR2*		*CDKN1A*		*MCL1*		*GADD45β*	
Ab - Treatment	TSS	INT	TSS	INT	TSS	INT	TSS	INT	TSS	INT	TSS	INT	TSS	INT
Pol II - DMSO	13,1	4,0	17,7	1,7	5,4	2,5	3,2	1,1	6,4	3,1	28,1	14,4	37,7	19,2
Pol II - 4-NQO	60,8	124,1	38,6	15,0	150,4	95,3	6,9	7,8	19,6	40,0	63,1	18,1	108,0	81,3
Ser2-P - DMSO	7,8	7,7	11,8	10,9	5,7	3,5	2,2	1,3	3,0	22,2	26,4	112,7	24,5	41,6
Ser2-P - 4-NQO	210,5	661,7	93,0	99,5	719,2	555,0	12,9	37,0	130,9	286,8	222,8	81,9	234,9	429,1

Enrichments of Pol II and Ser2-P ChIP values over *FOS* intergenic region

	JUN		*FOS*		*EGR1*		*EGR2*		*CDKN1A*		*MCL1*		*GADD45β*	
Ab - Treatment	TSS	INT	TSS	INT	TSS	INT	TSS	INT	TSS	INT	TSS	INT	TSS	INT
Pol II - DMSO	20,8	4,1	31,3	1,7	3,4	1,8	0,9	1,0	8,1	4,1	31,6	3,2	119,0	17,5
Pol II - 4-NQO	24,0	19,8	49,4	9,0	19,4	14,3	1,0	3,9	1,5	5,7	29,7	10,6	43,3	24,8
Ser2-P - DMSO	12,3	7,8	20,9	10,8	3,6	2,6	0,6	1,3	3,8	29,2	29,7	25,4	77,3	37,9
Ser2-P - 4-NQO	83,3	105,5	119,1	59,5	92,8	83,4	1,9	18,7	9,9	40,7	104,9	47,9	94,1	131,1

Enrichments of the CDK9, Pol II and Ser2-P chromatin occupancies over the normal IgG and *FOS* intergenic site controls are shown. Conditions with (red lines) and without (blue lines) 4-NQO are shown. Data shown are derived from mean % of input values corresponding to the data presented in Figure 6A, and are presented as fold-enrichments over the controls which were set to 1. (n = 3).
