# Supplementary material for: P-TEFb Activation by RBM7 Shapes a Pro-survival Transcriptional Response to Genotoxic Stress
Source: Mol Cell. 2019 Apr 18;74(2):254–267.e10. doi: 10.1016/j.molcel.2019.01.033 (PMC6482433; doi:10.1016/j.molcel.2019.01.033)
Supplement: Document S2. Article plus Supplemental Information [file mmc6.pdf]

Article

Molecular Cell

# P-TEFb Activation by RBM7 Shapes a Pro-survival Transcriptional Response to Genotoxic Stress

## Graphical Abstract

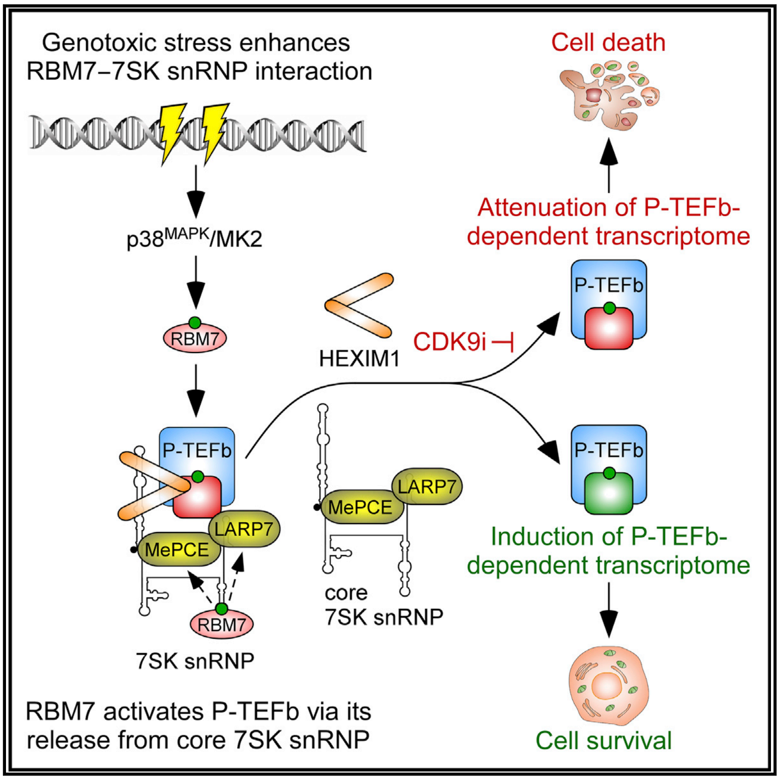


## Authors

Andrii Bugai,
Alexandre J.C. Quaresma,
Caroline C. Friedel, ..., Jernej Ule,
Lars Dölken, Matjaž Barborič

## Correspondence

matjaz.barboric@helsinki.fi

## In Brief

Bugai et al. report that stimulation of Pol II pause release is central to the DNA damage response. Mechanistic studies uncover the vital role of RBM7 in the genotoxic-stress-induced release of P-TEFb from the inhibitory 7SK snRNP, which enables a transcriptional response that is crucial to the survival of stressed cells.

## Highlights

- iCLIP identifies a genotoxic-stress-enhanced interaction of RBM7 with 7SK snRNA
- RBM7 interacts with core subunits of 7SK snRNP to activate P-TEFb
- Induced Pol II transcription through P-TEFb is a hallmark of the DNA damage response
- The RBM7-P-TEFb axis promotes viability of genotoxic-stressed cells

## Data Resources

GSE110272

Bugai et al., 2019, Molecular Cell *74*, 254–267
April 18, 2019 © 2019 The Authors. Published by Elsevier Inc.
https://doi.org/10.1016/j.molcel.2019.01.033

# P-TEFb Activation by RBM7 Shapes a Pro-survival Transcriptional Response to Genotoxic Stress

Andrii Bugai,[1] Alexandre J.C. Quaresma,[1] Caroline C. Friedel,[2,11] Tina Lenasi,[1,11] Robert Düster,[3,11] Christopher R. Sibley,[4,5] Koh Fujinaga,[6] Petra Kukanja,[1] Thomas Hennig,[7] Melanie Blasius,[8] Matthias Geyer,[3] Jernej Ule,[5,9,10] Lars Dölken,[7] and Matjaž Barborič[1,12,*]
[1]Department of Biochemistry and Developmental Biology, University of Helsinki, Helsinki 00014, Finland
[2]Institute for Informatics, Ludwig-Maximilians-Universität München, 80333 Munich, Germany
[3]Institute of Structural Biology, University of Bonn, 53127 Bonn, Germany
[4]Division of Brain Sciences, Department of Medicine, Imperial College London, London W12 0NN, UK
[5]MRC-Laboratory of Molecular Biology, Francis Crick Avenue, Cambridge CB2 0QH, UK
[6]Departments of Medicine, Microbiology, and Immunology, University of California, San Francisco, San Francisco, CA 94143, USA
[7]Institute for Virology and Immunobiology, University of Würzburg, 97078 Würzburg, Germany
[8]Department of Cellular and Molecular Medicine, Center for Healthy Aging, University of Copenhagen, 2200 Copenhagen, Denmark
[9]Institute of Neurology, University College London, London WC1N 3BG, UK
[10]The Francis Crick Institute, 1 Midland Road, London NW1 1AT, UK
[11]These authors contributed equally
[12]Lead Contact
*Correspondence: matjaz.barboric@helsinki.fi
https://doi.org/10.1016/j.molcel.2019.01.033

## SUMMARY

**DNA damage response (DDR) involves dramatic transcriptional alterations, the mechanisms of which remain ill defined. Here, we show that following genotoxic stress, the RNA-binding motif protein 7 (RBM7) stimulates RNA polymerase II (Pol II) transcription and promotes cell viability by activating the positive transcription elongation factor b (P-TEFb) via its release from the inhibitory 7SK small nuclear ribonucleoprotein (7SK snRNP). This is mediated by activation of p38$^{MAPK}$, which triggers enhanced binding of RBM7 with core subunits of 7SK snRNP. In turn, P-TEFb relocates to chromatin to induce transcription of short units, including key DDR genes and multiple classes of non-coding RNAs. Critically, interfering with the axis of RBM7 and P-TEFb provokes cellular hypersensitivity to DNA-damage-inducing agents due to activation of apoptosis. Our work uncovers the importance of stress-dependent stimulation of Pol II pause release, which enables a pro-survival transcriptional response that is crucial for cell fate upon genotoxic insult.**

## INTRODUCTION

The cellular DNA damage response (DDR) has evolved to detect and repair lesions that are generated continuously by external and internal DNA-damaging agents (Hoeijmakers, 2009). In parallel, activation of the DDR halts progression of the cell cycle to provide time for repair, the outcome of which determines whether cells will re-enter the cell cycle and continue with their physiological program, enter into senescence, or die by apoptosis. A defective DDR can lead to genomic instability underlying many diseases, including hematological disorders and cancer (Jackson and Bartek, 2009). Importantly, it is widely accepted that cells need to shut down RNA polymerase II (Pol II) transcription in response to UV-induced bulky DNA lesions and other types of DNA damage, which can facilitate repair and limit the production of abnormal transcripts (Giono et al., 2016). While transcription can be inhibited transiently at the initiation and elongation stages (Awwad et al., 2017; Rockx et al., 2000; Williamson et al., 2017) or irreversibly through degradation of stalled Pol II (Wilson et al., 2013), it is eventually restored once the damage is corrected. However, the significance of mounting transcriptional activation following genotoxic stress remains poorly understood.

Transition of Pol II from promoter-proximal pausing to productive elongation represents a critical regulatory step in metazoan gene expression (Zhou et al., 2012). At most active genes, Pol II transcribes 20–100 nt from the transcription start site (TSS) before its elongation is paused by the multisubunit negative transcription elongation factors (N-TEFs), consisting of negative elongation factor (NELF) and DRB-sensitivity inducing factor (DSIF). The release of paused Pol II genome-wide is stimulated by positive transcription elongation factor b (P-TEFb), which is composed of the catalytic CDK9 kinase and a regulatory CycT1 or CycT2 subunit (Gressel et al., 2017; Zhou et al., 2012). Upon its recruitment or activation at target gene promoters or enhancers, P-TEFb phosphorylates serine 2 residues (Ser2-P) within the C-terminal domain (CTD) $Y^1S^2P^3T^4S^5P^6S^7$ heptapeptide repeats of the largest Pol II subunit, RPB1, as well as NELF-E and the SPT5 subunit of DSIF, leading to productive Pol II elongation (Bacon and D'Orso, 2018; Zhou et al., 2012). In addition, clearance of Pol II from the pause sites enables new transcriptional initiation, augmenting the production of RNA (Gressel et al., 2017).

 © 2019 The Authors. Published by Elsevier Inc.
This is an open access article under the CC BY license (http://creativecommons.org/licenses/by/4.0/).

Considering that P-TEFb is crucial for prompt expression of stimulus-induced genes (Liu et al., 2015), we reasoned that it might feature prominently in activating Pol II transcription following DNA damage. Importantly, a major fraction of P-TEFb resides within 7SK small nuclear ribonucleoprotein (7SK snRNP), in which coordinated actions of the scaffolding 7SK small nuclear RNA (7SK) and three RNA-binding proteins (RBPs) inhibit the kinase. While the 7SK $\gamma$-methylphosphate capping enzyme MePCE and LARP7 stabilize 7SK to form the core of 7SK snRNP, HEXIM1 subsequently interacts with 7SK of the core to bind and inhibit P-TEFb (Michels and Bensaude, 2018; Quaresma et al., 2016). Of note, activation of P-TEFb via its release from 7SK snRNP, as exemplified by the RNA binding HIV-1 transcriptional transactivator Tat (Ott et al., 2011), has been identified as a rate-limiting event that stimulates Pol II pause release at specific genes (Quaresma et al., 2016). Given that cellular RBPs are emerging as important effectors in the DDR (Dutertre et al., 2014) and transcription (Quaresma et al., 2016; Skalska et al., 2017), we envisioned that a protein from this class could facilitate genotoxic-stress-induced Pol II transcription through 7SK snRNP.

In this study, we focused our efforts on the ubiquitously expressed RNA-binding motif protein 7 (RBM7), which promotes survival of cells following DNA damage generated by UV or its mimicking genotoxic and carcinogenic chemical 4-nitroquinoline 1-oxide (4-NQO) (Blasius et al., 2014). RBM7 binds the DExH/D box RNA helicase MTR4 via the bridging zinc-knuckle protein ZCCHC8, forming the nuclear exosome targeting complex (NEXT) (Falk et al., 2016; Lubas et al., 2011). Through the RNA-binding capacity of RBM7, NEXT promotes degradation of multiple RNA classes, including upstream antisense RNA (uaRNA) and enhancer-derived RNA (eRNA) transcripts, as well as 3′ end extended forms of snRNA, small nucleolar RNA (snoRNA), and histone gene transcripts (Andersen et al., 2013; Hallais et al., 2013; Lubas et al., 2011). Furthermore, NEXT targets pre-mRNAs for decay and/or processing of intron-embedded snoRNAs and microRNAs (miRNAs) (Lubas et al., 2015). Finally, NEXT associates with the cap-binding protein complex and functions in connecting transcription termination with exosomal degradation (Andersen et al., 2013; Hallais et al., 2013). Although a linkage between RBM7 and DDR has been reported (Blasius et al., 2014), it remains unclear how RBM7 exerts its pro-survival function. Here, we uncovered an unexpected interplay between RBM7 and 7SK snRNP in the wake of DNA damage, shining a spotlight on the central role of P-TEFb kinase in shaping a transcriptional response that is crucial for viability of genotoxic-stressed cells.

## RESULTS

### iCLIP Reveals a Genotoxic-Stress-Enhanced Interaction of RBM7 with 7SK

We postulated that defining the RNA interactome of RBM7 under unchallenged and DNA-damage conditions should disclose insights into its critical function in genotoxic-stressed cells. Thus, we performed an individual-nucleotide-resolution UV crosslinking and immunoprecipitation (iCLIP) assay in human HEK293 Flp-In T-Rex (HEK293) cells that expressed 3XFLAG-epitope-tagged RBM7 (F-RBM7). Because UV irradiation yields RNA-protein crosslinks immediately and thus prior to DDR activation, we instead employed its mimetic, 4-NQO. Notably, 4-NQO metabolite 4-hydroxyaminoquinolone 1-oxide forms bulky DNA adducts on purines, which are removed by nucleotide excision repair (NER) (Ikenaga et al., 1977). Consistent with the previous report (Lubas et al., 2015), RBM7 bound directly a diverse set of RNAs, including genic, intergenic, and non-coding RNAs (ncRNAs) (Figures 1A, S1A, and S1B; Table S1A). We ranked these RNAs by the change in binding following 4-NQO exposure, which showed increased RBM7 binding to snRNAs, including 7SK, spliceosomal snRNAs, and other ncRNAs, and decreased RBM7 binding to specific pre-mRNAs (Tables S1B and S1C). As expected, 4-NQO exposure increased $\gamma$-H2AX foci formation, confirming activation of the DDR (Figure S1C). Given the pivotal role of 7SK snRNP in regulating Pol II pause release (Quaresma et al., 2016), we investigated the potential connection between RBM7 and 7SK in controlling Pol II transcription following genotoxic stress.

We next plotted the RBM7 iCLIP reads on a secondary structure model of 7SK. This revealed that RBM7 binds selectively stem-loop 3 (SL3) of 7SK (Figure 1B), thereby placing it between MePCE/HEXIM1 and LARP7 that bind SL1 and SL4, respectively (Quaresma et al., 2016). A quantitative RNA immunoprecipitation (RIP-qPCR) assays proved the iCLIP result to be specific, since substituting the conserved Lys60, Phe62, and Phe64 residues of the ribonucleoprotein 1 (RNP1) motif within the RNA recognition motif (RRM) of RBM7 to alanines (mRNP1) abrogated the interaction (Figure 1C). Furthermore, while the interaction of RBM7 with 7SK increased at 30 min of 4-NQO treatment, it decreased at 2 h to ~50% of the interaction in unchallenged cells (Figure 1D). Paralleling this dynamic, the interaction of F-RBM7 with the core 7SK snRNP subunit MePCE peaked at 30 min of 4-NQO exposure as revealed by a co-immunoprecipitation (coIP) experiment (Figure S1D). Of note, 4-NQO treatment led to decreased binding of RBM7 with the uaRNA of *RBM39*, which is consistent with the DNA-damage-induced release of uaRNAs from NEXT (Blasius et al., 2014; Tiedje et al., 2015) (Figure S1E). Together, these results identify RBM7 as a 7SK-interacting protein, suggesting its role in the cellular response to genotoxic stress via 7SK snRNP.

### Genotoxic Stress Stimulates the Relocation of P-TEFb from 7SK snRNP to Pol II

To test if RBM7 binds the rest of 7SK snRNP subunits, we performed a coIP analysis in HEK293 cells. This showed the interaction of F-RBM7 with endogenous LARP7 and CDK9, but not HEXIM1 (Figure 2A). We obtained the same result when we used 3XFLAG-epitope-tagged MTR4 (F-MTR4) as the bait (Figure S2A), suggesting that all NEXT subunits bind 7SK snRNP in unchallenged cells. Importantly, 2 h of 4-NQO treatment resulted in the release of endogenous CDK9, HEXIM1, and RBM7 from 7SK snRNP in HEK293 and HeLa Flp-In (HeLa) cells, as determined by coIP analysis with 3XFLAG-epitope-tagged LARP7 (F-LARP7) and glycerol gradient centrifugation analysis, respectively (Figures 2B and S2B). Likewise, exposing HeLa cells to UV and primary human foreskin fibroblasts (HFF-1) to 4-NQO released CDK9 from HEXIM1 (Figures S2C and S2D).

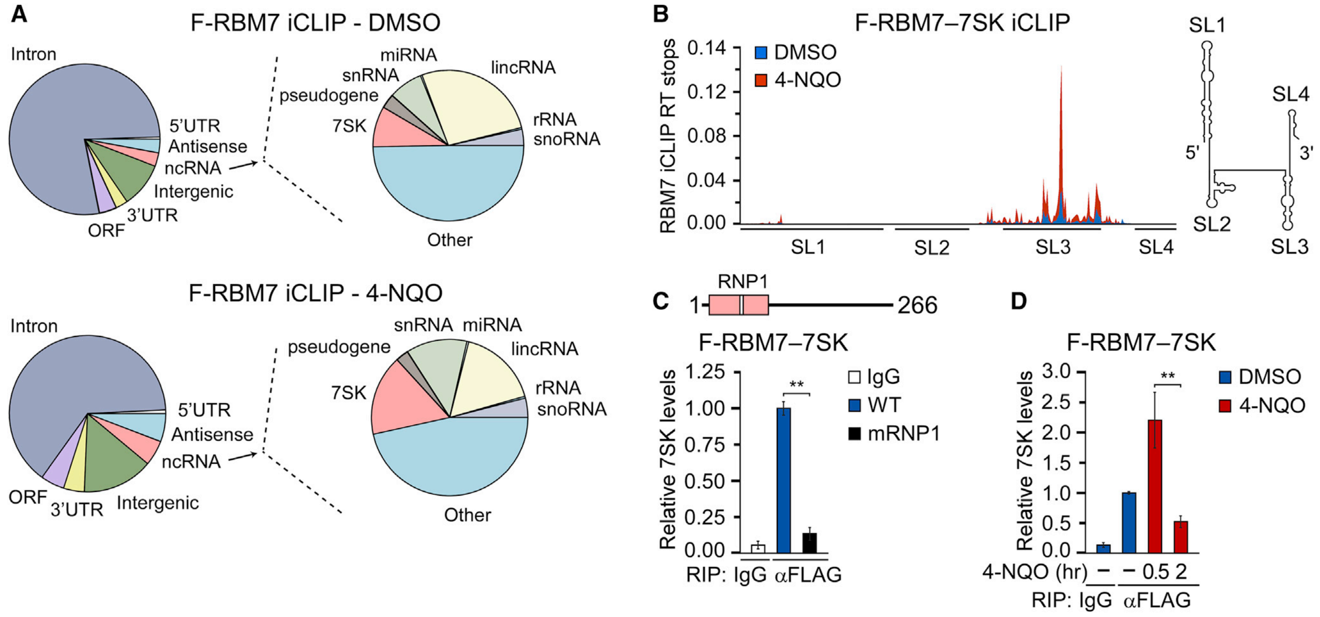


**Figure 1. Genotoxic Stress Induces the Interaction of RBM7 with 7SK**

(A) Distribution charts of unique tags derived from the F-RBM7 libraries based on percentages of the total iCLIP reads and mapped to the indicated RNA classes. Charts on the right show distribution of the indicated types of ncRNA.

(B) F-RBM7 iCLIP reads mapped to 7SK. Positions of the four stem-loops (SL1–4) are shown below the iCLIP reads and on a 7SK secondary structure model.

(C) RIP-qPCR of 7SK in wild-type and mRNP1 F-RBM7 IP from whole-cell extracts (WCEs) of HEK293 cells. RBM7 with RRM (in pink) and the position of RNP1 (white stripe) is shown on top.

(D) RIP-qPCR of 7SK in F-RBM7 IP from WCE of HEK293 cells. Conditions with (red bars; in hours) and without (blue bars) 4-NQO are shown.

Results in (C) and (D) are presented as the mean ± SEM (n = 3). $^{**}p < 0.01$. See also Figure S1 and Tables S1A–S1C.

To follow the relocation of P-TEFb upon genotoxic stress further, we examined its interaction with RBM7 and Pol II by conducting a series of coIP and bimolecular fluorescence complementation (BiFC) assays. 2 h of 4-NQO treatment increased the interaction of 3XFLAG-epitope-tagged CDK9 (F-CDK9) with RBM7 (Figure 2C), indicating that the initial enhanced binding between RBM7 and 7SK snRNP is followed by the release of RBM7 in a complex with P-TEFb. Notably, interaction of the released CDK9 with F-MTR4 decreased following 4-NQO exposure (Figure S2A), implying a remodeling of NEXT upon genotoxic stress. Importantly, the released F-CDK9 and F-RBM7 displayed enhanced interaction with the transcriptionally engaged Ser2-P form of Pol II upon DNA damage (Figures 2C and 2D). Likewise, 4-NQO treatment enhanced the binding between F-CDK9 and total Pol II (Figure S2E), which is likely mediated by direct interaction of the CycT1 histidine-rich domain with the CTD of Pol II (Lu et al., 2018). To extend these findings, we evaluated whether 4-NQO treatment triggered relocation of P-TEFb to the CTD in living HeLa cells by conducting a visualization of P-TEFb activation (V-PAC) assay (Fujinaga et al., 2015). Here, BiFC of transiently expressed YC-P-TEFb and YN-CTD chimera containing the C- and N-terminal regions of yellow fluorescent protein (YFP), respectively, indicates the relocation (Figure 2E). Corroborating our coIP results, 4-NQO exposure increased the number of YFP-positive cells to a similar extent as the known P-TEFb releasing agents SAHA and JQ1 (Bartholomeeusen et al., 2012; Contreras et al., 2009) (Figures 2F and S2F). Together, these findings establish that RBM7 binds 7SK snRNP and that genotoxic stress activates P-TEFb by relocating it from 7SK snRNP to the CTD of Pol II.

## Genotoxic Stress Triggers the Release of P-TEFb from HEXIM1 via RBM7 and p38$^{MAPK}$

The observations gathered thus far led us to hypothesize that following DNA damage, RBM7 activates P-TEFb by promoting its release from the 7SK snRNP. Indeed, the 4-NQO-induced release of CDK9 from 3XFLAG-epitope-tagged HEXIM1 (F-HEXIM) was abrogated in *RBM7* knockdown cells (Figure 3A). In a complementary approach, ectopic expression of F-RBM7 in HEK293 cells decreased the interaction of endogenous HEXIM1 with CDK9 and 7SK, but this effect was lost when using the 7SK-binding-deficient mRNP1 F-RBM7 (Figure 3B). It is likely that overexpression of F-RBM7 alleviated the requirement of genotoxic stress for P-TEFb activation in this system. Because UV irradiation triggers phosphorylation of RBM7 via the p38$^{MAPK}$-MK2 pathway (Blasius et al., 2014; Borisova et al., 2018), we examined the importance of this signaling cascade for P-TEFb activation. While 30 min of 4-NQO exposure activated p38$^{MAPK}$ and induced the release of CDK9 from HEXIM1, pharmacological inhibition of p38$^{MAPK}$ with SB203580 (p38i) interfered with the release (Figure 3C). Importantly, the blockade of p38$^{MAPK}$ diminished the 4-NQO-enhanced interaction of RBM7 with 7SK (Figure 3D). Together, these results show the critical role of RBM7 and p38$^{MAPK}$ in genotoxic-stress-induced activation of P-TEFb.

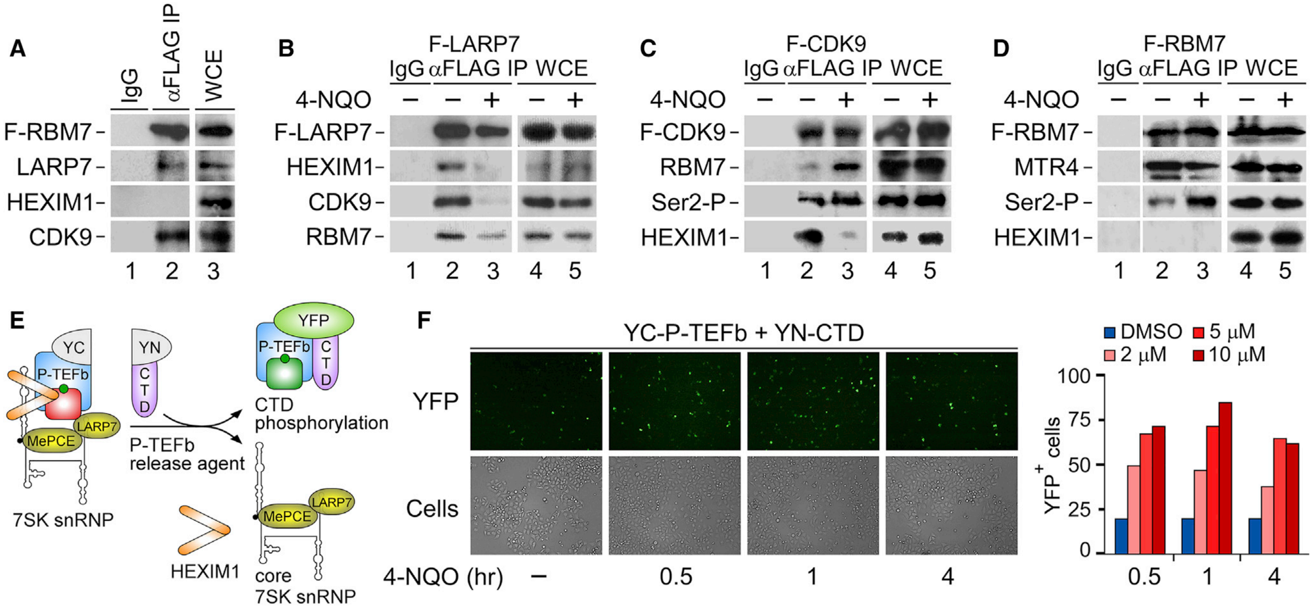


**Figure 2. Genotoxic Stress Induces the Relocation of P-TEFb and RBM7 from 7SK snRNP to Pol II**

(A and B) CoIP of F-RBM7 (A) and F-LARP7 (B) with 7SK snRNP from WCE of HEK293 cells. Conditions with (+) and without (–) 4-NQO are shown.

(C and D) CoIP of F-CDK9 (C) and F-RBM7 (D) with the indicated proteins from WCE of HEK293 cells. Conditions with (+) and without (–) 4-NQO are shown.

(E) Cartoon depicting the V-PAC assay. P-TEFb-releasing agents induce the transfer of the inactive YC-P-TEFb (inactive CDK9 in red) to the substrate YN-CTD chimera (active CDK9 in green), yielding YFP fluorescence.

(F) V-PAC assay in HeLa cells expressing YC-P-TEFb and YN-CTD chimera. Left: representative YFP fluorescence (YFP) and phase contrast (cells) images of cells are shown. Conditions with (in hours) and without (–) 4-NQO are shown. Right: quantification of YFP-positive cells that were treated as indicated.

See also Figure S2.

## RBM7 Releases P-TEFb from the Core of 7SK snRNP upon Genotoxic Stress

As part of NEXT, RBM7 might tether RNA exosome to 7SK snRNP, resulting in its disintegration via 7SK nucleolysis. However, depletion of the NEXT subunits ZCCHC8 and MTR4, which link RBM7 with the core exosome (Falk et al., 2016; Lubas et al., 2011), failed to prevent P-TEFb activation upon DNA damage (Figure S3A). Further coIP and RIP-qPCR experiments confirmed that the 4-NQO-induced release of CDK9 and HEXIM1 from F-LARP7 left the core of 7SK snRNP intact. Specifically, F-LARP7 and MePCE remained bound during the DDR (Figure 4A), and neither the total nor the F-LARP7-bound levels of 7SK were altered considerably upon 4-NQO treatment (Figure 4B). Conversely, the levels of HEXIM1-bound 7SK decreased (Figure 4B). Demonstrating integrity of 7SK in the core, 4-NQO exposure increased the interaction of F-LARP7 with hnRNP A1 (Figure 4A). Namely, hnRNP A1 and other hnRNPs replace P-TEFb and HEXIM1 upon Pol II inhibition by binding SL3 of 7SK (Barrandon et al., 2007; Van Herreweghe et al., 2007). Unlike the reassembly of 7SK snRNP upon removing a P-TEFb inhibitor flavopiridol (FP) from cell culture medium (Figure S3B), the washout of 4-NQO did not induce resequestration of CDK9 and HEXIM1 into the 7SK snRNP, most likely due to the residual DNA damage as indicated by the persisting levels of phosphorylated γ-H2AX (Figure 4A). Thus, RBM7 does not employ the RNA exosome to activate P-TEFb.

To address if RBM7 could act on 7SK snRNP directly to release P-TEFb, we performed an *in vitro* P-TEFb release assay (Figure 4C). We immunopurified wild-type and mRNP1 F-RBM7 from HEK293 cells under stringent conditions to strip off its binding partners, including subunits of NEXT, RNA exosome, and P-TEFb (Figure 4D). Next, we incubated increasing amounts of the F-RBM7 proteins with the immobilized 7SK snRNP isolated from HeLa cells using an anti-HEXIM1 antibody. Indeed, F-RBM7 released CDK9 and 7SK from HEXIM1, while the mRNP1 F-RBM7 failed to do so (Figure 4D).

To uncover direct interactions between RBM7 and 7SK snRNP, we carried out *in vitro* pull-down assays with RBM7 and individual 7SK snRNP recombinant proteins. We purified wild-type and mRNP1 mutant maltose-binding protein (MBP)-tagged RBM7 (MBP-RBM7), glutathione-S-transferase (GST)-tagged HEXIM1, LARP7 and the catalytic domain of MePCE (cMePCE; residues 400–689), and P-TEFb composed of His-tagged CDK9 and GST-tagged cyclin-box domain of CycT1 (residues 1–272) to near-homogeneity using *E. coli* and baculovirus-infected *Sf9* insect cells (Figure S3C). We found that MBP-RBM7 interacted with GST-cMePCE and GST-LARP7 (Figures 4E and 4F) and that the 7SK-binding-deficient mRNP1 MBP-RBM7 retained the ability to bind these GST-tagged proteins (Figure S3D; data not shown). On the contrary, MBP-RBM7 failed to interact with the His-CDK9/GST-CycT1 heterodimer and GST-HEXIM1 (Figures 4G and S3E). Of note, we could not purify P-TEFb containing the full-length CycT1 of sufficient quality (data not

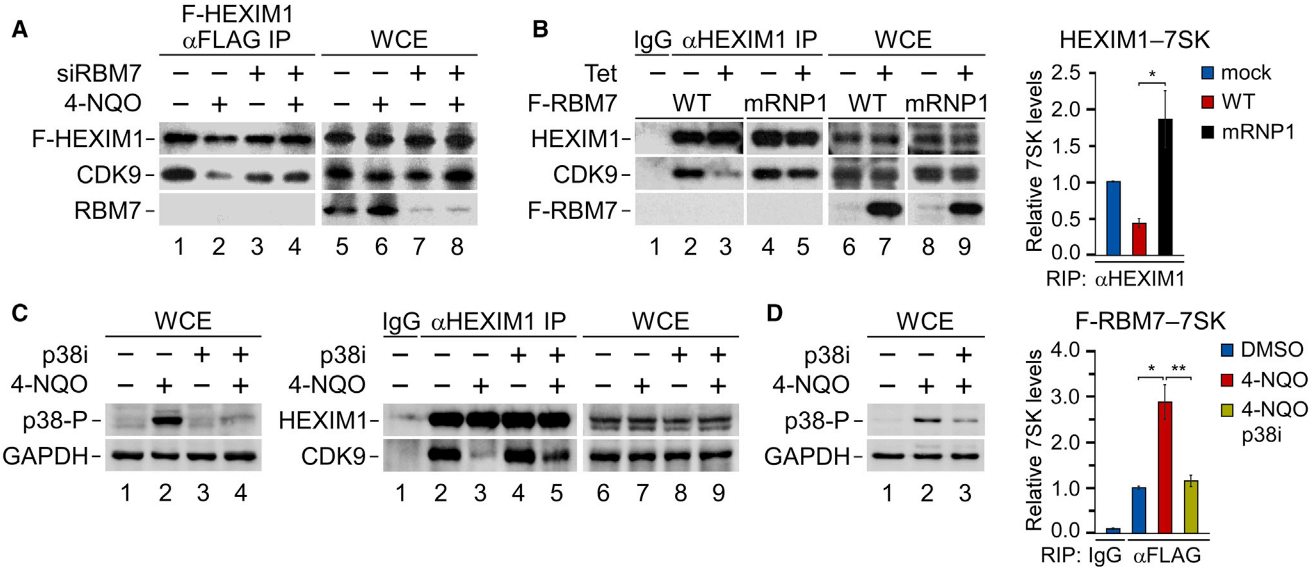


**Figure 3. RBM7 Is Critical for the Genotoxic-Stress-Induced Release of P-TEFb from HEXIM1**

(A) CoIP of F-HEXIM1 with CDK9 and RBM7 from WCE of HEK293 cells. Conditions with control (–) and RBM7 siRNA #1 (+) and with (+) and without (–) 4-NQO are shown.

(B) Left: CoIP of HEXIM1 with CDK9 from WCEs of HEK293 cells containing wild-type and mRNP1 F-RBM7. Conditions with (+) and without (–) F-RBM7 induction by tetracycline (Tet) are shown. Right: RIP-qPCR of 7SK in HEXIM1 IP from WCE of HEK293 cells containing wild-type and mRNP1 F-RBM7. Conditions with wild-type (red bars), mRNP1 (black bars), and without (blue bars) F-RBM7 induction by Tet are shown. Results are presented as the mean ± SEM (n = 3). *$p < 0.05$, determined by Student's t test.

(C) CoIP of HEXIM1 with CDK9 from WCEs of HeLa cells. Conditions with (+) and without (–) 4-NQO or p38i are shown. Levels of phospho-p38$^{MAPK}$ (p38-P) indicate activation of p38$^{MAPK}$.

(D) RIP-qPCR of 7SK in F-RBM7 IP from WCEs of HeLa cells. Conditions with 4-NQO (red bars), 4-NQO and p38i (yellow bars), and without 4-NQO (blue bars) are shown. Results are presented as the mean ± SEM (n = 3). *$p < 0.05$; **$p < 0.01$, determined by Student's t test. Levels of phospho-p38$^{MAPK}$ (p38-P) indicate activation of p38$^{MAPK}$.

shown) and were thus unable to evaluate its binding with RBM7. Together, these findings suggest that RBM7 activates P-TEFb in genotoxic-stressed cells via direct interactions with the core subunits of 7SK snRNP.

## P-TEFb Directs Transcriptional Activation by Pol II in Response to Genotoxic Stress

To disclose if active P-TEFb is vital for shaping a transcriptional response to genotoxic stress, we treated HeLa cells for 1 and 2 h with 4-NQO, labeled transcripts metabolically with the nucleoside analog 4-thiouridine (4sU) for 30 min, and isolated the newly transcribed RNAs for sequencing (4sU-seq) (Figure 5A). Concurrently with 4-NQO, we also exposed the cells to a suboptimal concentration of FP to evaluate if pharmacological inhibition of P-TEFb attenuates the response. Based on the reproducible 4sU-seq data (Figure S4A), we determined differentially expressed (DE) mRNAs, long intergenic non-coding RNAs (lincRNAs), uaRNAs, and eRNAs (p value $\leq$ 0.01; expression fold-change $\geq$ 2; Tables S2A–S2D). This revealed that over one-third of the DE coding genes and most of the DE ncRNAs are upregulated following genotoxic stress (Figures 5A and 5B). Importantly, the transcriptional changes, which were similar at both durations of 4-NQO treatments (r = 0.81 for eRNAs to 0.89 for uaRNAs; Figure S4B), were highly dependent on active P-TEFb (Figure 5B). Notably, the response to 4-NQO was rather specific, as only 10%–13% of mRNAs and uaRNAs and 3%–5% of lincRNAs and eRNAs underwent expression changes (Table S2E). In accordance with previous work on transcriptional response to UV (McKay et al., 2004; Williamson et al., 2017), the upregulated genes were considerably shorter than the downregulated genes (Figure 5C). Finally, p53, the bulky DNA adduct-inducers camptothecin and cisplatin, and DNA-damaging doxorubicin and sirolimus were identified by the Ingenuity Pathway Analysis (IPA) Upstream Regulator analytic as top regulators that could yield the 4-NQO-like response at coding genes (Table S2F), suggesting that DNA damage mediates the transcriptional changes.

To explore further the role of P-TEFb in DDR, we compiled a set of 4-NQO-induced coding genes that showed decreased expression by at least 20% upon FP co-treatment (4FP set; Table S3A). IPA identified cell death and survival, cellular development, cellular growth and proliferation, gene expression, and the cell cycle as the most significant cellular functions controlled by the 551-gene 4FP set (Figure 5D). Comparison with gene sets of the Molecular Signatures Database (MSigDB) collection revealed that the 4FP set bears similarity to the one controlled by NF-κB (p value = $6.01 \times 10^{-57}$) and of the p53 pathway (p value = $1.23 \times 10^{-40}$) (Table S3B) and is enriched for the reported sets of direct p53 target genes (Andrysik et al., 2017; Fischer, 2017) (p value = $2.65 \times 10^{-5}$ to $4.87 \times 10^{-10}$; Table S3C), suggesting

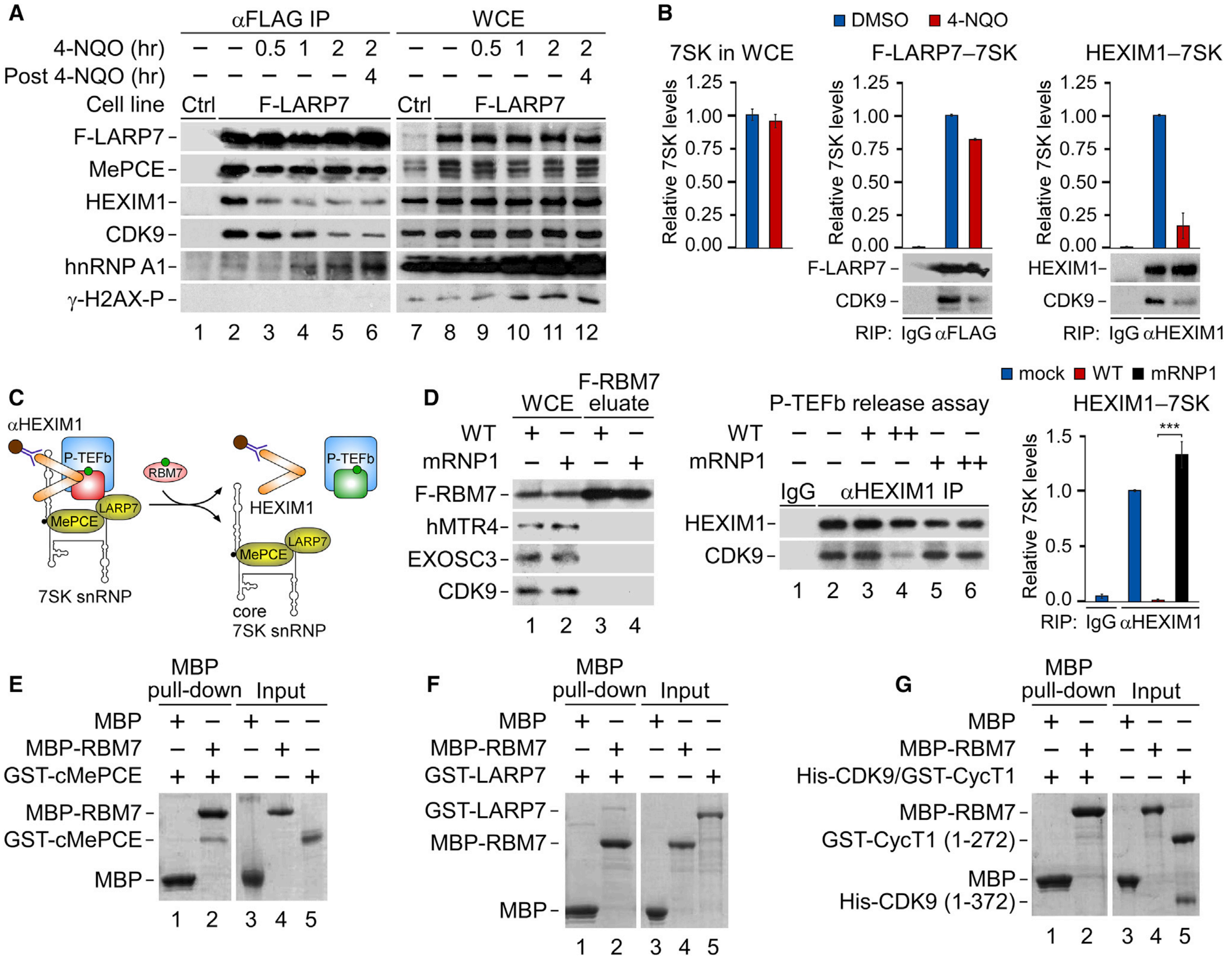

**Figure 4. RBM7 Releases P-TEFb from the Core of 7SK snRNP upon Genotoxic Stress**

(A) CoIP of F-LARP7 with 7SK snRNP and γ-H2AX from WCE of HEK293 cells. Conditions with (in hours) and without (–) 4-NQO are shown.

(B) RT-qPCR of 7SK (left) and RIP-qPCR of 7SK in F-LARP7 (middle) and HEXIM1 (right) IP from WCE of HEK293 cells. Conditions with (red bars) and without (blue bars) 4-NQO are shown. Results are presented as the mean ± SEM (n = 2). Protein levels in the IP are shown below the graphs.

(C) Cartoon depicting P-TEFb release assay. Release of inactive P-TEFb (CDK9 in red) from 7SK snRNP abrogates the P-TEFb–HEXIM1 interaction, resulting in P-TEFb activation (CDK9 in green).

(D) Left: eluates of immunopurified wild-type and mRNP1 F-RBM7 from HEK293 WCE. Middle: western blot analysis of P-TEFb release from HEXIM1 immunopurified (αHEXIM1 IP) 7SK snRNP by the F-RBM7 proteins. Control (–, lanes 1 and 2) and conditions with (+) increasing amounts of wild-type and mRNP1 F-RBM7 are shown. Right: RIP-qPCR of 7SK in HEXIM1 IP. Conditions with wild-type (red bars), mRNP1 (black bars), and without (blue bars) F-RBM7 incubation are shown. Results are presented as the mean ± SEM (n = 4). ***p < 0.001, determined by Student's t test.

(E–G) Coomassie-stained gels of *in vitro* MBP pull-down assays of MBP-RBM7 with GST-cMePCE (E), GST-LARP7 (F), and His-CDK9/GST-CycT1 (G).

See also Figure S3.

that these transcription factors (TFs) contribute to specifying the transcriptional response. Indeed, NF-κB and p53 function in DDR (Gomes et al., 2006; McKay et al., 2004; Sullivan et al., 2018; Wu et al., 2006) and require P-TEFb for activating their target genes (Barboric et al., 2001; Gomes et al., 2006). Furthermore, analysis of the sequences within 5kb from TSSs of the 4FP set genes with RcisTarget (Aibar et al., 2017) identified enriched motifs for many DNA-binding activators, including CCAAT-motif-binding NF-Y and CEBP TFs, cAMP-response-element-binding ATF/CREB family members, and TFs of the AP-1 family. (Figure S4C; Table S3D). Moreover, the 4FP set itself is enriched for nucleic-acid-binding proteins controlling gene expression (p value = $1.4 \times 10^{-16}$ to $4.36 \times 10^{-33}$; Table S3E), which might further shape the response. Finally, the 4FP set overlaps with the UV-induced gene sets of the MSigDB collection (p value = $3.8 \times 10^{-50}$ to $2.02 \times 10^{-56}$; Table S3F) and is predicted by IPA to respond to DNA damage-inducing agents (Table S3G).

We conducted kinetic qRT-PCR assays using HeLa cells to confirm that nascent transcripts of key functionally important DDR genes of the 4FP set, such as oncogenic *JUN*, *FOS*, and

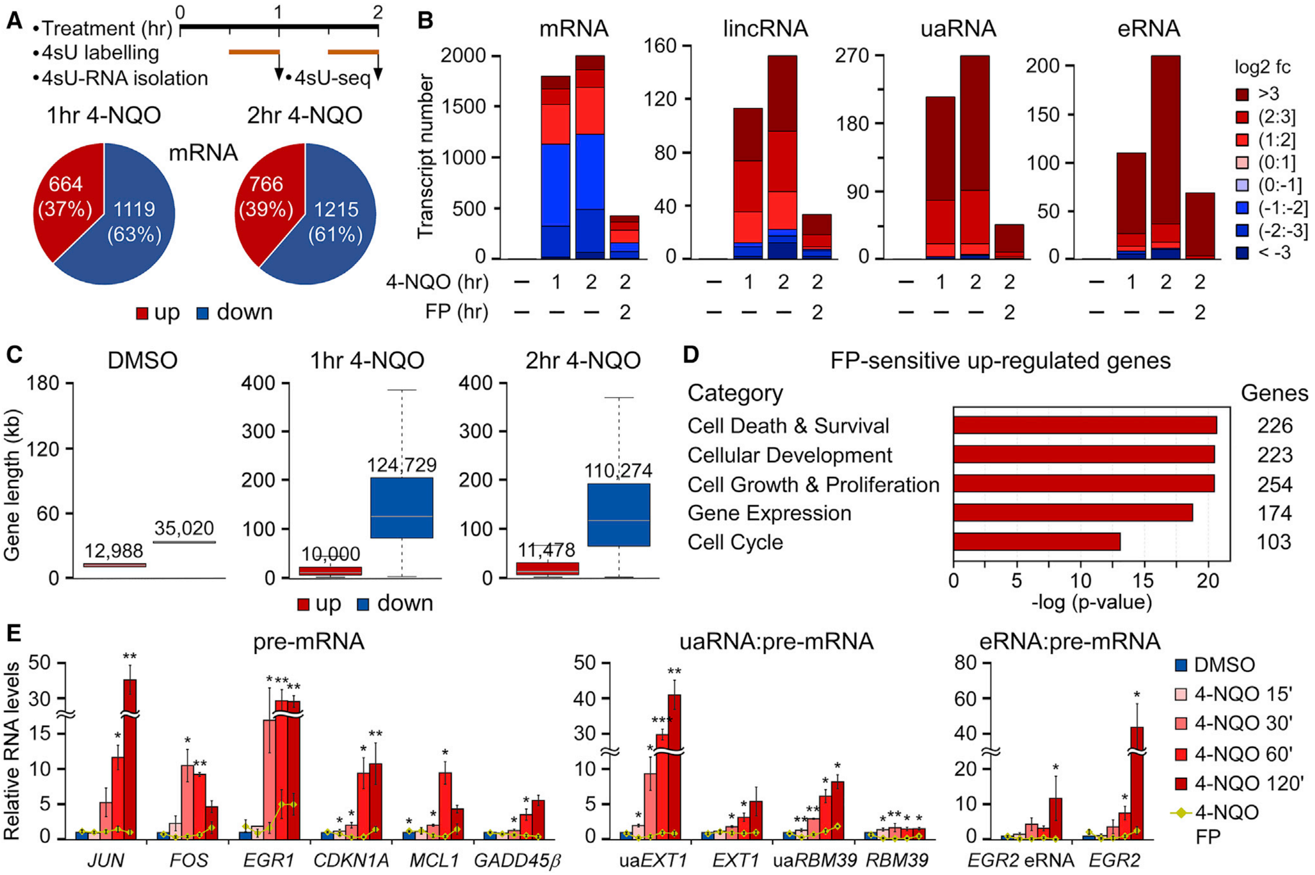


**Figure 5. Active P-TEFb Is Vital for the Pol II Transcriptional Response to Genotoxic Stress**

(A) (Top) Schematic depicting major steps in the generation of 4sU-labeled transcripts (4sU RNA) for 4sU-seq. (Bottom) Pie charts showing the fractions of DE protein-coding genes (mRNA) in 4-NQO-treated HeLa cells as assessed by 4sU-seq (n = 2).

(B) Bar charts showing the number of DE classes of transcripts in HeLa cells as assessed by 4sU-seq (n = 2). The degrees of differential expression are presented according to the legend. Conditions with (in hours) and without (–) 4-NQO or FP are shown.

(C) Boxplots indicating the distribution of gene lengths for upregulated and downregulated protein-coding genes. Median gene length for each group is shown.

(D) Top Molecular and Cellular Functions categories of the 4FP gene set as identified by IPA. The number of affected genes per category is shown on the right.

(E) RT-qPCR of the indicated DNA damage-induced unspliced (pre-mRNA), uaRNA, and eRNA transcripts. HeLa cells were treated as indicated by the legend. Results were normalized to the DMSO control and are presented as the mean ± SEM (n = 3). *p < 0.05; **p < 0.01; ***p < 0.001, determined by Student's t test. See also Figure S4 and Tables S3A–S3G.

*EGR1*, a cell-cycle inhibitor and a pro-survival *CDKN1A*, an anti-apoptotic *MCL1*, and a DDR regulator *GADD45*β, are upregulated upon DNA-damage-induced P-TEFb activation (Figure 5E). Importantly, co-administration of FP attenuated the induction of genes (Figure 5E). Similarly, the levels of oncogenic *EGR2* pre-mRNA and its eRNA, as well as those of *EXT1*, *RBM39*, and their corresponding uaRNAs, increased with a similar kinetic in a P-TEFb-dependent manner (Figure 5E). Together, these findings demonstrate that induced Pol II transcription of short protein-coding and non-coding genomic loci through P-TEFb is a hallmark of early DDR.

## RBM7 and 7SK snRNP Enable Induction of P-TEFb-Dependent DDR Genes

We next subjected the above protein-coding DDR genes to detailed mechanistic analyses. To provide further evidence that they are controlled by P-TEFb, we monitored the occupancy of CDK9 near TSSs of the seven genes by quantitative chromatin immunoprecipitation (ChIP-qPCR) assays using unchallenged and 4-NQO-treated HeLa cells. Furthermore, we followed the occupancy of total Pol II and its Ser2-P form near the TSS and in the middle of gene interior (INT) of each DDR gene. To account for the 4-NQO-triggered changes in Pol II occupancy at the genes, we normalized Ser2-P signals to those of total Pol II. In accordance with our biochemical and transcript findings, 4-NQO exposure elevated occupancy of CDK9 near the TSSs of nearly all tested DDR genes (Figures 6A and S5A). Concomitantly, levels of the Ser2-P hallmark of productive Pol II elongation also increased at the gene sites upon genotoxic stress, particularly near TSSs that correspond to the locations of paused Pol II (Figures 6A and S5A). Furthermore, FP blocked the increased Ser2-P levels (Figure S5B). The CDK9, Pol II, and

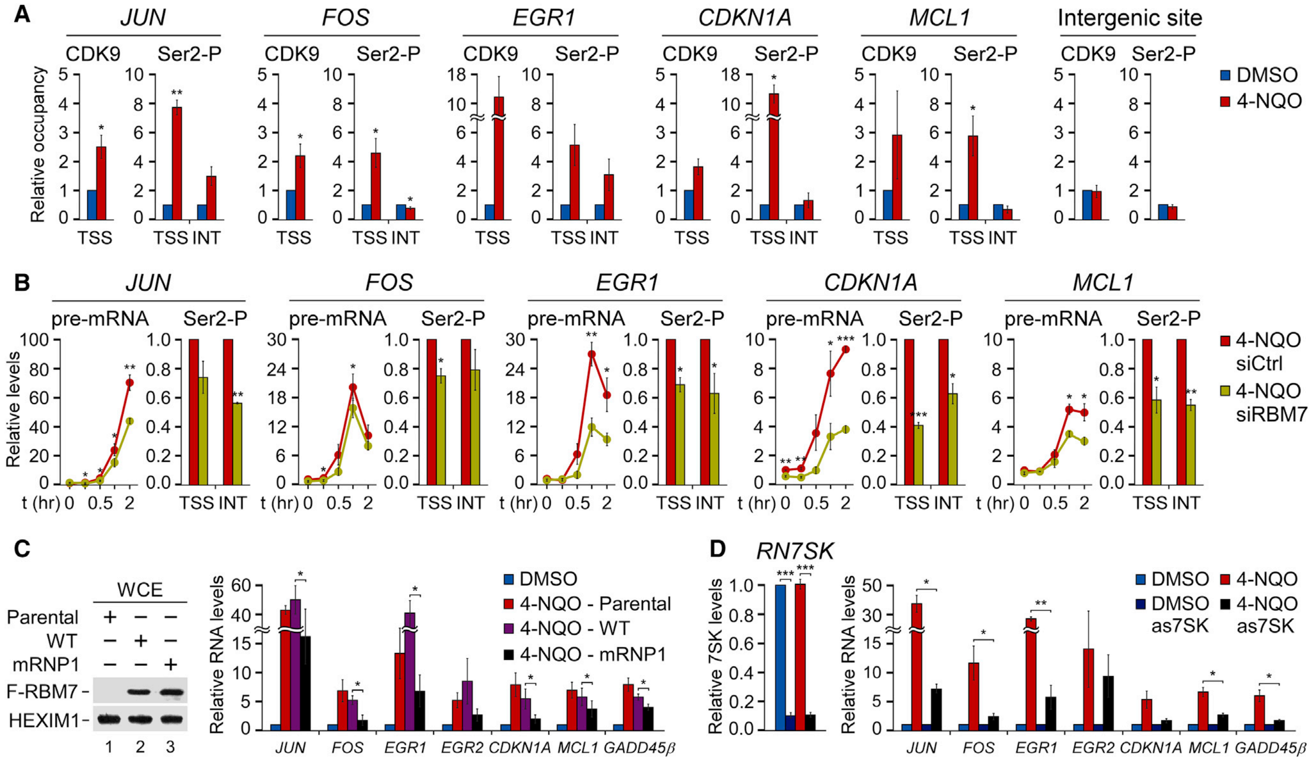


**Figure 6. RBM7 and 7SK snRNP Are Critical for the Induction of P-TEFb-Dependent DDR Genes**

(A) ChIP-qPCR of the occupancy of CDK9 and Ser2-P relative to Pol II at transcription start site (TSS) and in the middle of gene interior (INT) of the indicated DDR genes. The ChIP-qPCR data at the intergenic site ~100 kb upstream of *FOS* TSS is also presented. Conditions with (red bars) and without (blue bars) 4-NQO are shown. Results were normalized to the DMSO control and are presented as the mean ± SEM (n = 3). *p < 0.05; **p < 0.01, determined by Student's t test.

(B) RT-qPCR (left) of unspliced transcripts (pre-mRNA) of the indicated DDR genes and ChIP-qPCR (right) of the levels of Ser2-P relative to Pol II at the TSS and in the middle of gene interior (INT) of the indicated DDR genes in control (siCtrl; red) and *RBM7* knockdown (siRBM7 #2; yellow) 4-NQO-treated HeLa cells. In RT-qPCR assays, the cells were exposed to 4-NQO for 15 min, 0.5 h, 1 h, and 2 h as indicated, and results were normalized to the untreated control and are presented as the mean ± SEM (n = 3). *p < 0.05; **p < 0.01; ***p < 0.001, determined by Student's t test. ChIP-qPCR results were normalized to the control values that were set to 1 and are presented as the mean ± SEM (n = 3). *p < 0.05; **p < 0.01, determined by Student's t test.

(C and D) RT-qPCR of unspliced transcripts of the indicated DDR genes in parental, wild-type, and mRNP1 F-RBM7-expressing (C) or 7SK-depleted (D) HeLa cells. The cells were treated for 2 h with DMSO or 4-NQO as indicated by the legend. Results were normalized to the respective DMSO control and are presented as the mean ± SEM (C: n = 4; D: n = 3). *p < 0.05; **p < 0.01; ***p < 0.001, determined by Student's t test. Levels of the F-RBM7 proteins and efficacy of the *RN7SK* knockdown with the 7SK antisense DNA oligonucleotide (as7SK) are shown on the left.

See also Figure S5 and Table S4.

Ser2-P Pol II occupancies were specific, as they were enriched significantly over the normal immunoglobulin G (IgG) and *FOS* intergenic site controls (Table S4). These results highlight further the role of P-TEFb in stimulating Pol II transcription following 4-NQO exposure.

Our findings thus far support a model whereby genotoxic stress enhances the interaction of RBM7 with the core of 7SK snRNP, prompting the release of P-TEFb, which in turn stimulates transcription of specific gene sets. Thus, interfering with RBM7 or 7SK snRNP should hamper the 4-NQO-triggered gene induction. Indeed, kinetic qRT-PCR assays showed that *RBM7* knockdown decreased transcription at nearly all analyzed 4-NQO-induced genes in HeLa cells (Figures 6B and S5C). That the seven genes displayed varying degrees of transcriptional attenuation in the knockdown cells suggests that the RBM7–P-TEFb axis might be particularly vital for a subset of the induced transcriptome. Importantly, this transcriptional defect coincided with decreased levels of the Ser2-P mark at the induced genes as revealed by ChIP-qPCR experiments (Figures 6B and S5C), underscoring the role of RBM7 in P-TEFb-dependent gene induction. Supporting this notion, HeLa cells constitutively expressing the 7SK binding-deficient mRNP1 F-RBM7 exhibited decreased activation of these genes upon 4-NQO exposure when compared to the parental or F-RBM7 expressing HeLa cells (Figure 6C), suggesting that mRNP1 F-RBM7 acted in a dominant-negative fashion. Furthermore, inhibition of $p38^{MAPK}$, which interfered with the 4-NQO-induced P-TEFb release and RBM7–7SK interaction, also diminished the DDR gene induction (Figure S5D). In contrast, *ZCCHC8* and *MTR4* knockdown did not preclude the 4-NQO-triggered gene activation (Figures S5E and S5F). To monitor induction of the genes in cells with compromised 7SK snRNP, we used 7SK-specific phosphorothioate-modified antisense DNA oligodeoxynucleotide and found that depleting the scaffolding 7SK compromised the response to

genotoxic stress (Figure 6D). Given the predicted role of p53 in P-TEFb-dependent transcriptional response to 4-NQO (Tables S3B and S3C), we also examined the gene induction by 4-NQO using colorectal carcinoma HCT116 *TP53*$^{+/+}$ and HCT116 *TP53*$^{-/-}$ cell lines. Although the 4-NQO-triggered release of CDK9 from HEXIM1 took place in both lines (Figure S5G), the absence of p53 led to deficient transcription at specific genes, including its direct targets *FOS*, *EGR2*, and *CDKN1A* (Figure S5H). Together, these results confirm the vital role of the axis of RBM7 and P-TEFb and of cellular TFs in the transcriptional induction of important pro-survival and DDR genes.

## P-TEFb and RBM7 Promote Cell Viability upon Genotoxic Stress

The significance of RBM7 and P-TEFb in facilitating gene activation following genotoxic stress led us to hypothesize that antagonizing this transcriptional axis during DDR should be detrimental to cell survival. Consistent with this possibility, analysis of the 4FP set with the IPA Downstream Effects Analysis tool predicted that the induced P-TEFb-dependent genes promote cellular viability (Table S3H). Thus, we performed time course cytotoxicity assays in which compromised membrane integrity of dead cells is proportional to fluorescence signal generated by the binding of an otherwise cell impermeable cyanine dye to DNA. We used HeLa cells, a cell line derived from the retinal pigment epithelium (RPE-1), and primary HFF-1 cells. Of note, UV-induced stress leads to death or dysfunction of RPE-1 cells, which is thought to underlie several retinal diseases, including central vision loss of the age-related macular degeneration (Roduit and Schorderet, 2008). Confirming our hypothesis, HeLa, RPE-1, and HFF-1 cells became hypersensitive to 4-NQO when treated with the concentration of FP that attenuated the induction of P-TEFb-dependent genes, reaching death of nearly all cells at 36 and 72 h, respectively (Figures 7A and S6A). Depletion of RBM7 by two different small interfering RNAs (siRNAs) resulted in a similar hypersensitivity of HeLa and RPE-1 cell lines to 4-NQO and UV (Figures 7B, S6B, and S6C). Of note, ectopical expression of F-RBM7, which was resistant to the siRNA-mediated repression targeting the 3′ UTR of the endogenous *RBM7* transcripts, increased survival of 4-NQO-treated HeLa cells, underscoring the specificity of our RNAi findings (Figure S6D). To provide additional evidence for the pro-survival role of P-TEFb and RBM7 in genotoxic-stressed cells, we conducted fluorescence-based viability assays in which the indicator compound resazurin is reduced upon entering cells due to the reducing power of living cell cytosol, enabling its fluorescence capacity. Corroborating the cytotoxicity results, P-TEFb inhibition and RBM7 depletion decreased the viability of 4-NQO-exposed HeLa cells (Figures S6E and S6F).

Because of the contribution of p53 in P-TEFb-dependent gene induction following genotoxic stress (Figure S5H; Tables S3B and S3C), we also performed cytotoxicity assays using HCT116 *TP53*$^{+/+}$ and HCT116 *TP53*$^{-/-}$ cell lines. In contrast to the cells with functional p53, the cytotoxic response to P-TEFb inhibition with FP was markedly lowered in 4-NQO-treated HCT116 cells lacking p53 (Figure S6G), underscoring an important contribution of this TF to the pro-survival transcriptional program under P-TEFb control.

Finally, we asked if apoptosis was responsible for the hypersensitivity of P-TEFb- or RBM7-deficient cells to genotoxic stress. We followed kinetics of apoptosis using a luminescence-based assay that measures the exposure of phosphatidylserine on the outer cell membrane surface during apoptosis with two Annexin V fusion proteins containing fragments of NanoBit Luciferase (Dixon et al., 2016), of which complementation is achieved through binding of Annexin V and phosphatidylserine. Indeed, FP treatment and RBM7 depletion enhanced 4-NQO-stimulated apoptosis of HeLa cells (Figures 7C and 7D). Likewise, both perturbations increased 4-NQO-induced cleavage of poly(ADP-ribose) polymerase (PARP) and caspase-3 (Figures S6H and S6I), independently confirming apoptosis hyperactivation. Comparison of the cytotoxicity and apoptosis kinetics shows that the onset of apoptosis precedes the occurrence of cell death, indicating that apoptosis was causing the cellular demise. Together, these findings demonstrate the critical role of the transcriptional axis of RBM7 and P-TEFb in promoting viability of cells under genotoxic insult.

# DISCUSSION

In this study, we investigated the mechanisms and importance of transcriptional activation following genotoxic stress. We uncovered that stimulation of Pol II pause release by P-TEFb is at the heart of the cellular DDR (Figure 7E). We identified RBM7 as a critical regulator directing the stress-induced release of P-TEFb from the core of 7SK snRNP, which enables a transcriptional response that is indispensable to the survival of stressed cells. We envision that upon activation of P-TEFb, gene-specific TFs, including the known DDR players NF-κB and p53, as well as those predicted by our motifs analysis, capture the kinase on chromatin, thereby underwriting specificity of the transcriptional outcome and resolution to the genotoxic affront.

Our work describes an important paradigm in stress-dependent control of Pol II pause release. The role of RBM7 in the activation of P-TEFb expands its known regulatory ability, which has been thus far limited to promoting RNA degradation (Lubas et al., 2015). Considering that UV irradiation impairs the RBM7-mediated ribonucleolysis by triggering its phosphorylation at serines 136 and 204 via the p38$^{MAPK}$-MK2 pathway (Blasius et al., 2014; Tiedje et al., 2015), genotoxic stress thus switches a function of RBM7 from facilitating RNA degradation to promoting Pol II transcription. Of note, we did not find a role of MTR4 and ZCCHC8 in the activation of P-TEFb. However, because RBM7 binds both proteins at near-stoichiometric levels (Andersen et al., 2013; Lubas et al., 2011), and given our evidence suggesting the interaction of entire NEXT with 7SK snRNP, RBM7 likely activates P-TEFb as a subunit of NEXT. This possibility could explain the dominant-negative effect of the mRNP1 F-RBM7 on the induction of DDR genes, where the mutant protein might interfere with the function of endogenous RBM7 by dislodging it from NEXT. We speculate that P-TEFb activation is not limited to the DDR. It might take place also in response to other types of stress, such as hypoxic milieu of tumor cells, in which the oncogenic transcriptional activator HIF1A could co-opt activated P-TEFb for alleviating Pol II pausing at hypoxia-inducible genes (Galbraith et al., 2013).

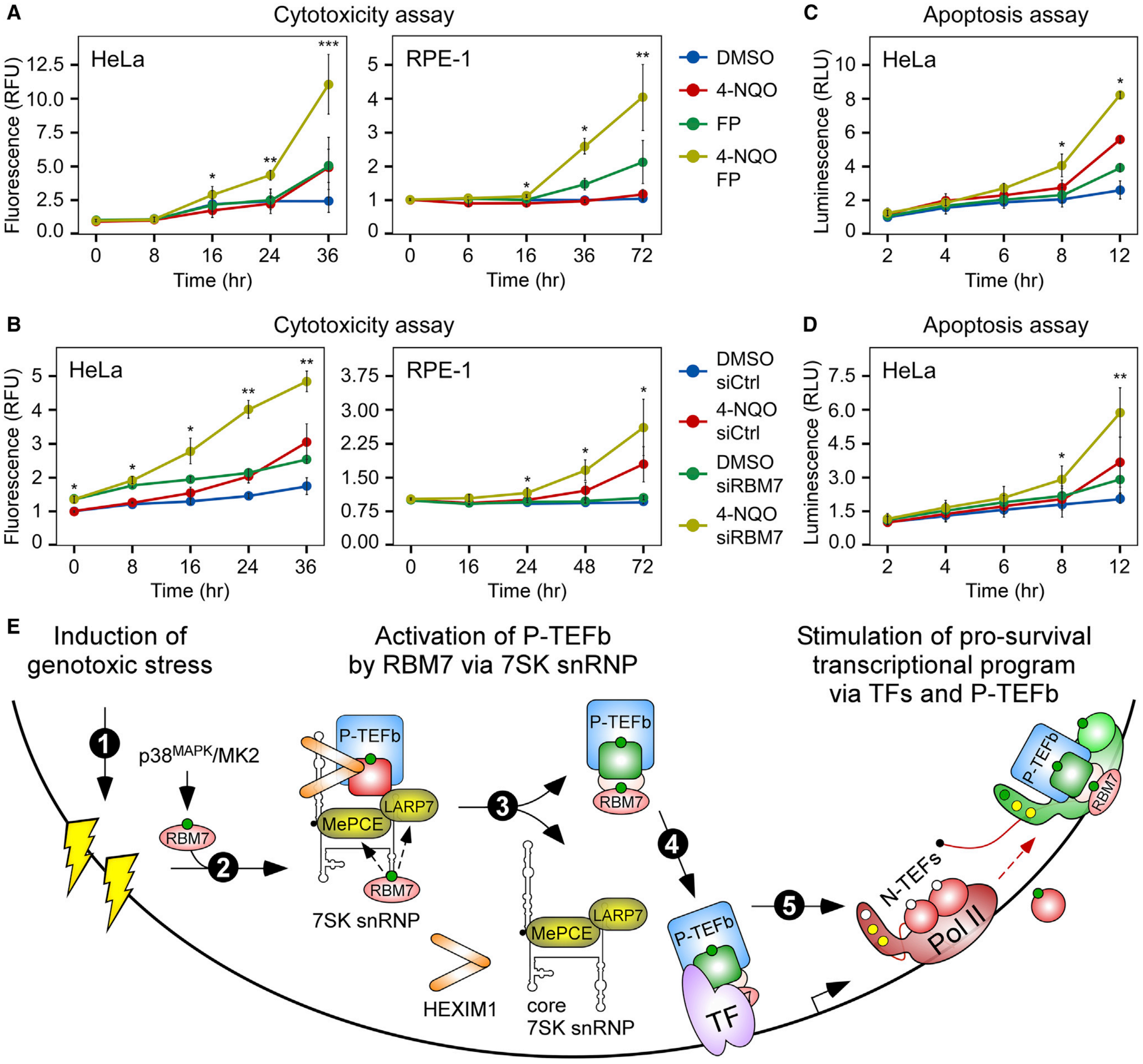


**Figure 7. P-TEFb and RBM7 Promote Cell Viability upon Genotoxic Stress**

(A and B) Hypersensitivity of HeLa and RPE-1 cells to 4-NQO upon FP treatment (A) and RBM7 depletion (B). The cells were treated as indicated by the legends and examined at the time points indicated below the graphs. Two independent siRNAs (siRBM7 #2, HeLa cells; siRBM7 #1, RPE-1 cells) were used to deplete RBM7. Cytotoxicity results are presented as fluorescence values relative to the untreated control and plotted as the mean ± SEM (n = 3). *$p < 0.05$; **$p < 0.01$; ***$p < 0.001$, determined by Student's t test using 4-NQO and 4-NQO FP or 4-NQO siRBM7 datasets, respectively.

(C and D) FP treatment (C) and RBM7 depletion (D) enhance 4-NQO-induced apoptosis in HeLa cells. The cells were treated as indicated by the legends and examined at the time points indicated below the graphs. siRBM7 #2 was used to deplete RBM7. Apoptosis results are presented as luminescence values relative to the untreated control and plotted as the mean ± SEM (n = 3). *$p < 0.05$; **$p < 0.01$, determined by Student's t test using 4-NQO and 4-NQO FP or 4-NQO siRBM7 datasets, respectively.

(E) Model of P-TEFb activation by RBM7 during DDR. Genotoxic stress (step 1) provokes phosphorylation (green circle) of RBM7 by the $p38^{MAPK}$-MK2 pathway. Subsequently, this triggers enhanced interaction of RBM7 with the core of 7SK snRNP (step 2; dashed arrows indicate interactions of RBM7 with MePCE and LARP7), triggering the release of inactive P-TEFb (CDK9 in red) from the core (step 3), yielding active P-TEFb (CDK9 in green). In turn, transcription factors (TFs) capture P-TEFb on chromatin (step 4). Stimulation of pro-survival DDR gene transcription at the Pol II pause release phase ensues (step 5), which is achieved by P-TEFb-mediated phosphorylation (green circles) of Pol II CTD at Ser2 as well as the negative transcription elongation factors (N-TEFs) NELF and DSIF. While NELF dissociates from Pol II, DSIF becomes a positive transcription elongation factor.

See also Figure S6 and Table S3H.

We uncover valuable insights into how RBM7 activates P-TEFb, which shall facilitate further studies of the mechanism at work. The lack of direct interaction of RBM7 with the P-TEFb containing cyclin-box domain of CycT1 and HEXIM1 argues against the possibility that RBM7 activates P-TEFb by dislodging the kinase from HEXIM1, as is the case in the HIV-1 Tat system (Barboric et al., 2007; Ott et al., 2011). Rather, based on our interaction findings, we propose that following genotoxic stress-induced activation of the $p38^{MAPK}$-MK2 pathway, direct binding of the phosphorylated RBM7 with the core of 7SK snRNP is a pre-requisite for the release of P-TEFb. Given that the RRM of RBM7 is dispensable for its binding with MePCE and LARP7, we speculate that the independent interacting surfaces in RBM7, consisting of the RRM and those likely formed upon phosphorylation of the serine residues within the C-terminal unstructured region of RBM7, mediate its induced binding with 7SK and the core 7SK snRNP protein subunits, respectively. Because the interaction of LARP7 with MePCE influences their affinity for 7SK and possibly the overall conformation of 7SK (Brogie and Price, 2017; Muniz et al., 2013; Xue et al., 2010), any structural changes of the core by RBM7 could trigger the release of HEXIM1 and P-TEFb from 7SK snRNP. The possible RBM7-induced conformational changes might also compromise the ability of LARP7 to promote the sequestration of P-TEFb into the snRNP (Markert et al., 2008; Muniz et al., 2013), contributing to P-TEFb activation.

Importantly, we highlight the significance of stimulating Pol II transcription upon DNA damage. Since P-TEFb targets Pol II that is poised for elongation, its activation and chromatin recruitment is suited uniquely to promote the rapid and coordinated transcriptional response. Our biochemical and gene induction findings are in agreement with the reported activation of transcription at promoter-proximal regions following UV irradiation (Andrade-Lima et al., 2015; Lavigne et al., 2017; Williamson et al., 2017) and with previous studies that linked the activation of P-TEFb with the DDR (Gudipaty et al., 2015; Nguyen et al., 2001; Yang et al., 2001). It remains to be established, however, how exactly the stimulation of Pol II pause release promotes survival of cells under genotoxic stress. We propose that transcriptional reprogramming plays a pivotal role. While induction of coding DDR genes as exemplified by *CDKN1A* and *MCL1* as well as lincRNAs enables cell-cycle arrest and opposes apoptosis (Cazzalini et al., 2010; Huarte et al., 2010; Zhou et al., 1997), elevated levels of uaRNAs and eRNAs might concurrently stabilize new gene expression program through various mechanisms, including modulation of chromatin, promotion of TFs occupancy, and augmentation of Pol II elongation (Kaikkonen and Adelman, 2018; Schaukowitch et al., 2014). Accordingly, we speculate that the transiently reshaped transcriptome sets the stage for subsequent recovery from the stress. Therein activation of P-TEFb could be also advantageous to the repair of damaged DNA. Indeed, stimulating Pol II pause release facilitates transcription-coupled NER (TC-NER) across the genome (Andrade-Lima et al., 2015; Chiou et al., 2018; Lavigne et al., 2017). Namely, Pol II stalling at the sites of DNA damage mediates activation of this pathway (Vermeulen and Fousteri, 2013), and because Pol II dissociates from DNA during individual TC-NER reactions (Chiou et al., 2018), the repair of subsequent DNA lesions would benefit from iterative rounds of Pol II pause release events. Active P-TEFb might also exert its positive role via its DNA damage-induced interaction with Cockayne syndrome B translocase (Boeing et al., 2016), which is instrumental for TC-NER and resetting of transcription as cells recover from the damage (Epanchintsev et al., 2017).

Finally, P-TEFb-dependent Pol II pause release is frequently dysregulated in cancers, particularly in those addicted to c-MYC and translocations of mixed-lineage leukemia gene (Dawson et al., 2011; Delmore et al., 2011; Liang et al., 2018), spurring interest in the development of highly specific CDK9 inhibitors for clinical use (Lu et al., 2015; Olson et al., 2018). Our discovery of the pro-survival role of P-TEFb in genotoxic stress reinforces the relevance of this key transcriptional kinase to cancer biology. Hence, we offer a framework for anti-cancer approaches consisting of targeting RBM7 and P-TEFb together with DNA-damaging chemotherapeutics. Countering the axis of RBM7 and P-TEFb might prove especially effectual in combination with bulky DNA-adduct-inducing platinum-based drugs such as cisplatin, which shows improved therapeutic outcome in NER-deficient cancers (Dietlein et al., 2014; Gavande et al., 2016). The proposed combinatorial approach might also curb the emergence of drug-resistant tumor cells, a major challenge of contemporary cancer monotherapies.

## STAR★METHODS

Detailed methods are provided in the online version of this paper and include the following:

- KEY RESOURCES TABLE
- CONTACT FOR REAGENT AND RESOURCE SHARING
- EXPERIMENTAL MODEL AND SUBJECT DETAILS
- METHOD DETAILS
  - Plasmid DNAs and Mutagenesis
  - Chemicals and Treatments
  - RBM7 iCLIP Assay
  - Quantitative RNA Immunoprecipitation Assay
  - Co-immunoprecipitation and Western Blotting Assays
  - Glycerol Gradient Sedimentation Analysis
  - Immunofluorescence Microscopy
  - Bimolecular Fluorescence Complementation Assay
  - P-TEFb Release Assay
  - Expression of Recombinant Proteins
  - Purification of Recombinant Proteins
  - MBP-pull down Assay
  - 4sU-sequencing Assay
  - Analysis of 4sU-sequencing Datasets
  - Ingenuity Pathway Analysis and Molecular Signature Database Analysis
  - Transcription Factor Binding Motifs Analysis
  - p53 Target Gene Enrichment Analysis
  - RNA extraction and RT-qPCR Analysis
  - Quantitative Chromatin Immunoprecipitation Assay
  - RNA Interference, siRNAs and antisense-oligonucleotide treatment
  - Cytotoxicity Assay

- Viability Assay
- Apoptosis Assay
- QUANTIFICATION AND STATISTICAL ANALYSIS
- DATA AND SOFTWARE AVAILABILITY
  - Software
  - Data Resources

## SUPPLEMENTAL INFORMATION

Supplemental Information can be found with this article online at https://doi.org/10.1016/j.molcel.2019.01.033.

## ACKNOWLEDGMENTS

We thank Céline Verheggen and Edouard Bertrand for sharing the HeLa Flp-In cell line; Masami Nagahama for sharing HEK 293 Flp-In T-REx F-hMTR4 cell line; Zdenek Andrysik, Joaquin M. Espinosa, and Bert Vogelstein for sharing HCT116 *TP53*$^{+/+}$ and HCT116 *TP53*$^{-/-}$ cell lines; Qiang Zhou for sharing anti-LARP7 antibody; Michal Lubas for providing uaRNA and eRNA annotations; Stephen P. Jackson for advice and suggestions; Zeping Luo for technical assistance; and Denis Belitskin and Karlo Skube for sharing several commercially available antibodies throughout the study. This work was funded by the Academy of Finland (grants 1263825 and 1309846 to M.B.), the Sigrid Jusélius Foundation (grant 4704610 to M.B.), the Deutsche Forschungsgemeinschaft (DFG) (grant Do-1275/2-1 to L.D., grant GE-976/9-2 to M.G., and grant FR2938/7-1 to C.C.F.), the European Research Council (grant 206726-CLIP to J.U.), the Francis Crick Institute, with core funding from Cancer Research UK (grant FC001002), the UK Medical Research Council (grant FC001002), and the Wellcome Trust (grant FC001002 to J.U.). A.B. was supported in part by funding from the Doctoral Program in Biomedicine, University of Helsinki. A.J.C.Q. was supported in part by the K. Albin Johansson Foundation and by the The Ella and Georg Ehrnrooth Foundation. C.R.S. was supported by an Edmond Lily Safra fellowship.

## AUTHOR CONTRIBUTIONS

M. Barborič conceptualized and supervised the study and was assisted by A.B. and A.J.C.Q. in experimental design. C.R.S. performed iCLIP with the assistance from M. Blasius and analyzed iCLIP data with assistance from J.U. 4sU-seq was performed by A.B., T.H., and L.D., and C.C.F. analyzed 4sU-seq data. R.D. purified recombinant proteins and performed binding experiments with supervision by M.G. V-PAC experiments were performed by K.F. All other experiments were performed by A.B., A.J.C.Q, T.L., and P.K., where A.B. contributed the most. M. Barborič wrote the manuscript with input from L.D., J.U., M.G., and A.B.

## DECLARATION OF INTERESTS

The authors declare no competing interests.

Received: June 13, 2018
Revised: November 27, 2018
Accepted: January 23, 2019
Published: February 26, 2019

## REFERENCES

Aibar, S., González-Blas, C.B., Moerman, T., Huynh-Thu, V.A., Imrichova, H., Hulselmans, G., Rambow, F., Marine, J.C., Geurts, P., Aerts, J., et al. (2017). SCENIC: single-cell regulatory network inference and clustering. Nat. Methods *14*, 1083–1086.

Andersen, P.R., Domanski, M., Kristiansen, M.S., Storvall, H., Ntini, E., Verheggen, C., Schein, A., Bunkenborg, J., Poser, I., Hallais, M., et al. (2013). The human cap-binding complex is functionally connected to the nuclear RNA exosome. Nat. Struct. Mol. Biol. *20*, 1367–1376.

Andrade-Lima, L.C., Veloso, A., Paulsen, M.T., Menck, C.F., and Ljungman, M. (2015). DNA repair and recovery of RNA synthesis following exposure to ultraviolet light are delayed in long genes. Nucleic Acids Res. *43*, 2744–2756.

Andrysik, Z., Galbraith, M.D., Guarnieri, A.L., Zaccara, S., Sullivan, K.D., Pandey, A., MacBeth, M., Inga, A., and Espinosa, J.M. (2017). Identification of a core TP53 transcriptional program with highly distributed tumor suppressive activity. Genome Res. *27*, 1645–1657.

Awwad, S.W., Abu-Zhayia, E.R., Guttmann-Raviv, N., and Ayoub, N. (2017). NELF-E is recruited to DNA double-strand break sites to promote transcriptional repression and repair. EMBO Rep. *18*, 745–764.

Bacon, C.W., and D'Orso, I. (2018). CDK9: a signaling hub for transcriptional control. Transcription. Published online September 19, 2018. https://doi.org/10.1080/21541264.2018.

Barboric, M., Nissen, R.M., Kanazawa, S., Jabrane-Ferrat, N., and Peterlin, B.M. (2001). NF-kappaB binds P-TEFb to stimulate transcriptional elongation by RNA polymerase II. Mol. Cell *8*, 327–337.

Barboric, M., Yik, J.H., Czudnochowski, N., Yang, Z., Chen, R., Contreras, X., Geyer, M., Matija Peterlin, B., and Zhou, Q. (2007). Tat competes with HEXIM1 to increase the active pool of P-TEFb for HIV-1 transcription. Nucleic Acids Res. *35*, 2003–2012.

Barrandon, C., Bonnet, F., Nguyen, V.T., Labas, V., and Bensaude, O. (2007). The transcription-dependent dissociation of P-TEFb-HEXIM1-7SK RNA relies upon formation of hnRNP-7SK RNA complexes. Mol. Cell. Biol. *27*, 6996–7006.

Bartholomeeusen, K., Xiang, Y., Fujinaga, K., and Peterlin, B.M. (2012). Bromodomain and extra-terminal (BET) bromodomain inhibition activate transcription via transient release of positive transcription elongation factor b (P-TEFb) from 7SK small nuclear ribonucleoprotein. J. Biol. Chem. *287*, 36609–36616.

Benjamini, Y., and Hochberg, Y. (1995). Controlling the false discovery rate: a practical and powerful approach to multiple testing. J. R. Stat. Soc. Series B Stat. Methodol. *57*, 289–300.

Blasius, M., Wagner, S.A., Choudhary, C., Bartek, J., and Jackson, S.P. (2014). A quantitative 14-3-3 interaction screen connects the nuclear exosome targeting complex to the DNA damage response. Genes Dev. *28*, 1977–1982.

Boeing, S., Williamson, L., Encheva, V., Gori, I., Saunders, R.E., Instrell, R., Aygün, O., Rodriguez-Martinez, M., Weems, J.C., Kelly, G.P., et al. (2016). Multiomic analysis of the UV-induced DNA damage response. Cell Rep. *15*, 1597–1610.

Bonfert, T., Kirner, E., Csaba, G., Zimmer, R., and Friedel, C.C. (2015). ContextMap 2: fast and accurate context-based RNA-seq mapping. BMC Bioinformatics *16*, 122.

Borisova, M.E., Voigt, A., Tollenaere, M.A.X., Sahu, S.K., Juretschke, T., Kreim, N., Mailand, N., Choudhary, C., Bekker-Jensen, S., Akutsu, M., et al. (2018). p38-MK2 signaling axis regulates RNA metabolism after UV-light-induced DNA damage. Nat. Commun. *9*, 1017.

Brogie, J.E., and Price, D.H. (2017). Reconstitution of a functional 7SK snRNP. Nucleic Acids Res. *45*, 6864–6880.

Quaresma, A.J.C., Bugai, A., and Barboric, M. (2016). Cracking the control of RNA polymerase II elongation by 7SK snRNP and P-TEFb. Nucleic Acids Res. *44*, 7527–7539.

Calo, E., Flynn, R.A., Martin, L., Spitale, R.C., Chang, H.Y., and Wysocka, J. (2015). RNA helicase DDX21 coordinates transcription and ribosomal RNA processing. Nature *518*, 249–253.

Cazzalini, O., Scovassi, A.I., Savio, M., Stivala, L.A., and Prosperi, E. (2010). Multiple roles of the cell cycle inhibitor p21(CDKN1A) in the DNA damage response. Mutat. Res. *704*, 12–20.

Chiou, Y.Y., Hu, J., Sancar, A., and Selby, C.P. (2018). RNA polymerase II is released from the DNA template during transcription-coupled repair in mammalian cells. J. Biol. Chem. *293*, 2476–2486.

Contreras, X., Schweneker, M., Chen, C.S., McCune, J.M., Deeks, S.G., Martin, J., and Peterlin, B.M. (2009). Suberoylanilide hydroxamic acid reactivates HIV from latently infected cells. J. Biol. Chem. *284*, 6782–6789.

Dawson, M.A., Prinjha, R.K., Dittmann, A., Giotopoulos, G., Bantscheff, M., Chan, W.I., Robson, S.C., Chung, C.W., Hopf, C., Savitski, M.M., et al. (2011). Inhibition of BET recruitment to chromatin as an effective treatment for MLL-fusion leukaemia. Nature *478*, 529–533.

Delmore, J.E., Issa, G.C., Lemieux, M.E., Rahl, P.B., Shi, J., Jacobs, H.M., Kastritis, E., Gilpatrick, T., Paranal, R.M., Qi, J., et al. (2011). BET bromodomain inhibition as a therapeutic strategy to target c-Myc. Cell *146*, 904–917.

Dietlein, F., Thelen, L., and Reinhardt, H.C. (2014). Cancer-specific defects in DNA repair pathways as targets for personalized therapeutic approaches. Trends Genet. *30*, 326–339.

Dixon, A.S., Schwinn, M.K., Hall, M.P., Zimmerman, K., Otto, P., Lubben, T.H., Butler, B.L., Binkowski, B.F., Machleidt, T., Kirkland, T.A., et al. (2016). NanoLuc complementation reporter optimized for accurate measurement of protein interactions in cells. ACS Chem. Biol. *11*, 400–408.

Dobin, A., Davis, C.A., Schlesinger, F., Drenkow, J., Zaleski, C., Jha, S., Batut, P., Chaisson, M., and Gingeras, T.R. (2013). STAR: ultrafast universal RNA-seq aligner. Bioinformatics *29*, 15–21.

Dutertre, M., Lambert, S., Carreira, A., Amor-Guéret, M., and Vagner, S. (2014). DNA damage: RNA-binding proteins protect from near and far. Trends Biochem. Sci. *39*, 141–149.

Ekumi, K.M., Paculova, H., Lenasi, T., Pospichalova, V., Bösken, C.A., Rybarikova, J., Bryja, V., Geyer, M., Blazek, D., and Barboric, M. (2015). Ovarian carcinoma CDK12 mutations misregulate expression of DNA repair genes via deficient formation and function of the Cdk12/CycK complex. Nucleic Acids Res. *43*, 2575–2589.

Epanchintsev, A., Costanzo, F., Rauschendorf, M.A., Caputo, M., Ye, T., Donnio, L.M., Proietti-de-Santis, L., Coin, F., Laugel, V., and Egly, J.M. (2017). Cockayne's syndrome A and B proteins regulate transcription arrest after genotoxic stress by promoting ATF3 degradation. Mol. Cell *68*, 1054–1066.e1056.

Falk, S., Finogenova, K., Melko, M., Benda, C., Lykke-Andersen, S., Jensen, T.H., and Conti, E. (2016). Structure of the RBM7-ZCCHC8 core of the NEXT complex reveals connections to splicing factors. Nat. Commun. *7*, 13573.

Fischer, M. (2017). Census and evaluation of p53 target genes. Oncogene *36*, 3943–3956.

Fujinaga, K., Luo, Z., Schaufele, F., and Peterlin, B.M. (2015). Visualization of positive transcription elongation factor b (P-TEFb) activation in living cells. J. Biol. Chem. *290*, 1829–1836.

Galbraith, M.D., Allen, M.A., Bensard, C.L., Wang, X., Schwinn, M.K., Qin, B., Long, H.W., Daniels, D.L., Hahn, W.C., Dowell, R.D., and Espinosa, J.M. (2013). HIF1A employs CDK8-mediator to stimulate RNAPII elongation in response to hypoxia. Cell *153*, 1327–1339.

Gavande, N.S., VanderVere-Carozza, P.S., Hinshaw, H.D., Jalal, S.I., Sears, C.R., Pawelczak, K.S., and Turchi, J.J. (2016). DNA repair targeted therapy: the past or future of cancer treatment? Pharmacol. Ther. *160*, 65–83.

Giono, L.E., Nieto Moreno, N., Cambindo Botto, A.E., Dujardin, G., Muñoz, M.J., and Kornblihtt, A.R. (2016). The RNA response to DNA damage. J. Mol. Biol. *428*, 2636–2651.

Gomes, N.P., Bjerke, G., Llorente, B., Szostek, S.A., Emerson, B.M., and Espinosa, J.M. (2006). Gene-specific requirement for P-TEFb activity and RNA polymerase II phosphorylation within the p53 transcriptional program. Genes Dev. *20*, 601–612.

Gressel, S., Schwalb, B., Decker, T.M., Qin, W., Leonhardt, H., Eick, D., and Cramer, P. (2017). CDK9-dependent RNA polymerase II pausing controls transcription initiation. eLife *6*, e29736.

Gudipaty, S.A., McNamara, R.P., Morton, E.L., and D'Orso, I. (2015). PPM1G binds 7SK RNA and Hexim1 to block P-TEFb assembly into the 7SK snRNP and sustain transcription elongation. Mol. Cell. Biol. *35*, 3810–3828.

He, N., Jahchan, N.S., Hong, E., Li, Q., Bayfield, M.A., Maraia, R.J., Luo, K., and Zhou, Q. (2008). A La-related protein modulates 7SK snRNP integrity to suppress P-TEFb-dependent transcriptional elongation and tumorigenesis. Mol. Cell *29*, 588–599.

Hallais, M., Pontvianne, F., Andersen, P.R., Clerici, M., Lener, D., Benbahouche, Nel.H., Gostan, T., Vandermoere, F., Robert, M.C., Cusack, S., et al. (2013). CBC-ARS2 stimulates 3′-end maturation of multiple RNA families and favors cap-proximal processing. Nat. Struct. Mol. Biol. *20*, 1358–1366.

Hiraishi, N., Ishida, Y., and Nagahama, M. (2015). AAA-ATPase NVL2 acts on MTR4-exosome complex to dissociate the nucleolar protein WDR74. Biochem. Biophys. Res. Commun. *467*, 534–540.

Hoeijmakers, J.H. (2009). DNA damage, aging, and cancer. N. Engl. J. Med. *361*, 1475–1485.

Huarte, M., Guttman, M., Feldser, D., Garber, M., Koziol, M.J., Kenzelmann-Broz, D., Khalil, A.M., Zuk, O., Amit, I., Rabani, M., et al. (2010). A large intergenic noncoding RNA induced by p53 mediates global gene repression in the p53 response. Cell *142*, 409–419.

Huppertz, I., Attig, J., D'Ambrogio, A., Easton, L.E., Sibley, C.R., Sugimoto, Y., Tajnik, M., König, J., and Ule, J. (2014). iCLIP: protein-RNA interactions at nucleotide resolution. Methods *65*, 274–287.

Ikenaga, M., Takebe, H., and Ishii, Y. (1977). Excision repair of DNA base damage in human cells treated with the chemical carcinogen 4-nitroquinoline 1-oxide. Mutat. Res. *43*, 415–427.

Jackson, S.P., and Bartek, J. (2009). The DNA-damage response in human biology and disease. Nature *461*, 1071–1078.

Kaikkonen, M.U., and Adelman, K. (2018). Emerging roles of non-coding RNA transcription. Trends Biochem. Sci. *43*, 654–667.

Konig, J., Zarnack, K., Rot, G., Curk, T., Kayikci, M., Zupan, B., Turner, D.J., Luscombe, N.M., and Ule, J. (2010). iCLIP reveals the function of hnRNP particles in splicing at individual nucleotide resolution. Nat. Struct. Mol. Biol. *17*, 909–915.

Lavigne, M.D., Konstantopoulos, D., Ntakou-Zamplara, K.Z., Liakos, A., and Fousteri, M. (2017). Global unleashing of transcription elongation waves in response to genotoxic stress restricts somatic mutation rate. Nat. Commun. *8*, 2076.

Liang, K., Smith, E.R., Aoi, Y., Stoltz, K.L., Katagi, H., Woodfin, A.R., Rendleman, E.J., Marshall, S.A., Murray, D.C., Wang, L., et al. (2018). Targeting processive transcription elongation via sec disruption for myc-induced cancer therapy. Cell *175*, 766–779.e717.

Liao, Y., Smyth, G.K., and Shi, W. (2014). featureCounts: an efficient general purpose program for assigning sequence reads to genomic features. Bioinformatics *30*, 923–930.

Liu, X., Kraus, W.L., and Bai, X. (2015). Ready, pause, go: regulation of RNA polymerase II pausing and release by cellular signaling pathways. Trends Biochem. Sci. *40*, 516–525.

Lu, H., Xue, Y., Yu, G.K., Arias, C., Lin, J., Fong, S., Faure, M., Weisburd, B., Ji, X., Mercier, A., et al. (2015). Compensatory induction of MYC expression by sustained CDK9 inhibition via a BRD4-dependent mechanism. eLife *4*, e06535.

Lu, H., Yu, D., Hansen, A.S., Ganguly, S., Liu, R., Heckert, A., Darzacq, X., and Zhou, Q. (2018). Phase-separation mechanism for C-terminal hyperphosphorylation of RNA polymerase II. Nature *558*, 318–323.

Lubas, M., Christensen, M.S., Kristiansen, M.S., Domanski, M., Falkenby, L.G., Lykke-Andersen, S., Andersen, J.S., Dziembowski, A., and Jensen, T.H. (2011). Interaction profiling identifies the human nuclear exosome targeting complex. Mol. Cell *43*, 624–637.

Lubas, M., Andersen, P.R., Schein, A., Dziembowski, A., Kudla, G., and Jensen, T.H. (2015). The human nuclear exosome targeting complex is loaded onto newly synthesized RNA to direct early ribonucleolysis. Cell Rep. *10*, 178–192.

Markert, A., Grimm, M., Martinez, J., Wiesner, J., Meyerhans, A., Meyuhas, O., Sickmann, A., and Fischer, U. (2008). The La-related protein LARP7 is a component of the 7SK ribonucleoprotein and affects transcription of cellular and viral polymerase II genes. EMBO Rep. *9*, 569–575.

Martin, M. (2011). Cutadapt removes adapter sequences from high-throughput sequencing reads. Embnet J. *17*, 10–12.

McKay, B.C., Stubbert, L.J., Fowler, C.C., Smith, J.M., Cardamore, R.A., and Spronck, J.C. (2004). Regulation of ultraviolet light-induced gene expression by gene size. Proc. Natl. Acad. Sci. USA *101*, 6582–6586.

Michels, A.A., and Bensaude, O. (2018). Hexim1, an RNA-controlled protein hub. Transcription *9*, 262–271.

Muniz, L., Egloff, S., and Kiss, T. (2013). RNA elements directing in vivo assembly of the 7SK/MePCE/Larp7 transcriptional regulatory snRNP. Nucleic Acids Res. *41*, 4686–4698.

Nguyen, V.T., Kiss, T., Michels, A.A., and Bensaude, O. (2001). 7SK small nuclear RNA binds to and inhibits the activity of CDK9/cyclin T complexes. Nature *414*, 322–325.

Olson, C.M., Jiang, B., Erb, M.A., Liang, Y., Doctor, Z.M., Zhang, Z., Zhang, T., Kwiatkowski, N., Boukhali, M., Green, J.L., et al. (2018). Pharmacological perturbation of CDK9 using selective CDK9 inhibition or degradation. Nat. Chem. Biol. *14*, 163–170.

Ott, M., Geyer, M., and Zhou, Q. (2011). The control of HIV transcription: keeping RNA polymerase II on track. Cell Host Microbe *10*, 426–435.

Rädle, B., Rutkowski, A.J., Ruzsics, Z., Friedel, C.C., Koszinowski, U.H., and Dölken, L. (2013). Metabolic labeling of newly transcribed RNA for high resolution gene expression profiling of RNA synthesis, processing and decay in cell culture. J. Vis. Exp. (78), 50195.

Robinson, M.D., McCarthy, D.J., and Smyth, G.K. (2010). edgeR: a Bioconductor package for differential expression analysis of digital gene expression data. Bioinformatics *26*, 139–140.

Rockx, D.A., Mason, R., van Hoffen, A., Barton, M.C., Citterio, E., Bregman, D.B., van Zeeland, A.A., Vrieling, H., and Mullenders, L.H. (2000). UV-induced inhibition of transcription involves repression of transcription initiation and phosphorylation of RNA polymerase II. Proc. Natl. Acad. Sci. USA *97*, 10503–10508.

Roduit, R., and Schorderet, D.F. (2008). MAP kinase pathways in UV-induced apoptosis of retinal pigment epithelium ARPE19 cells. Apoptosis *13*, 343–353.

Schaukowitch, K., Joo, J.Y., Liu, X., Watts, J.K., Martinez, C., and Kim, T.K. (2014). Enhancer RNA facilitates NELF release from immediate early genes. Mol. Cell *56*, 29–42.

Skalska, L., Beltran-Nebot, M., Ule, J., and Jenner, R.G. (2017). Regulatory feedback from nascent RNA to chromatin and transcription. Nat. Rev. Mol. Cell Biol. *18*, 331–337.

Subramanian, A., Tamayo, P., Mootha, V.K., Mukherjee, S., Ebert, B.L., Gillette, M.A., Paulovich, A., Pomeroy, S.L., Golub, T.R., Lander, E.S., and Mesirov, J.P. (2005). Gene set enrichment analysis: a knowledge-based approach for interpreting genome-wide expression profiles. Proc. Natl. Acad. Sci. USA *102*, 15545–15550.

Sullivan, K.D., Galbraith, M.D., Andrysik, Z., and Espinosa, J.M. (2018). Mechanisms of transcriptional regulation by p53. Cell Death Differ. *25*, 133–143.

Tiedje, C., Lubas, M., Tehrani, M., Menon, M.B., Ronkina, N., Rousseau, S., Cohen, P., Kotlyarov, A., and Gaestel, M. (2015). p38MAPK/MK2-mediated phosphorylation of RBM7 regulates the human nuclear exosome targeting complex. RNA *21*, 262–278.

Van Herreweghe, E., Egloff, S., Goiffon, I., Jády, B.E., Froment, C., Monsarrat, B., and Kiss, T. (2007). Dynamic remodelling of human 7SK snRNP controls the nuclear level of active P-TEFb. EMBO J. *26*, 3570–3580.

Vermeulen, W., and Fousteri, M. (2013). Mammalian transcription-coupled excision repair. Cold Spring Harb. Perspect. Biol. *5*, a012625.

Williamson, L., Saponaro, M., Boeing, S., East, P., Mitter, R., Kantidakis, T., Kelly, G.P., Lobley, A., Walker, J., Spencer-Dene, B., et al. (2017). UV irradiation induces a non-coding RNA that functionally opposes the protein encoded by the same gene. Cell *168*, 843–855.e813.

Wilson, M.D., Harreman, M., and Svejstrup, J.Q. (2013). Ubiquitylation and degradation of elongating RNA polymerase II: the last resort. Biochim. Biophys. Acta *1829*, 151–157.

Wu, Z.H., Shi, Y., Tibbetts, R.S., and Miyamoto, S. (2006). Molecular linkage between the kinase ATM and NF-kappaB signaling in response to genotoxic stimuli. Science *311*, 1141–1146.

Xue, Y., Yang, Z., Chen, R., and Zhou, Q. (2010). A capping-independent function of MePCE in stabilizing 7SK snRNA and facilitating the assembly of 7SK snRNP. Nucleic Acids Res. *38*, 360–369.

Yang, Z., Zhu, Q., Luo, K., and Zhou, Q. (2001). The 7SK small nuclear RNA inhibits the CDK9/cyclin T1 kinase to control transcription. Nature *414*, 317–322.

Zhou, P., Qian, L., Kozopas, K.M., and Craig, R.W. (1997). Mcl-1, a Bcl-2 family member, delays the death of hematopoietic cells under a variety of apoptosis-inducing conditions. Blood *89*, 630–643.

Zhou, Q., Li, T., and Price, D.H. (2012). RNA polymerase II elongation control. Annu. Rev. Biochem. *81*, 119–143.

# STAR★METHODS

## KEY RESOURCES TABLE

| REAGENT or RESOURCE | SOURCE | IDENTIFIER |
|---|---|---|
| Antibodies | | |
| Mouse monoclonal anti-FLAG M2 | Sigma | Cat#F1804; RRID: AB_262044 |
| Mouse monoclonal anti-FLAG M2 | Sigma | Cat#F3165; RRID: AB_259529 |
| Rabbit monoclonal anti-RNA polymerase II RPB1 | Abcam | Cat#ab76123; RRID: AB_1310643 |
| Mouse monoclonal anti-RNA polymerase II CTD repeat YSPTSPS [8WG16] | Abcam | Cat#ab817; RRID: AB_306327 |
| Rabbit polyclonal anti-RNA polymerase II CTD repeat YSPTSPS (phospho S2) | Abcam | Cat#ab5095; RRID: AB_304749 |
| Goat polyclonal anti-HEXIM1 | Everest Biotech | Cat#EB06964; RRID: AB_2118260 |
| Rabbit polyclonal anti-RBM7 | Sigma | Cat#HPA013993; RRID: AB_1856137 |
| Rabbit polyclonal anti-RBM7 | Proteintech | Cat#21896-1-AP |
| Rabbit polyclonal anti-CDK9 | Santa Cruz Biotechnology | Cat#sc-484; RRID: AB_2275986 |
| Rabbit monoclonal anti-CDK9 | Cell Signaling Technology | Cat#2316; RRID: AB_2291505 |
| Rabbit polyclonal anti-LARP7 | Laboratory of Q. Zhou (UC Berkeley) (He et al., 2008) | N/A |
| Goat polyclonal anti-MePCE | Santa Cruz Biotechnology | Cat#sc-82542; RRID: AB_2266399 |
| Mouse monoclonal anti-hnRNP A1 | Abnova | Cat#MAB2492; RRID: AB_10553148 |
| Mouse monoclonal anti-γ-H2AX (phospho S140) | Abcam | Cat#ab22551; RRID: AB_447150 |
| Rabbit polyclonal anti-MTR4 | Abcam | Cat#ab70551; RRID: AB_1270701 |
| Mouse monoclonal anti-EXOSC3 | Santa Cruz Biotechnology | Cat#sc-166568; RRID: AB_2278187 |
| Rabbit polyclonal anti-Cyclin T1 | Santa Cruz Biotechnology | Cat#sc-10750; RRID: AB_2073888 |
| Rabbit polyclonal anti-Cleaved PARP | Cell Signaling Technology | Cat#9541; RRID: AB_331426 |
| Rabbit polyclonal anti-Cleaved Caspase-3 | Cell Signaling Technology | Cat#9661; RRID: AB_2341188 |
| Mouse monoclonal anti-GAPDH | Santa Cruz Biotechnology | Cat#sc-32233; RRID:AB_627679 |
| Mouse monoclonal anti-p53 | Santa Cruz Biotechnology | Cat#sc-126; RRID: AB_628082 |
| Rabbit monoclonal anti-phospho-p38$^{MAPK}$ | Cell Signaling Technology | Cat#4511; RRID: AB_2139682 |
| Normal mouse IgG | Santa Cruz Biotechnology | Cat#sc-2025; RRID: AB_737182 |
| Normal rabbit IgG | Santa Cruz Biotechnology | Cat#sc-2027; RRID: AB_737197 |
| Bacterial and Virus Strains | | |
| *E. coli* BL21 (DE3) | Merck | Cat#69450 |
| *E. coli* BL21 (DE3) Rosetta pLysS | Merck | Cat#70956 |
| Chemicals, Peptides, and Recombinant Proteins | | |
| Zeocin | InVivogen | Cat#ant-zn-1 |
| Hygromycin B Gold | InVivogen | Cat#ant-hg-5 |
| Blasticidin | InVivogen | Cat#ant-bl-1 |
| Phusion High-Fidelity DNA polymerase | New England Biolabs | Cat#M0530 |
| 4-Nitroquinoline N-oxide | Sigma | Cat#N8141 |
| Flavopiridol | Sigma | Cat#F3055 |
| p38$^{MAPK}$ inhibitor SB203580 | Selleckchem | Cat#S1076 |
| 4-Thiouridine | Carbosynth | Cat#NT06186 |
| Tetracycline hydrochloride | Sigma | Cat#T7660 |
| Doxycycline hyclate | Sigma | Cat#D9891 |
| EDTA-free Protease Inhibitor Cocktail | Sigma | Cat#11873580001 |
| SUPERase In RNase Inhibitor | Thermo Fisher Scientific | Cat#AM2694 |
| Random hexamers | Thermo Fisher Scientific | Cat#N8080127 |

(*Continued on next page*)

***Continued***

| REAGENT or RESOURCE | SOURCE | IDENTIFIER |
|---|---|---|
| RNase A | Thermo Fisher Scientific | Cat#12091021 |
| TRIzol LS reagent | Thermo Fisher Scientific | Cat#10296028 |
| TRI Reagent | Sigma | Cat#T9424 |
| FastStart Universal SYBR Green QPCR Master (Rox) | Sigma | Cat#4913914001 |
| goat anti-mouse conjugated with AlexaFluor 488 | Thermo Fisher Scientific | Cat#A11001 |
| NucBlue reagent | Thermo Fisher Scientific | Cat#R37606 |
| ProLong Gold Antifade Mountant | Thermo Fisher Scientific | Cat#P10144 |
| 3XFLAG peptide | ApexBio Technology | Cat#A6001 |
| EZ-Link Biotin-HPDP | Thermo Fisher Scientific | Cat#21341 |
| Lipofectamine RNAiMAX reagent | Thermo Fisher Scientific | Cat#13778-150 |
| X-tremeGENE transfection reagent | Sigma | Cat#XTG9-RO |
| ChIP Cross-link Gold reagent | Diagenode | Cat#C01019027 |
| MBP | Laboratory of M. Geyer (University of Bonn) | N/A |
| MBP-RBM7 | This paper | N/A |
| MBP-RBM7 mRNP1 | This paper | N/A |
| GST-HEXIM1 | Laboratory of M. Geyer (University of Bonn) | N/A |
| GST-MePCE (aa 400-689) | Laboratory of M. Geyer (University of Bonn) | N/A |
| GST-LARP7 | Laboratory of M. Geyer (University of Bonn) | N/A |
| His-CDK9/GST-CycT1 (aa 1-272) | Laboratory of M. Geyer (University of Bonn) | N/A |
| Critical Commercial Assays | | |
| Flp-In T-REx Core Kit | Thermo Fisher Scientific | Cat#K650001 |
| Rapid DNA Ligation Kit | Thermo Fisher Scientific | Cat#K1423 |
| Dynabeads Protein G | Thermo Fisher Scientific | Cat#10004D |
| M-MLV reverse transcriptase | Thermo Fisher Scientific | Cat#28025-013 |
| SuperScript III reverse transcriptase | Thermo Fisher Scientific | Cat#18080044 |
| AccuPrime SuperMix I | Thermo Fisher Scientific | Cat#12342010 |
| Turbo DNA-Free kit | Thermo Fisher Scientific | Cat#AM1907 |
| Anti-FLAG M2 affinity gel | Sigma | Cat#A2220 |
| μMACS Streptavidin Kit | Miltenyi | Cat#130-074-101 |
| TruSeq Stranded mRNA LT Sample Prep Kit | Illumina | Cat#RS-122-2101 |
| CellTox Green Cytotoxicity Assay | Promega | Cat#G8741 |
| RealTime-Glo Annexin V Apoptosis and Necrosis Assay | Promega | Cat#JA1011 |
| alamarBlue Cell Viability Assay | Thermo Fisher Scientific | Cat#DAL 1025 |
| MycoAlert mycoplasma detection kit | Lonza | Cat#LT07-118 |
| MBPTrap HP – 5 mL prepacked column | GE Healthcare | Cat#28918780 |
| GSTrap FF – 5 mL prepacked column | GE Healthcare | Cat#17513001 |
| HiLoad 16/600 Superdex200 pg | GE Healthcare | Cat#GE28-9893-35 |
| HiLoad 16/600 Superdex75 pg | GE Healthcare | Cat#GE28-9893-33 |
| Deposited Data | | |
| RBM7 iCLIP | EMBL-EBI ArrayExpress Archive | E-MTAB-6475 |
| 4sU-seq | NCBI Gene Expression Omnibus | GEO: GSE110272 |

(*Continued on next page*)

| *Continued* | | |
|---|---|---|
| REAGENT or RESOURCE | SOURCE | IDENTIFIER |
| Human reference genome UCSC assembly hg19 (GRCh37) | Genome Reference Consortium | http://hgdownload.cse.ucsc.edu/goldenPath/hg19/ |
| Human reference genome UCSC assembly hg 38 (GRCh38) | Genome Reference Consortium | http://hgdownload.cse.ucsc.edu/goldenPath/hg38/ |
| Experimental Models: Cell Lines | | |
| HEK293 Flp-In T-REx | Thermo Fisher Scientific | Cat#R78007 |
| HEK293 Flp-In T-REx 3XFLAG | This paper | N/A |
| HEK293 Flp-In T-REx F-RBM7 | Laboratory of J. Ule (The Francis Crick Institute) | N/A |
| HEK293 Flp-In T-REx F-RBM7 mRNP1 | This paper | N/A |
| HEK293 Flp-In T-REx F-LARP7 | This paper | N/A |
| HEK293 Flp-In T-REx F-CDK9 | This paper | N/A |
| HEK293 Flp-In T-REx F-HEXIM1 | This paper | N/A |
| HEK293 Flp-In T-REx F-MTR4 | Laboratory of M. Nagahama (Meiji Pharmaceutical University) (Hiraishi et al., 2015) | N/A |
| HeLa Flp-In | Laboratory of E. Bertrand (University of Montpellier) | N/A |
| HeLa Flp-In F-RBM7 | This paper | N/A |
| HeLa Flp-In F-RBM7 mRNP1 | This paper | N/A |
| HeLa | ATCC | Cat#CCL2; RRID: CVCL_0045 |
| RPE-1 | ATCC | Cat#CRL-4000; RRID: CVCL_4388 |
| HFF-1 | ATCC | Cat#SCRC-1041; RRID: CVCL_3285 |
| HCT116 *TP53*$^{+/+}$ and HCT116 *TP53*$^{-/-}$ | Laboratory of J.M. Espinosa (University of Colorado) | N/A |
| Oligonucleotides | | |
| pcDNA5/FRT/TO/3XFLAG-RBM7 mRNP1 mutagenesis primers: TGCGGCTGTGAATTTCAAACATGAAGTG GCCTGCGCTGGTTTACCATCCTTATCTTTTG | Integrated DNA Technologies | N/A |
| 7SK antisense DNA: CCTTGAGAGCTTGTTTGGAGG | Integrated DNA Technologies | N/A |
| RBM7 siRNA #1: GCGUAAAGUCAGAAUGAAUTT | Integrated DNA Technologies | N/A |
| RBM7 siRNA #2: GGAUAAAGGCAUUGCUUAATT | Integrated DNA Technologies | N/A |
| hMTR4 siRNA: CAAUUAAGGCUCUGAGUAATT | Integrated DNA Technologies | N/A |
| Control siRNA | QIAGEN | Cat#SI03650318 |
| Primers for RIP-qPCR assays, see Table S5A | Integrated DNA Technologies | N/A |
| Primers for RT-qPCR assays, see Table S5B | Integrated DNA Technologies | N/A |
| Primers for ChIP-qPCR assays, see Table S5C | Integrated DNA Technologies | N/A |
| Recombinant DNA | | |
| pcDNA5/FRT/TO/3XFLAG | Laboratory of J. Ule (The Francis Crick Institute) | N/A |
| pcDNA5/FRT/TO/3XFLAG-RBM7 | Laboratory of J. Ule (The Francis Crick Institute) | N/A |
| pcDNA5/FRT/TO/3XFLAG-RBM7 mRNP1 | This paper | N/A |
| pcDNA5/FRT/TO/3XFLAG-LARP7 | This paper | N/A |
| pcDNA5/FRT/TO/3XFLAG-CDK9 | This paper | N/A |
| pcDNA5/FRT/TO/3XFLAG-HEXIM1 | This paper | N/A |
| pEF.YN.CTD | Laboratory of B.M. Peterlin (UCSF) (Fujinaga et al., 2015) | N/A |
| pEF-YC.P-TEFb | Laboratory of B.M. Peterlin (UCSF) (Fujinaga et al., 2015) | N/A |

(*Continued on next page*)

*Continued*

| REAGENT or RESOURCE | SOURCE | IDENTIFIER |
|---|---|---|
| pET28a/MBP | Laboratory of M. Geyer (University of Bonn) | N/A |
| pET28a/MBP-RBM7 | This paper | N/A |
| pET28a/MBP-RBM7 mRNP1 | This paper | N/A |
| pGEX4T1/HEXIM1 | Laboratory of M. Geyer (University of Bonn) | N/A |
| pGEX4T1/MePCE (aa 400-689) | Laboratory of M. Geyer (University of Bonn) | N/A |
| pGEX4T1/LARP7 | Laboratory of M. Geyer (University of Bonn) | N/A |
| pGEX4T1/CycT1 (aa 1-272) | Laboratory of M. Geyer (University of Bonn) | N/A |
| pACEBac1/CDK9 | Laboratory of M. Geyer (University of Bonn) | N/A |
| Software and Algorithms | | |
| iCount | Laboratory of T. Curk (University of Ljubljana) | http://icount.biolab.si https://github.com/tomazc/iCount |
| STAR RNA aligner | Dobin et al., 2013 | RRID: SCR_015899; https://github.com/alexdobin/STAR |
| FastQC | Babraham Bioinformatics | http://www.bioinformatics.babraham.ac.uk/projects/fastqc/ |
| Cutadapt | Martin, 2011 | https://cutadapt.readthedocs.io/en/stable/guide.html |
| ContextMap 2 | Bonfert et al., 2015 | https://www.bio.ifi.lmu.de/software/contextmap/index.html |
| featureCounts | Liao et al., 2014 | http://bioinf.wehi.edu.au/featureCounts/ |
| edgeR | Robinson et al., 2010 | https://bioconductor.org/packages/release/bioc/html/edgeR.html |
| Ingenuity Pathway Analysis v01-08 - IPA Upstream Regulator Analysis | Ingenuity Pathway Analysis | http://pages.ingenuity.com/rs/ingenuity/images/0812%20upstream_regulator_analysis_whitepaper.pdf |
| Ingenuity Pathway Analysis v01-08 - IPA Downstream Effects analytic | Ingenuity Pathway Analysis | http://pages.ingenuity.com/rs/ingenuity/images/0812%20downstream_effects_analysis_whitepaper.pdf |
| RcisTarget v1.0.2 | Bioconductor (Aibar et al., 2017) | http://bioconductor.org/packages/release/bioc/html/RcisTarget.html |
| Molecular Signatures Database v6.0 | GSEA - Broad Institute (Subramanian et al., 2005) | http://software.broadinstitute.org/gsea/msigdb/index.jsp |
| AxioVision v4.3 Microscopy Software | Zeiss | N/A |
| MetaMorph Microscopy Automation and Image Analysis Software | Molecular Devices | N/A |
| MxPro QPCR Software v4.10 | Stratagene | N/A |

## CONTACT FOR REAGENT AND RESOURCE SHARING

Further information and requests for reagents may be directed to and will be fulfilled by the Lead Contact, Matjaž Barborič (matjaz.barboric@helsinki.fi).

## EXPERIMENTAL MODEL AND SUBJECT DETAILS

HEK293 Flp-In T-REx (Thermo Fisher Scientific), HeLa (ATCC), HeLa Flp-In and human foreskin fibroblast (HFF-1, ATCC) cell lines were grown in Dulbecco‘s Modified Eagle‘s Medium (D-MEM; Sigma, D5796) supplemented with 10% fetal bovine serum (FBS) and 100 U/ml penicillin/streptomycin. The parental HEK293 Flp-In T-REx and HeLa Flp-In cell lines were grown with 50 μg/ml of

Zeocin (InVivogen). HEK293 Flp-In T-REx F-hMTR4 and HeLa Flp-In cell lines were described previously (Hallais et al., 2013; Hiraishi et al., 2015). HEK293 Flp-In T-REx and HeLa Flp-In cell lines expressing 3XFLAG peptide or 3XFLAG epitope-tagged proteins were generated using Flp-In T-REx Core Kit (Thermo Fisher Scientific) according to the manufacturer's instructions and grown with 100 μg/ml of Hygromycin B Gold (InVivogen) and 3 μg/ml of Blasticidin (InVivogen). Retinal pigment epithelium (RPE-1) cell line (ATCC) was grown in D-MEM/F12 (Sigma, D8437) supplemented with 10% FBS and 100 U/ml penicillin/streptomycin. HCT116 *TP53*$^{+/+}$ and HCT116 *TP53*$^{-/-}$ cell lines were grown in McCoy's 5A Medium (Sigma, M9309) supplemented with 10% FBS and 100 U/ml penicillin/streptomycin. All cell lines were maintained at 37°C with 5% $CO_2$. Cell lines were confirmed to be mycoplasma-free using MycoAlert mycoplasma detection kit (Lonza). Cell lines were not authenticated by us, but retrieved from trusted sources as listed in the Key Resources Table.

## METHOD DETAILS

### Plasmid DNAs and Mutagenesis

To generate plasmid DNAs encoding 3XFLAG epitope-tagged HEXIM1, LARP7, CDK9 and RBM7, the cDNAs were amplified using Phusion High-Fidelity DNA polymerase (NEB) with primers carrying the appropriate restriction enzymes sites and cloned using Rapid DNA Ligation Kit (Thermo Fisher Scientific) into pcDNA5/FRT/TO vector, which was modified to encode 3XFLAG peptide upstream of the multiple cloning site (a gift from Dr. Ule). Q5® Site-Directed Mutagenesis Kit (NEB) was used for generating pcDNA5/FRT/TO/3XFLAG-RBM7 mRNP1 plasmid encoding the mutant mRNP1 F-RBM7 protein. RBM7 mutagenesis primer sequences and plasmids used in this study are listed in the Key Resources Table. To generate plasmid DNAs encoding the MBP-tagged RBM7 and mRNP1 RBM7 the cDNAs were cloned into a modified pET28a vector containing an N-terminal MBP sequence followed by a TEV protease cleavage site. To generate baculovirus transfer vector encoding His-tagged CDK9, its cDNA was cloned into pACEBac1 with a sequence encoding OctaHis-tag. All other cDNAs encoding full-length HEXIM1 and LARP7 as well as MePCE (aa 400-689) and CycT1 (aa 1-272) were cloned into a pGEX4T1 vector modified to contain a TEV protease cleavage site between the GST tag and the protein.

### Chemicals and Treatments

4-Nitroquinoline N-oxide (4-NQO; Sigma) was diluted in DMSO to a final concentration of 50 mM, aliquoted, sealed, and stored at −80°C. UV irradiation was performed in Crosslinker CL-1000 using 254 nm wavelength lamp with dose 40-60 J/m$^2$. Flavopiridol (Sigma) and 4-Thiouridine (4sU; Carbosynth) were diluted in DMSO to a final concentration of 1 mM and stored at −20°C. Tetracycline hydrochloride (Sigma) was diluted in water to a final concentration of 1 mg/ml and stored at −20°C. SB203580 (Selleckchem) was diluted in DMSO to a final concentration of 50 mM and stored in −20°C.

### RBM7 iCLIP Assay

HEK293 Flp-In T-REx F-RBM7 cells were treated for 24 h with 2 μg/ml of doxycycline (Sigma) to express F-RBM7, exposed for 2 h to DMSO or 5 mM of 4-NQO, washed once with PBS and UV-cross-linked at 0,15 mJ/cm$^2$ with 254 nm wavelength. Immunoprecipitation of RNA-protein complexes, retrieval of protein-bound RNAs and preparation of cDNA libraries were conducted as reported previously (Huppertz et al., 2014). In brief, lysates were generated from the crosslinked cells and treated with Turbo DNase (Thermo Fisher Scientific) and RNase I (1:100 or 1:500; Ambion) for 3 min at 37°C to digest the genomic DNA and trim the RNA to short fragments of an optimal size range. RNA-protein complexes were immunoprecipitated using anti-FLAG M2 (Sigma, F1804) and Protein G Dynabeads (Thermo Fisher Scientific). Following stringent high salt washes, the immunoprecipitated RNA was 5′ end-labeled using radioactive $^{32}$P isotopes followed by on-bead-ligation of pre-adenylated adaptors to the 3′ end. The immunoprecipitated complexes were separated with SDS-PAGE and transferred to a BA-85 nitrocellulose membrane (Protran). RNA was recovered by digesting proteins using proteinase K and then reverse transcribed into cDNA using SuperScript III reverse transcriptase (Thermo Fisher Sceintific). The reverse transcription primers contained barcode sequences to enable multiplexing and a BamHI restriction enzyme site. The cDNA was size selected, circularized to add the adaptor to the 5′ end, digested at the internal BamHI site, and then PCR amplified using AccuPrime SuperMix I (Thermo Fisher Scientific). The final PCR libraries were purified on PCR purification columns (Fermentas) to remove residual PCR reagents and submitted for sequencing. iCLIP libraries were multiplexed and sequenced on Illumina HiSeq2 machine in a single-end manner with a read length of 50nt. Sequenced reads were de-multiplexed into individual libraries based on their barcodes and collapsed to remove PCR duplicates. The barcode sequences and adaptors were trimmed from the 5′ and 3′ ends, respectively. Following mapping to the human genome (hg19) with STAR aligner (Dobin et al., 2013) as part of the iCount package (http://icount.biolab.si/), cross-linked nucleotides were defined as the nucleotide upstream of mapped iCLIP cDNA tags as reported previously (Konig et al., 2010). Replicate iCLIP experiments were performed, cross-linking positions compared between samples, and the replicates were subsequently combined into groups for final analysis using the iCount package.

### Quantitative RNA Immunoprecipitation Assay

HEK293 Flp-In T-REx cells grown on 15 cm plates were treated for 16 h with 1 μg/ml of tetracycline to express the 3XFLAG epitope-tagged proteins. The cells at approximately 90% confluency were then exposed to DMSO or 10 μM of 4-NQO for 2 h unless noted

otherwise. The cells were resuspended in Falcon tubes with 10 mL of ice-cold PBS and cross-linked with 1% formaldehyde at room temperature for 10 min, which was stopped with 250 mM of glycine for 5 min. The cells were then washed twice with ice-cold PBS, resuspended in buffer A (5 mM PIPES, 85 mM KCL, 0.5% NP-40, pH 7.9), and incubated 10 min on ice for nuclear extraction. Nuclear pellets obtained by centrifugation were lysed in 1 mL of RIPA buffer (50 mM Tris pH 8.0, 150 mM NaCL, 5 mM EDTA pH 8.0, 0.5% sodium deoxycholate, 1% NP-40, 0.1% SDS) in the presence of EDTA-free Protease Inhibitor Cocktail (Sigma) and SUPERase In RNase Inhibitor (Thermo Fisher Scientific). The lysates were then sonicated during one round of 35 cycles of 30 s ON/30 s OFF at 4°C with the Bioruptor Plus sonication device (Diagenode, B01020001) combined with the Bioruptor Water cooler (Cat. No. BioAcc-cool) & Single Cycle Valve (Cat. No. VB-100-0001) at high power setting (position H) using 1.5 mL TPX microtubes (Diagenode, M-50001). After centrifugation at 13 000 g for 15 min, 5% of supernatant was stored at −80°C for determining RNA input. We used one third of the rest of the lysate per IP, with the exception of F-RBM7 RIP-qPCRs, where we used one sixth per IP. The sonicated lysates were then supplemented with additional RIPA buffer to the total volume of 900 μL and incubated overnight at 4°C with 15 μL of antibody-coupled Protein G Dynabeads (Thermo Fisher Scientific). Before adding the supernatant, the beads were pre-blocked with bovine serum albumin and salmon sperm DNA overnight at a final concentration of 0.2 μg/μl, pre-incubated in 500 μL of RIPA buffer for 4 h with the antibody and collected by magnetic stand to remove the unbound antibody. We used 3 μg of normal mouse IgG (Santa Cruz Biotechnology), 3 μg of anti-FLAG M2 (Sigma, F1804), or 3 μg of anti-HEXIM1 (Everest Biotech) antibody. The beads were then washed with RIPA high salt buffer (20 mM Tris pH 8.0, 500 mM NaCL, 2 mM EDTA pH 8.0, 1% Triton-X, 0.1% SDS), RIPA low salt buffer (20 mM Tris pH 8.0, 150 mM NaCL, 2 mM EDTA pH 8.0, 1% Triton-X, 0.1% SDS), LiCl wash buffer (250 mM LiCl, 1% NP-40, 1% sodium deoxycholate) and TE buffer (10 mM Tris, 1 mM EDTA, pH 8.0). RNA-protein complexes were eluted at room temperature with elution buffer (1% SDS, 100 mM $NaHCO_3$). After reverse the cross-linking, RNAs from eluates and inputs were isolated using TRIzol LS reagent (Thermo Fisher Scientific) according to the manufacturer's protocol. RNA samples were DNase-treated with the Turbo DNA-Free kit (Thermo Fisher Scientific), reverse transcribed with SuperScript III reverse transcriptase (Thermo Fisher Sceintific) and random hexamers (Thermo Fisher Scientific), and amplified using FastStart Universal SYBR Green QPCR Master (Rox) (Sigma), RNA-specific primer pair, and Stratagene Mx3005 qPCR machine. Primers were from Integrated DNA Technologies and designed using PrimerQuest Tool. Values were normalized to their levels in RNA inputs and calculated using the MxPro QPCR Software. Results from at least three independent experiments are presented as the mean ± SEM. Sequences of the primers used in RIP-qPCR assays are listed in Table S5A.

## Co-immunoprecipitation and Western Blotting Assays

HEK293 Flp-In T-REx cells grown on 10 cm plates were treated for 16 h with 1 μg/ml of tetracycline to express the 3XFLAG epitope-tagged proteins. The cells were then exposed to DMSO or 10 μM of 4-NQO for 2 h unless noted otherwise. Whole cell extracts (WCE) were prepared by lysing the cell pellets on ice for 15 min with buffer C (20 mM Tris-HCl, 0.5% NP-40, 150 mM NaCl, 1.5 mM $MgCl_2$, 10 mM KCl, 10% Glycerol, 0.5 mM EDTA, pH 7.9) in the presence of EDTA-free Protease Inhibitor Cocktail (Sigma), followed by optional 10 s sonication with the Misonix XL-2000 Ultrasonic Liquid Processor using the P-1 Microprobe 3.2 mm tip, power setting 3, and centrifugation of lysates at 20 000 g for 15 min. For FLAG immunoprecipitation, WCE were incubated at 4°C for 4 h with buffer C-equilibrated anti-FLAG M2 affinity gel (Sigma), immuno-complexes were washed three times with buffer C, eluted in SDS running buffer in the presence of dithiothreitol (Sigma) for 5 min at 95°C and resolved using 12% SDS-PAGE. For total Pol II co-immunoprecipitation (coIP), 1 μg of the anti-RNA polymerase II CTD repeat YSPTSPS [8WG16] antibody (Abcam) was immobilized on Protein G Dynabeads (Thermo Fisher Scientific) according to manufacturer's instructions. WCE were prepared using ELB buffer (50 mM HEPES-KOH pH 7.9, 0.1% Triton X-100, 5 mM dithiothreitol, 5 mM EDTA, 150 mM NaCl), incubated with the ELB buffer-equilibrated beads for 16 h at 4°C, and the beads were washed three times ELB buffer. For endogenous HEXIM1 coIP, 2 μg of the anti-HEXIM1 antibody (Everest Biotech) was immobilized on Protein G Dynabeads (Thermo Fisher Scientific) according to manufacturer's instructions. In experiments with the $p38^{MAPK}$ inhibitor SB203580, HeLa Flp-In cells were pre-treated with 10 μM of the inhibitor for 1 h, followed by 1 μM of 4-NQO for 30 min. In the case of HEXIM1 coIP using HEK293 Flp-In T-REx F-RBM7 and HEK293 Flp-In T-REx F-RBM7 mRNP1 cell lines, the cells were treated for 24 h with 1 μg/ml of tetracycline to express the 3XFLAG epitope-tagged proteins. Upon cell lysis in buffer C, WCE were then added to the buffer C-equilibrated beads and incubated for 16 h at 4°C, and the beads were washed three times with buffer C. 50% of the beads was used to determine the levels of HEXIM1, CDK9 and F-RBM7 by western blotting. To determine the levels of 7SK in anti-HEXIM1 coIP, 500 μL of TRI reagent (Sigma) was added to the remaining 50% of the beads for RNA extraction. RNA samples were DNase-treated with the Turbo DNA-Free kit (Ambion), reverse transcribed with SuperScript III reverse transcriptase (Thermo Fisher Sceintific) and random hexamers (Thermo Fisher Scientific), and quantified with the Stratagene Mx3005 qPCR machine as described above using 5S rRNA as a normalizing control. Results from independent experiments are presented as the mean ± SEM. For western blotting, the following antibodies were used according to manufacturers' instructions: anti-FLAG (Sigma); anti-RBM7 (Sigma, Proteintech); anti-HEXIM1 (Everest Biotech); anti-CDK9 (Santa Cruz Biotechnology, Cell Signaling Technology); anti-LARP7 (a gift from Dr. Qiang Zhou); anti-MePCE (Santa Cruz Biotechnology); anti-RNA polymerase II CTD repeat YSPTSPS (phospho S2) antibody (Abcam); anti-hnRNP A1 (Abnova); anti-γ-H2AX (Abcam); anti-MTR4 (Abcam); anti-EXOSC3 (Santa Cruz Biotechnology); anti-Cyclin T1 (Santa Cruz Biotechnology), anti-Cleaved PARP (Cell Signaling Technology); anti-Cleaved Caspase-3 (Cell Signaling Technology). Manufacturers provide validation for all antibodies.

## Glycerol Gradient Sedimentation Analysis

HeLa Flp-In cells were treated with DMSO or 10 μM of 4-NQO for 2 h, lysed on ice for 15 min in 0.6 mL of lysis buffer B (20 mM HEPES, 0.3 M KCl, 0.2 mM EDTA, 0.1% NP-40, 0.1% protease inhibitor, pH 7.9). Cellular extracts were then subjected to ultracentrifugation in a SW41 Ti rotor (Beckman) at 38 000 rpm for 16 h in a 10 mL glycerol gradient solution (10%–30%) containing buffer B. Ten fractions were collected, and the proteins in each fraction were precipitated by 70% trichloroacetic acid and analyzed by western blotting.

## Immunofluorescence Microscopy

HeLa cells were grown on polylysine coated coverslips, treated with DMSO or 10 μM of 4-NQO for 1 h, washed twice in PBS, and incubated in cytoskeletal buffer (CSK; 10 mM Pipes pH 6.8, 300 mM sucrose, 100 mM NaCl, 3 mM $MgCl_2$, 1 mM EGTA) for 10 min. Cells were then fixed in 4% formaldehyde in CSK buffer for 1 h on ice and blocked with TBS-I (10 mM Tris pH 7.7, 150 mM NaCl, 3 mM KCl, 1.5 mM $MgCl_2$, 0.05% Tween 20, 0.1% BSA, 0.2% glycine) for at least 1 h on ice. Primary antibody staining using anti-γH2AX (Abcam; 1:100 dilution) was done overnight at 4°C, which was followed by two washes with ice cold CSK buffer. Secondary antibody staining using goat anti-mouse conjugated with AlexaFluor 488 (Thermo Fisher Scientific; 1:1000 dilution) was performed for 4 h at 4°C. Cells were then washed twice with ice cold CSK buffer, incubated with NucBlue reagent (Thermo Fisher Scientific) for 30 min at room temperature and washed three times with ice cold CSK buffer. Coverslips were mounted with ProLong Gold Antifade Mountant (Thermo Fisher Scientific). Images were acquired by AxioLab microscope equipped with AxioVision 4.3 Microscopy Software (Zeiss), and analyzed using CorelDRAW Graphic Suite 2017. Representative images show cells treated with DMSO or 250 nM of 4-NQO for 1 h. The number of cells with at least one nuclear γ-H2AX foci from two independent experiments was counted, plotted as percentage of the total number of cells in the field, and presented as the mean ± SEM. For each independent treatment, cells were counted from at least ten fields containing at least ten cells per field.

## Bimolecular Fluorescence Complementation Assay

HeLa Flp-In cells grown on 6-well plates were co-transfected with 0.2 μg of pEF.YN-CTD and 2 μg of pEF.YC-P-TEFb plasmids using X-tremeGENE transfection reagent (Sigma). Twenty-four hours after transfection, the cells were seeded into 6-8 wells of a 24-well plate and grown an additional 24 to 48 h. The cells were then left untreated or treated with DMSO, with 2, 5, and 10 μM of 4-NQO for 0.5, 1, and 4 h, and with 5 μM of SAHA or JQ1 for 1 h. Fluorescence signals were detected using Olympus IX70 fluorescent microscope. The fluorescence images were analyzed using MetaMorph Microscopy Automation and Image Analysis Software (Molecular Devices). YFP positive cells were counted manually and averaged from three randomly chosen fields of each sample.

## P-TEFb Release Assay

P-TEFb release assay was performed as reported previously (Calo et al., 2015) with the following modifications. WCE from one confluent 15 cm plate of HeLa cells was prepared by lysing the cell pellets on ice for 30 min with 1.2 mL of buffer C in the presence of EDTA-free Protease Inhibitor Cocktail (Sigma). To immobilize 7SK snRNP, 1 mL of WCE was incubated at 4°C for 4 h with 75 μL of Protein G Dynabeads (Thermo Fisher Scientific) that were pre-bound with 5 μg of anti-HEXIM1 antibody (Everest Biotech). For the control, the remaining 200 μL of WCE was incubated with 15 μL of Protein G Dynabeads (Thermo Fisher Scientific) that were pre-bound with 1 μg of normal IgG antibody. Immuno-purified F-RBM7 proteins were incubated with the equal amounts of immobilized 7SK snRNP for 2 h on ice, which was followed by the collection of samples for western blotting analysis from 50% of the beads. To determine the levels of 7SK that remained bound to HEXIM1, 500 μL of TRI reagent (Sigma) was added to the remaining 50% of the beads for RNA extraction. RNA samples were DNase-treated with the Turbo DNA-Free kit (Thermo Fisher Sceintific), reverse transcribed with SuperScript III reverse transcriptase (Thermo Fisher Sceintific) and random hexamers (Thermo Fisher Scientific), and quantified with the Stratagene Mx3005 qPCR machine as described above using 5S rRNA as a normalizing control. Results from independent experiments are presented as the mean ± SEM. For purification of the 3XFLAG epitope-tagged RBM7 proteins, the corresponding HEK293 Flp-In T-REx F-RBM7 cells grown on three 15 cm plates were treated for 16 h with 1 μg/ml of tetracycline to express the F-RBM7 proteins. The cells were lysed in buffer C. WCE were then incubated with 30 μL of buffer C-equilibrated anti-FLAG M2 affinity gel (Sigma) for 16 h in the presence of EDTA-free Protease Inhibitor Cocktail (Sigma) and RNase A (Thermo Fisher Scientific). After incubation, samples were washed three times with buffer C, where the first two washes contained 600 mM of NaCl. F-RBM7 proteins were eluted with 300 μg/ml of 3XFLAG peptide (ApexBio Technology) in 50 μL of buffer C, and 10 μL (+) or 20 μL (++) of the eluates were used per each reaction. To examine their purity, 10 μL eluate samples were subjected to SDS-PAGE and western blotting using anti-hMTR4, anti-EXOSC3, and anti-CDK9 antibodies.

## Expression of Recombinant Proteins

MBP-RBM7 and mRNP1 MBP-RBM7 proteins were expressed in *E. coli* BL21 (DE3) Rosetta pLysS cells grown in lysogeny broth (LB) medium supplemented with kanamycin (50 μg/ml) and chloramphenicol (34 μg/ml). GST-HEXIM1, GST-MePCE (aa 400-689), GST-CycT1 (aa 1-272) and GST-LARP7 were expressed in *E. coli* BL21 (DE3) cells grown in LB medium containing ampicillin (100 μg/ml). All proteins were expressed for 16 h at 18°C after induction of expression with 0.5 mM Isopropyl-β-D-thiogalactopyranosid (IPTG) at an OD 600nm of 0.8. His-CDK9 was expressed in *Sf9* insect cells for three days at 27°C by infecting the cells at a density of $1.5x10^6$ cells/ml with the baculovirus of a multiplicity of infection > 1.

### Purification of Recombinant Proteins

*E. coli* pellets were resuspended in lysis buffer (50 mM HEPES pH7.6, 150 mM NaCl, 5 mM β-Mercaptoethanol) and lysed by sonication. Afterward, lysate was centrifuged to remove cell debris, filtered through a 0.45 μm filter and subsequently used for purification. Proteins were purified using an ÄKTAPrime FPLC system (GE Healthcare) by affinity chromatography using GSTrap FF or MBPTrap HP columns (GE Healthcare), respectively. Proteins were further purified by size exclusion chromatography (SEC). For affinity chromatography, columns were equilibrated in lysis buffer prior to sample application. After sample application, columns were washed excessively with lysis buffer containing 1 M NaCl. The proteins were eluted with lysis buffer containing 10 mM GSH or 10 mM maltose, respectively. To remove residual contaminations, the proteins were subjected to size exclusion chromatography at a Superdex200 pg (GE Healthcare) or Superdex75 pg (GE Healthcare) and eluted with one column volume of SEC buffer (20 mM HEPES, 150 mM NaCl, 1 mM TCEP). Fractions were analyzed by Coomassie staining of SDS-PAGE and those containing the protein of interest were pooled and concentrated by Ultrafiltration (Millipore). The heterodimeric P-TEFb complex was reconstituted by the addition of GST-CycT1 (aa 1-272) to the lysed His-CDK9-expressing Sf21 cells. The complex was purified by immobilized metal affinity chromatography using pre-packed Ni-NTA resin (GE Healthcare). After washing in a buffer containing 20 mM imidazol, the complex was eluted from Ni-columns by an imidazol gradient (final imidazol concentration 400 mM). Fractions containing P-TEFb were identified by Coomassie staining of SDS-PAGE and pooled for further purification by SEC on a Superdex200 pg column (GE healthcare).

### MBP-pull down Assay

For interaction of RBM7 with the components of the 7SK snRNP, 10 μg of MBP or MBP-RBM7 were incubated with 25 μg of prey protein in 100 μL binding buffer (20 mM HEPES pH7.6, 150 mM NaCl, 1 mM TCEP), followed by addition of 15 μL of a 50% slurry solution of Amylose beads and incubation for 2 h at 4°C. Afterward, beads were collected by centrifugation and washed three times with 500 μL of binding buffer. After the final washing step, excess buffer was removed and protein eluted from the beads with binding buffer containing 30 mM maltose. The eluate was then transferred to a fresh tube containing SDS sample buffer and boiled for 5 min.

### 4sU-sequencing Assay

Metabolic labeling and isolation of newly transcribed RNA was performed as reported previously (Rädle et al., 2013). In brief, 80% confluent HeLa Flp-In cells were mock-treated or treated with DMSO, 5 μM of 4-NQO, or 5 μM of 4-NQO and 250 nM of flavopiridol for 1 or 2 h before lysis. Thirty minutes prior to lysis, the cells were labeled with 4sU at a final cell culture medium concentration of 100 μM. RNA was extracted with TRI reagent (Sigma) and 150 μg of total RNA was used for biotinylation with EZ-Link Biotin-HPDP (Thermo Fisher Scientific) for 90 min at room temperature. Second round of RNA extraction was performed with chloroform-isopropanol. μMACS Streptavidin Kit (Miltenyi) was used for separation of labeled RNA, which was followed by elution with DTT and RNA extraction with isopropanol. Libraries from two biological replicates were prepared and sequenced by Beijing Genomics.

### Analysis of 4sU-sequencing Datasets

Sequencing quality was assessed with FastQC and sequencing adapters were trimmed using Cutadapt (Martin, 2011). Reads were mapped against the human reference genome (GRCh38/hg38) and rRNA sequences using ContextMap 2 (Bonfert et al., 2015). Read counts for mRNAs, lincRNAs, uaRNAs and eRNAs were calculated using featureCounts (Liao et al., 2014). mRNA and lincRNA annotation were taken from Gencode version 25 and eRNA annotations were taken from the study by Lubas et al. (2015). eRNAs within 5 kb of an annotated gene (according to Gencode) were excluded. uaRNAs were defined as the window from −3kb to the transcription start sites of mRNAs and lincRNAs on the opposite DNA strand. If according to the Gencode annotation another gene was present within 10 kb upstream of the uaRNA-associated gene, we excluded those uaRNAs from our analysis. Expression of mRNAs, lincRNAs, uaRNAs and eRNAs was quantified in terms of fragments per kilobase of exons per million mapped reads (FPKM) and averaged between replicates. Differential gene expression analysis to determine fold-changes in gene expression and significance of changes was performed using edgeR (Robinson et al., 2010). Here, only RNAs with an average read count $\geq$ 1 in the respective samples were included. EdgeR models read counts using the negative binomial distributions and uses the quantile-adjusted conditional maximum likelihood method to estimate log fold-changes and p values for differential gene expression. p values obtained from edgeR were corrected by multiple testing using the method by Benjamini and Hochberg (1995) for adjusting the false discovery rate (FDR) and a p value cutoff of 0.01 was applied. For correlation analysis, Spearman rank correlation was used. Gene lengths were obtained from Gencode annotations. Table S2E summarizes the number of mRNAs, lincRNAs, uaRNAs and eRNAs which were differentially expressed upon 1 and 2 h of 4-NQO treatment. A transcript was considered consistently regulated if (i) the FDR-adjusted p value was $\leq$ 0.01 in at least one of the two experimental conditions, (ii) it was regulated in the same way (either up or down) in both experimental conditions and (iii) it was not differentially expressed in the DMSO control condition.

### Ingenuity Pathway Analysis and Molecular Signature Database Analysis

The set of protein-coding genes that are differentially expressed upon 1 and 2 h of 4-NQO exposure and the 4FP gene set were subjected to IPA Upstream Regulator Analysis, which identifies upstream regulators that can explain the observed gene expression changes in a user's dataset. The 4FP gene set was also subjected to IPA Downstream Effects analytic, which identifies biological functions that are expected to be increased or decreased given the observed gene expression changes in a user's dataset. Detailed explanation of these

analyses is provided by IPA at http://pages.ingenuity.com/rs/ingenuity/images/0812%20upstream_regulator_analysis_whitepaper.pdf; and http://pages.ingenuity.com/rs/ingenuity/images/0812%20downstream_effects_analysis_whitepaper.pdf. Overlaps between the 4FP gene set and gene sets of the Molecular Signatures Database (MSigDB) collection v6.0 were computed using the on-line tool at http://software.broadinstitute.org/gsea/msigdb/index.jsp. Gene set enrichment analysis, GSEA software, and MSigDB were described previously (Subramanian et al., 2005).

### Transcription Factor Binding Motifs Analysis

Transcription factor binding motifs analysis was performed with RcisTarget version 1.0.2 (Aibar et al., 2017) using the 4FP gene set and the gene sequences within 5kb window around the transcription start sites (motif collection version mc9nr, regions selected based on conservation in 7 species). Transcription factors and genes highly ranked to the given motif were annotated to each motif with the normalized enrichment score (NES) of at least 3.

### p53 Target Gene Enrichment Analysis

Four sets of p53 target genes reported in two publications were used for the enrichment analysis in the 4FP data set. In the first case, we evaluated the complete set of p53 target genes reported in Supplemental File S6 from Andrysik et al. (2017) (p53 – 1) as well as core p53 target genes defined in this gene set (p53 – 2). In the second case, we evaluated all compiled p53 target genes of Supplementary Table S1 from Fischer (2017) (p53 – 3) as well as a restricted set of human genes from this table which contained p53 bound near the promoter (p53 – 4). Enrichment (overlap between the gene sets is significantly larger than expected at random; odds ratio $> 1$) and significance of enrichment were determined with an exact Fisher's test. Multiple testing correction was performed with the method by Benjamini and Hochberg (1995).

### RNA extraction and RT-qPCR Analysis

Eighty % confluent HeLa Flp-In cells grown on 6-well plates were left untreated or treated with DMSO, 5 μM of 4-NQO, or co-treated with 5 μM of 4-NQO and 250 nM of flavopiridol for 15 min, 30 min, 1 h and 2 h. In experiments with the p38$^{MAPK}$ inhibitor SB203580, HeLa Flp-In cells were pre-treated with 10 μM of the inhibitor for 1 h. HCT116 *TP53*$^{+/+}$, HCT116 *TP53*$^{-/-}$ and siRNA-treated HeLa Flp-In cells were left untreated or treated at eighty % confluency with DMSO or 5 μM of 4-NQO for 30 min, 1 h and 2 h. RNA samples were extracted using TRI Reagent (Sigma), DNase-treated with the Turbo DNA-Free kit (Thermo Fisher Scientific), and reverse transcribed with M-MLV reverse transcriptase (Thermo Fisher Scientific) and random hexamers (Thermo Fisher Scientific) according to the manufacturers' instructions. qPCR reactions were performed with diluted cDNAs, primer pairs that spanned exon-intron junction, and FastStart Universal SYBR Green QPCR Master (Rox) (Sigma) using Stratagene Mx3005 qPCR machine. Primers were from Integrated DNA Technologies and designed using PrimerQuest Tool. Relative levels of transcripts were calculated using the MxPro QPCR Software v4.10. For 7SK, 7SK DNA value was used as a normalizer. For other RNAs, *GAPDH* mRNA values were used as a normalizer. Results from at least three independent experiments are presented as the mean ± SEM. Sequences of the primers used in RT-qPCR assays are listed in Table S5B.

### Quantitative Chromatin Immunoprecipitation Assay

ChIP-qPCR assay was performed as described previously (Ekumi et al., 2015) with the following modifications. HeLa Flp-In cells grown on 15 cm plates were treated at approximately 90% confluency with DMSO or 5 μM of 4-NQO for 2 h. For the total Pol II and Ser2-P Pol II assays, the formaldehyde cross-linked cell pellets were lysed in 800 μL of RIPA buffer (50 mM Tris pH 8.0, 150 mM NaCL, 5 mM EDTA pH 8.0, 0.5% sodium deoxycholate, 1% NP-40, 0.1% SDS) in the presence of EDTA-free Protease Inhibitor Cocktail (Sigma) and SUPERase In RNase Inhibitor (Thermo Fisher Scientific). Lysates were then sonicated during one round of 35 cycles of 30 s ON/30 s OFF at 4°C with the Bioruptor Plus sonication device (Diagenode, B01020001) combined with the Bioruptor Water cooler (Cat. No. BioAcc-cool) & Single Cycle Valve (Cat. No. VB-100-0001) at high power setting (position H) using 1.5 mL TPX microtubes (Diagenode, M-50001). After centrifugation at 13 000 g for 15 min, 2.5% of the cleared chromatin was stored at −80°C for determining DNA input. The rest of the sample was divided in three equal parts, which were supplemented with additional 600 μL of RIPA buffer and incubated overnight at 4°C with 14 μL of antibody-coupled protein G Dynabeads (Thermo Fisher Scientific). Before adding the cleared chromatin, the beads were pre-blocked with bovine serum albumin and salmon sperm DNA overnight at a final concentration of 0.2 μg/μl, pre-incubated in 500 μL of RIPA buffer for 4 h with the antibody and collected by magnetic stand to remove the unbound antibody. We used 2 μg of anti-RNA polymerase II RPB1 (Abcam), 1 μg of anti-RNA polymerase II CTD repeat YSPTSPS (phospho S2) (Abcam), and 3 μg of anti-CDK9 (Cell Signaling Technology) antibodies. Additionally, 2 μg of normal mouse IgG (Santa Cruz Biotechnology) and normal rabbit IgG (Santa Cruz Biotechnology) antibodies were used to determine specificity of the signals. Validation for all antibodies is provided on the manufacturers' websites. For the CDK9 assays, the cells were cross-linked using ChIP Cross-link Gold reagent (Diagenode) and formaldehyde according to manufacturer's instructions and lysed as above. Lysates were sonicated during 30 cycles of 15 s sonication with the Misonix XL-2000 Ultrasonic Liquid Processor using the P-1 Microprobe 3.2 mm tip, power setting 6, in 1.7 mL RNase/DNase-free microcentrifuge tubes (Sigma), which were kept for 1 min on ice between the cycles. Generally, 1/60th of the precipitated ChIP sample was used for each qPCR reaction. For input DNA, 1/100th of the DNA input dissolved in 100 μL of water was used for each qPCR reaction. Samples were amplified using FastStart Universal SYBR Green QPCR Master (Rox) (Sigma), DNA-specific primer pair, and LightCycler 480 II (Roche) machine.

Primers were from Integrated DNA Technologies and designed using PrimerQuest Tool. Values were normalized to their levels in DNA inputs and calculated as a ratio to the values obtained from the DMSO control samples. Ser2-P ChIP values were further normalized to the values of total Pol II. Results from three independent experiments are presented as the mean ± SEM. Sequences of the primers used in ChIP-qPCR assays along with their genomic locations are listed in Table S5C.

### RNA Interference, siRNAs and antisense-oligonucleotide treatment

For RT-qPCR, cytotoxicity, viability, and apoptosis assays, cells grown on 6-well plates were transfected with 50 pmol per well of the indicated siRNA for 48 h. For co-immunoprecipitation assays, cells grown on 10 cm plates were transfected with 400 pmol per plate of the indicated siRNA for 48 h. For ChIP-qPCR assay, cells grown on 15 cm plates were transfected with 450 pmol per plate of the indicated siRNA for 48 h. 7SK was depleted for 48 h using phosphorothioate-modified antisense DNA oligonucleotide (as7SK). 100 pmol per 6-well plate well of the antisense DNA oligonucleotide was used. Transfections were performed using Lipofectamine RNAiMAX reagent (Thermo Fisher Scientific) according to the manufacturer's instructions. Control siRNA was from QIAGEN. siRNA and as7SK oligonucleotide sequences are listed in the Key Resources Table. Efficiency of the knockdowns were evaluated by western blotting or RT-qPCR assays.

### Cytotoxicity Assay

HeLa Flp-In, RPE-1, HFF-1 and HCT116 cells were seeded on 96-well plates 16 h before experiment to ensure 80% confluency. Concentrations of chronic treatments with 4-NQO were 250 nM (HeLa Flp-In), 500 nM (HFF-1), and 1 μM (RPE-1 and HCT116). Where indicated, 250 nM of flavopiridol was used. Flavopiridol concentration for experiments with HCT116 cells was 100 nM. Doses of UV irradiation were 40 J/$m^2$ (HeLa) and 60 J/$m^2$ (RPE-1). Cytotoxicity was evaluated using CellTox Green Cytotoxicity Assay (Promega). CellTox Green Dye was added to the cells together with chemicals or immediately after UV irradiation. Fluorescence was measured at the indicated time points using PerkinElmer Victor X3 Reader. Results from three independent experiments are presented as fluorescence values relative to the untreated control and plotted as the mean ± SEM.

### Viability Assay

HeLa Flp-In cells were seeded on 96-well plates 16 h before experiment to ensure 80% confluency and subjected to the same experimental conditions as in cytotoxicity assays. Cell viability was examined using alamarBlue Cell Viability Assay (Thermo Fisher Scientific). Fresh medium containing the alamarBlue cell viability reagent was added to the cells 2 h prior to the indicated time points. Fluorescence was measured at the indicated time points using PerkinElmer Victor X3 Reader. Results from three independent experiments are presented as fluorescence values relative to the untreated control and plotted as the mean ± SEM.

### Apoptosis Assay

HeLa Flp-In cells were seeded on 96-well plates 16 h before experiment to ensure 80% confluency and subjected to the same experimental conditions as in cytotoxicity assays. Activation of apoptosis was assessed using RealTime-Glo Annexin V Apoptosis and Necrosis Assay (Promega). Luminescence was measured at the indicated time points using PerkinElmer Victor X3 Reader. Results from three independent experiments are presented as luminescence values relative to the untreated control and plotted as the mean ± SEM.

## QUANTIFICATION AND STATISTICAL ANALYSIS

Differential gene expression analysis of 4sU-seq data was performed using edgeR (Robinson et al., 2010). P values obtained from edgeR were corrected by multiple testing using the method by Benjamini and Hochberg (1995) for adjusting the false discovery rate (FDR) and a p value cutoff of 0.01 was applied. Data shown for all qPCR-based experiments and functional assays were collected from at least 3 biological replicates as indicated in individual figure legends and are presented as means ± SEM. Statistical significance and p values were determined by one-tailed Student's t test performed between the indicated paired groups of biological replicates. Where the results were not statistically significant, p values are not indicated.

## DATA AND SOFTWARE AVAILABILITY

### Software

See Key Resources Table.

### Data Resources

The RBM7 iCLIP data have been deposited to ArrayExpress Archive (EMBL-EBI) under the accession code E-MTAB-6475. The 4sU-seq data have been deposited to Gene Expression Omnibus (GEO) repository (NCBI) under the accession code GEO: GSE110272.

**Molecular Cell, Volume *74***

## Supplemental Information

# P-TEFb Activation by RBM7 Shapes a Pro-survival Transcriptional Response to Genotoxic Stress

**Andrii Bugai, Alexandre J.C. Quaresma, Caroline C. Friedel, Tina Lenasi, Robert Düster, Christopher R. Sibley, Koh Fujinaga, Petra Kukanja, Thomas Hennig, Melanie Blasius, Matthias Geyer, Jernej Ule, Lars Dölken, and Matjaž Barborič**

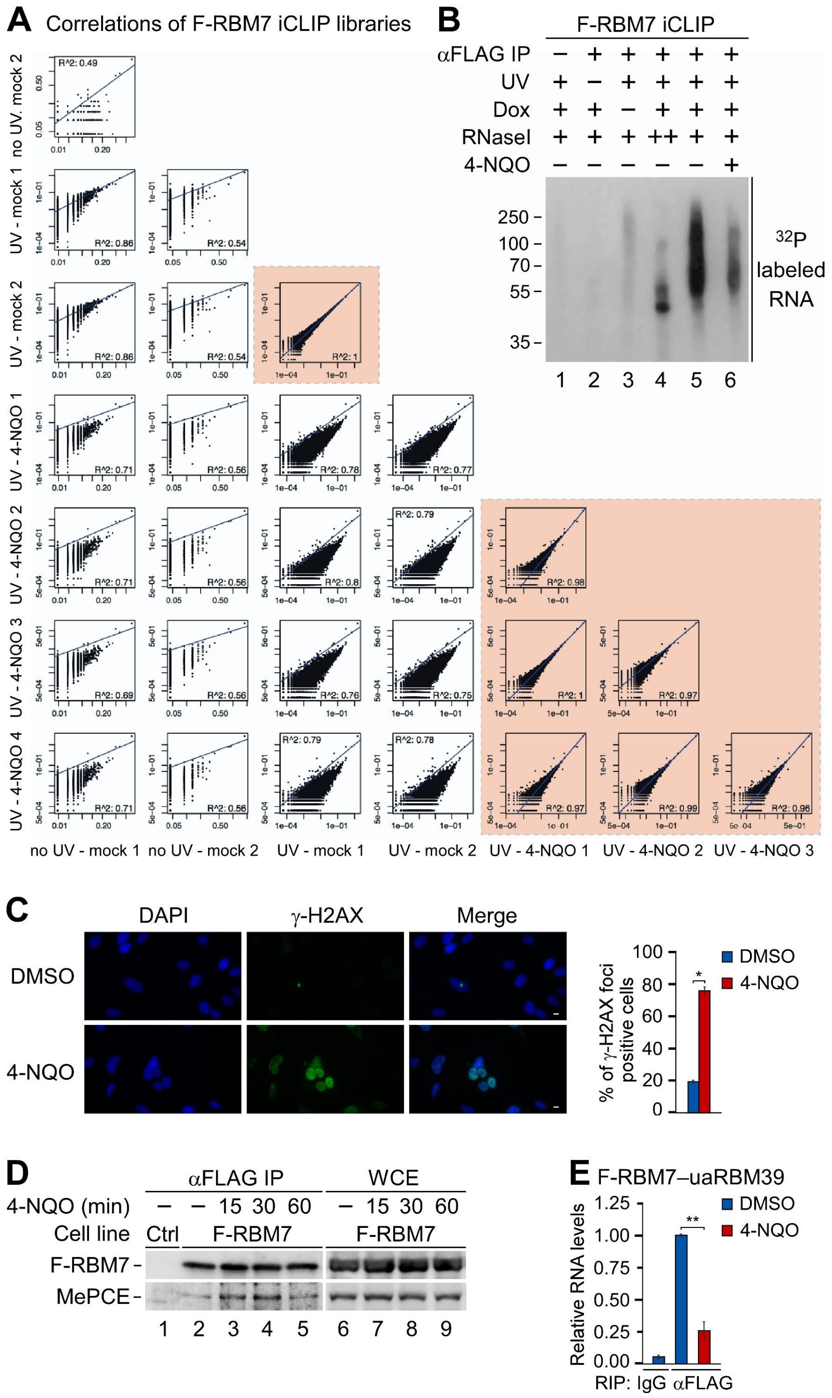


**Figure S1│ F-RBM7 iCLIP.** Related to Figure 1.

(A) Correlations between raw sequencing data of the indicated replicates of F-RBM7 iCLIP libraries.
(B) Autoradiogram of 32P-labelled RNA crosslinked to F-RBM7 in iCLIP. Prominent RNA–protein complexes are seen in the FLAG-M2 immuno-purifications (aFLAG IP) from HEK 293 cells expressing F-RBM7 upon induction by doxycycline (Dox) but not from control cells. Numbers on the left indicate molecular weight in kilodaltons.
(C) (Left) Representative DAPI and anti-g-H2AX staining images of HeLa cells are shown. Cells were treated with DMSO and 4-NQO as indicated. (Right) Quantification of g-H2AX foci-positive HeLa cells. The graph shows the percentage of cells with at least one nuclear g-H2AX foci. Values are plotted as the mean ± s.e.m. (n = 2). *, $P < 0.01$. Size bar = 10 mm.
(D) CoIP of F-RBM7 with MePCE from WCE of HEK 293 cells. Conditions with (in minutes) and without (-) 4-NQO are shown.
(E) RIP-qPCR uaRBM39 in F-RBM7 IP from HEK 293 WCE. Conditions with (red bars) and without (blue bars) 4-NQO (0.5 hr) are shown. Results are presented as the mean ± s.e.m. (n = 3). **, $P < 0.01$.

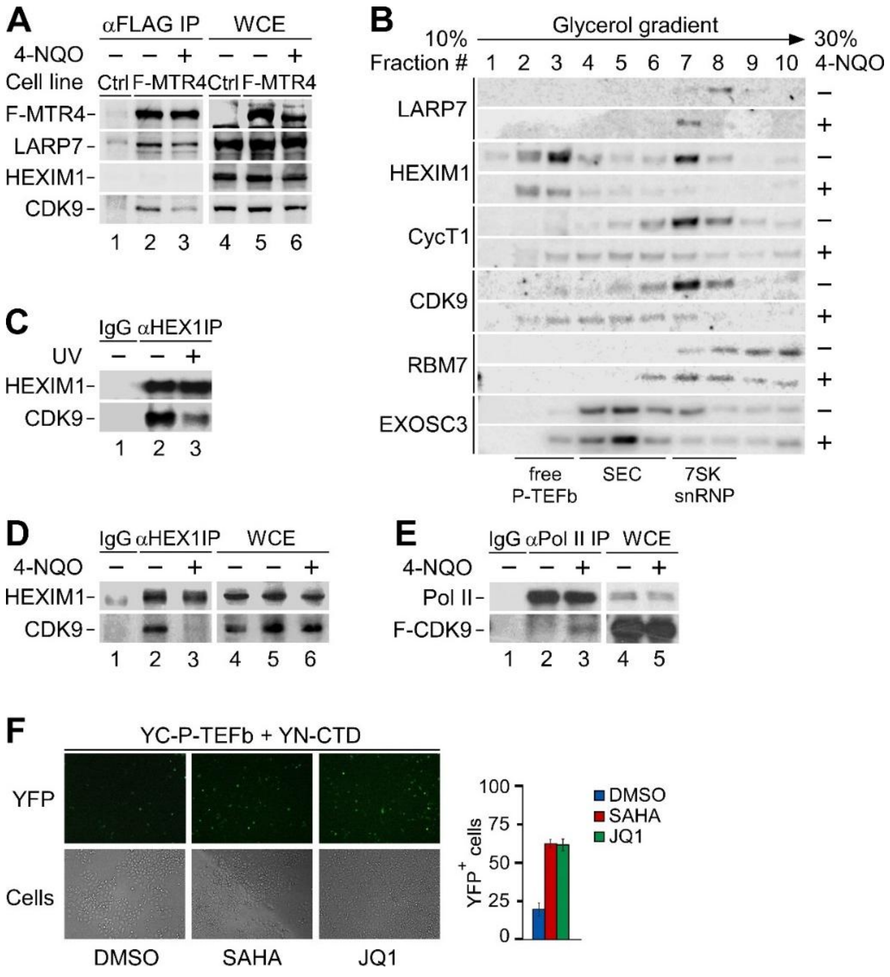


**Figure S2│Genotoxic stress induces the relocation of P-TEFb from 7SK snRNP to Pol II.** Related to Figure 2.

(A) CoIP of F-MTR4 with 7SK snRNP from WCE of HEK 293 cells. Conditions with (+) and without (-) 4-NQO are shown.
(B) Glycerol gradient (10 – 30 %) sedimentation analysis of WCE from HeLa cells. Conditions with (+) and without (-) 4-NQO are shown. The indicated proteins in collected fractions were detected by Western blotting. Fractions that contain P-TEFb in 7SK snRNP, super elongation complexes (SEC), and outside of these complexes are highlighted.
(C,D) CoIP of HEXIM1 with CDK9 from WCE of HeLa (C) and HFF-1 (D) cells. Conditions with (+) and without (-) UV are shown.
(E) CoIP of Pol II with F-CDK9 from WCE of HEK 293 cells. Conditions with (+) and without (-) 4-NQO are shown.
(F) V-PAC assay in HeLa cells expressing YC-P-TEFb and YN-CTD chimera. (Left) Representative YFP fluorescence (YFP) and phase contrast (Cells) images of cells are shown. Cell treatments are shown below the images. (Right) Quantification of YFP-positive cells that were treated as indicated. Values are plotted as the mean ± s.e.m. (n = 2).

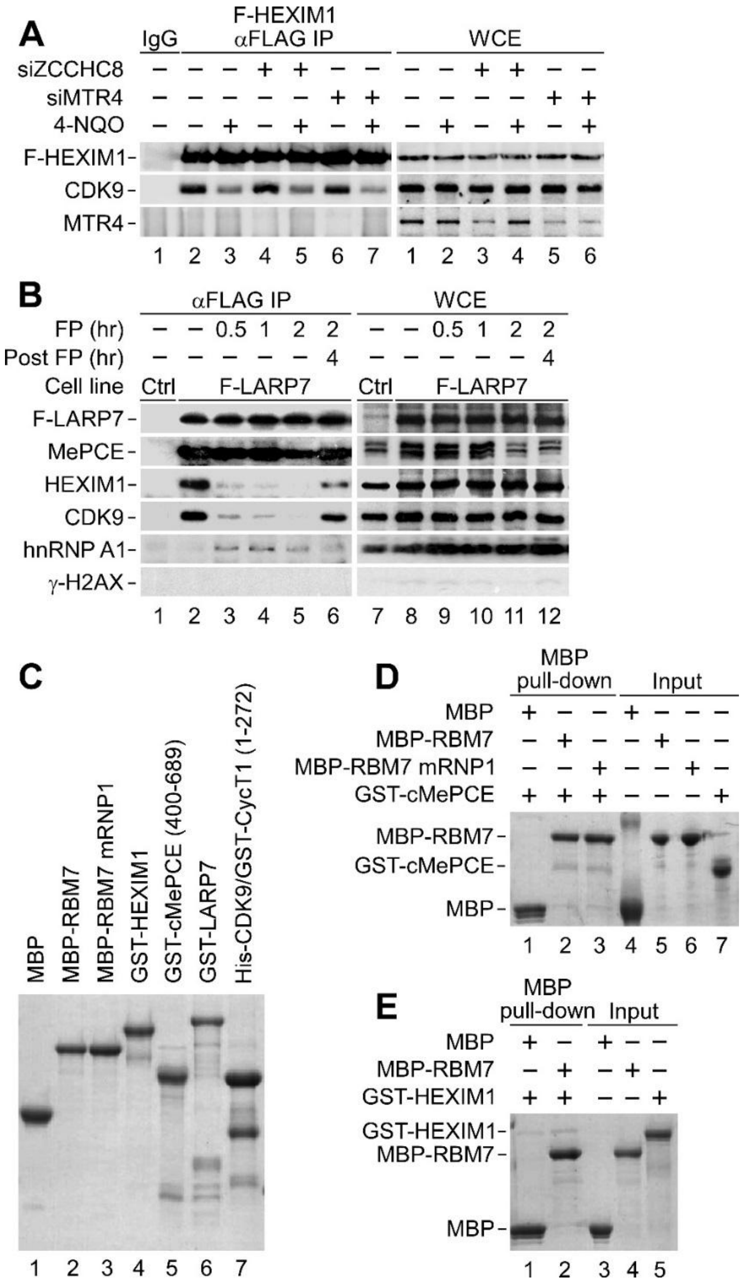


**Figure S3│ RBM7 releases P-TEFb from the core of 7SK snRNP upon genotoxic stress.** Related to Figure 4.

(A) CoIP of F-HEXIM1 with CDK9 from WCE of HEK 293 cells. Conditions with control (-), ZCCHC8 and MTR4 siRNA (+) are shown. The cells were treated (+) or not (-) with 4-NQO.
(B) Co-IP of F-LARP7 with 7SK snRNP and g-H2AX from WCE of HEK 293 cells. Conditions with (in hours) and without (-) FP are shown.
(C) Coomassie-stained gel of recombinant proteins used in *in vitro* MBP pull-down assays.
(D,E) Coomassie-stained gels of *in vitro* MBP pull-down assays of MBP-RBM7 proteins with GST-cMePCE (D) and GST-HEXIM1 (E).

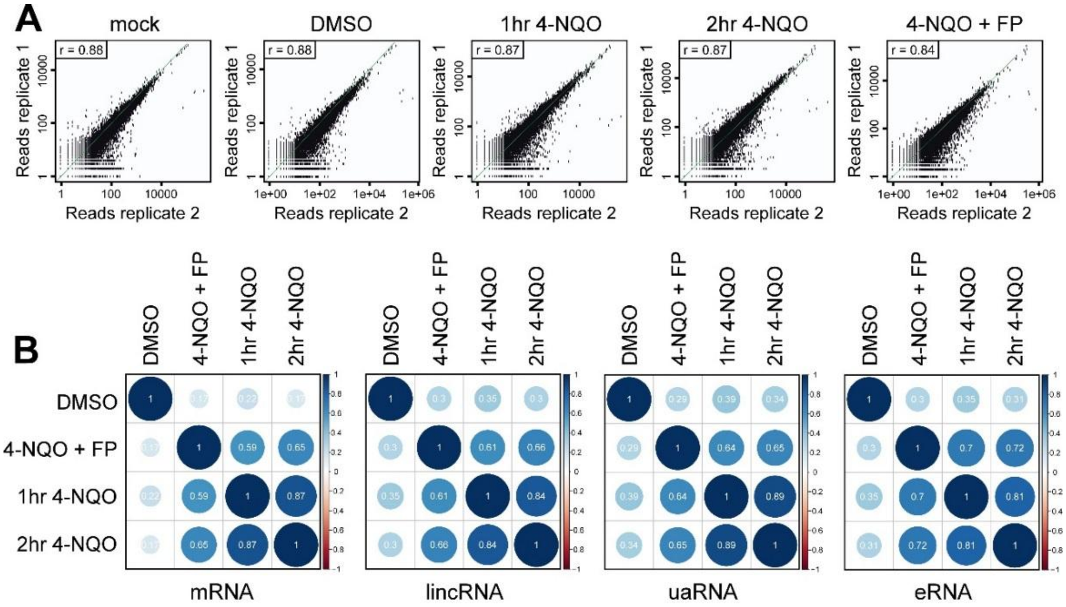


C

| Enriched TF Binding Motif | High Confidence TF | Enrichment Score | Enriched Genes |
|---|---|---|---|
| dbcorrdb__NFYB__ENCSR000EGQ_1__m1 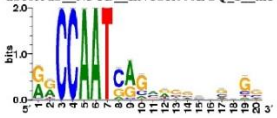 | NF-YB | 4,02 | 109 |
| cisbp__M6174 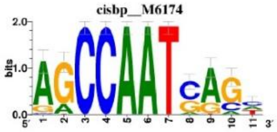 | C/EBPζ | 3,97 | 75 |
| cisbp__M3089 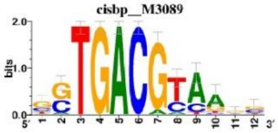 | CREB1 | 3,97 | 90 |
| swissregulon__hs__ATF4.p2 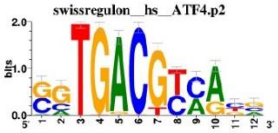 | ATF4 | 3,95 | 120 |
| dbcorrdb__FOS__ENCSR000EYZ_1__m1 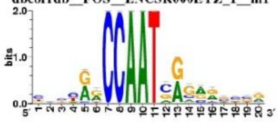 | FOS | 3,63 | 125 |
| cisbp__M0311 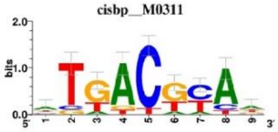 | JUN | 3,60 | 56 |

**Figure S4│ Active P-TEFb is vital for Pol II transcriptional response to genotoxic stress.** Related to Figure 5.

(A) Scatterplots comparing raw read counts for protein-coding and lincRNA genes from 4sU-seq data sets (n = 2) are shown and spearman correlation (*r*) is indicated. HeLa cells were treated as indicated on top of each graph.
(B) Spearman correlation between log2 fold-change values from 4sU-seq data sets (n = 2) are shown. HeLa cells were treated as indicated. Type of RNA transcript is shown below the graphs.
(C) Top transcription factor binding motifs within 5kb window around TSS of the 4FP gene set as revealed by RcisTarget. For each enriched motif, the annotated high confidence TF, normalized enrichment score, and number of genes highly ranked to the motif are shown.

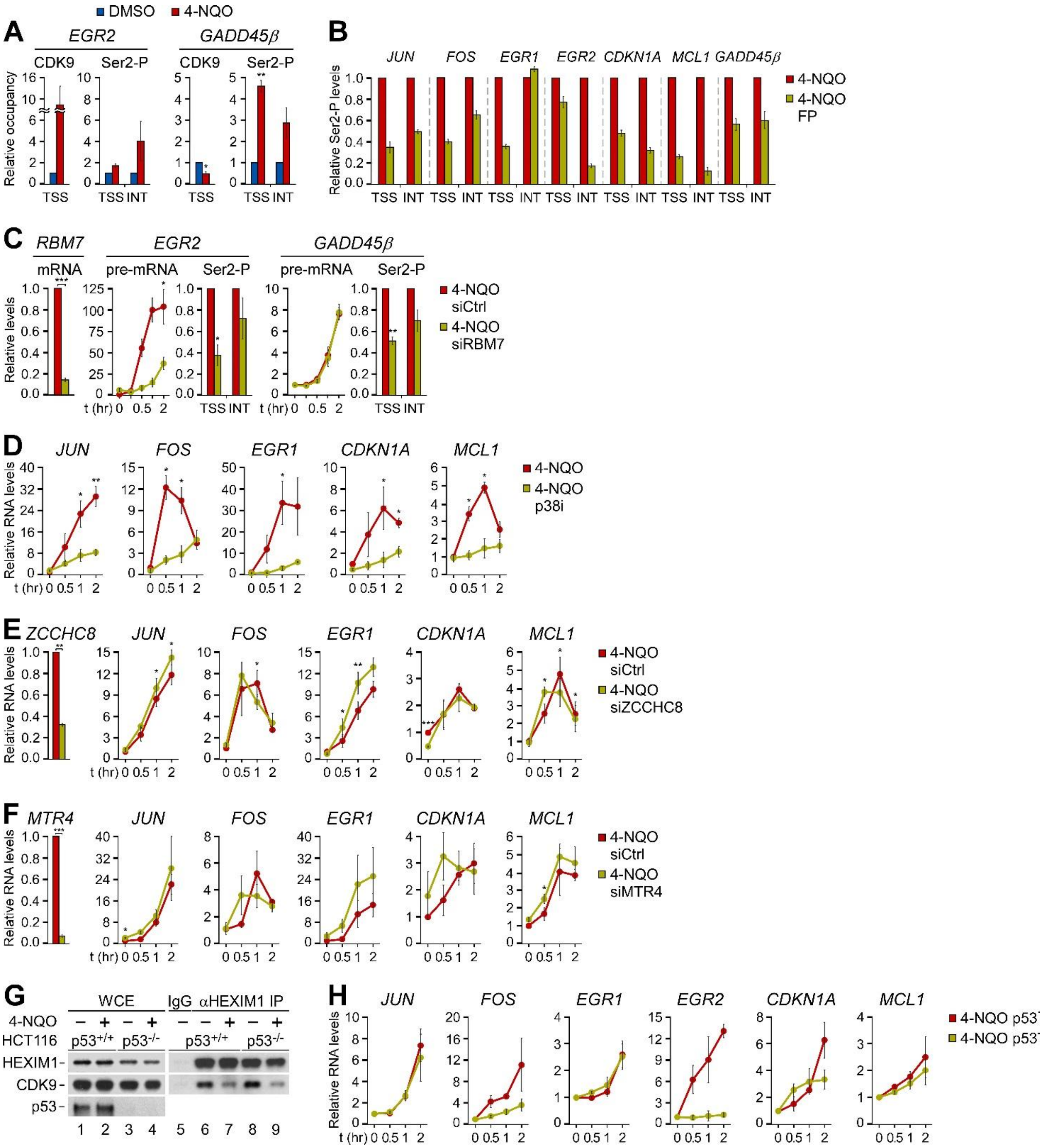


**Figure S5│ RBM7 and 7SK snRNP are critical for the induction of P-TEFb-dependent DDR genes.** Related to Figure 6.

(A) ChIP-qPCR of the occupancy of CDK9 and Ser2-P relative to Pol II at transcription start site (TSS) and in the middle of gene interior (INT) of the indicated DDR genes. Conditions with (red bars) and without (blue bars) 4-NQO are shown. Results were normalized to the DMSO control and are presented as the mean ± s.e.m. (n = 3). *, $P < 0.05$; **, $P < 0.01$, determined by Student's *t* test.
(B) ChIP-qPCR of the levels of Ser2-P relative to Pol II at transcription start site (TSS) and in the middle of gene interior (INT) of the indicated DDR genes. Conditions with 4-NQO (red bars) and with 4-NQO together with FP (yellow bars) are shown. Results were normalized to the 4-NQO values that were set to 1 and are presented as the mean ± s.e.m. Data shown are from one representative experiment (n = 3 technical replicates).
(C) RT-qPCR (left) of unspliced transcripts (pre-mRNA) of the indicated DDR genes and ChIP-qPCR (right) of the levels of Ser2-P relative to Pol II at transcription start site (TSS) and in the middle of gene interior (INT) of the indicated DDR genes

in control (siCtrl; red) and *RBM7* knockdown (siRBM7 #2; yellow) 4-NQO-treated HeLa cells. In RT-qPCR assays, the cells were exposed to 4-NQO for 15 min, 0.5 hr, 1 hr and 2 hr as indicated, and results were normalized to the untreated control and are presented as the mean ± s.e.m. (n = 3). *, P < 0.05; **, P < 0.01; ***, P < 0.001, determined by Student's *t* test. ChIP-qPCR results were normalized to the control values that were set to 1 and are presented as the mean ± s.e.m. (n = 3). *, P < 0.05; **, P < 0.01, determined by Student's *t* test. Efficacy of the *RBM7* knockdown is shown on the left. *** P < 0.001, determined by Student's *t* test.

(D) RT-qPCR of unspliced transcripts of the indicated DDR genes in 4-NQO-treated HeLa cells which were pre-treated or not with the p38 inhibitor (p38i) SB203580 for 1 hr. The cells were exposed to 4-NQO for 0.5 hr, 1 hr and 2 hr as indicated, and results were normalized to the untreated control and are presented as the mean ± s.e.m. (n = 3). *, P < 0.05; **, P < 0.01, determined by Student's *t* test.

(E,F) RT-qPCR of unspliced transcripts of the indicated DDR genes in control (siCtrl; red) and *ZCCHC8* (E; siZCCHC8, yellow) or *MTR4* (F; siMTR4, yellow) knockdown 4-NQO-treated HeLa cells. The cells were exposed to 4-NQO for 0.5 hr, 1 hr and 2 hr as indicated, and results were normalized to the untreated control and are presented as the mean ± s.e.m. (n = 3). *, P < 0.05; **, P < 0.01; ***, P < 0.001, determined by Student's *t* test. Efficacy of the *ZCCHC8* and *MTR4* knockdowns are shown on the left. **, P < 0.01; ***, P < 0.001, determined by Student's *t* test.

(G) CoIP of HEXIM1 with CDK9 from WCE of HCT116 $p53^{+/+}$ and HCT116 $p53^{-/-}$ cell lines. Conditions with (+) and without (-) UV are shown.

(H) RT-qPCR of unspliced transcripts of the indicated DDR genes in 4-NQO-treated HCT116 $p53^{+/+}$ (red) and HCT116 $p53^{-/-}$ (yellow) cell lines. The cells were exposed to 4-NQO for 0.5 hr, 1 hr and 2 hr as indicated, and results were normalized to the respective untreated control and are presented as the mean ± s.e.m. (n = 3).

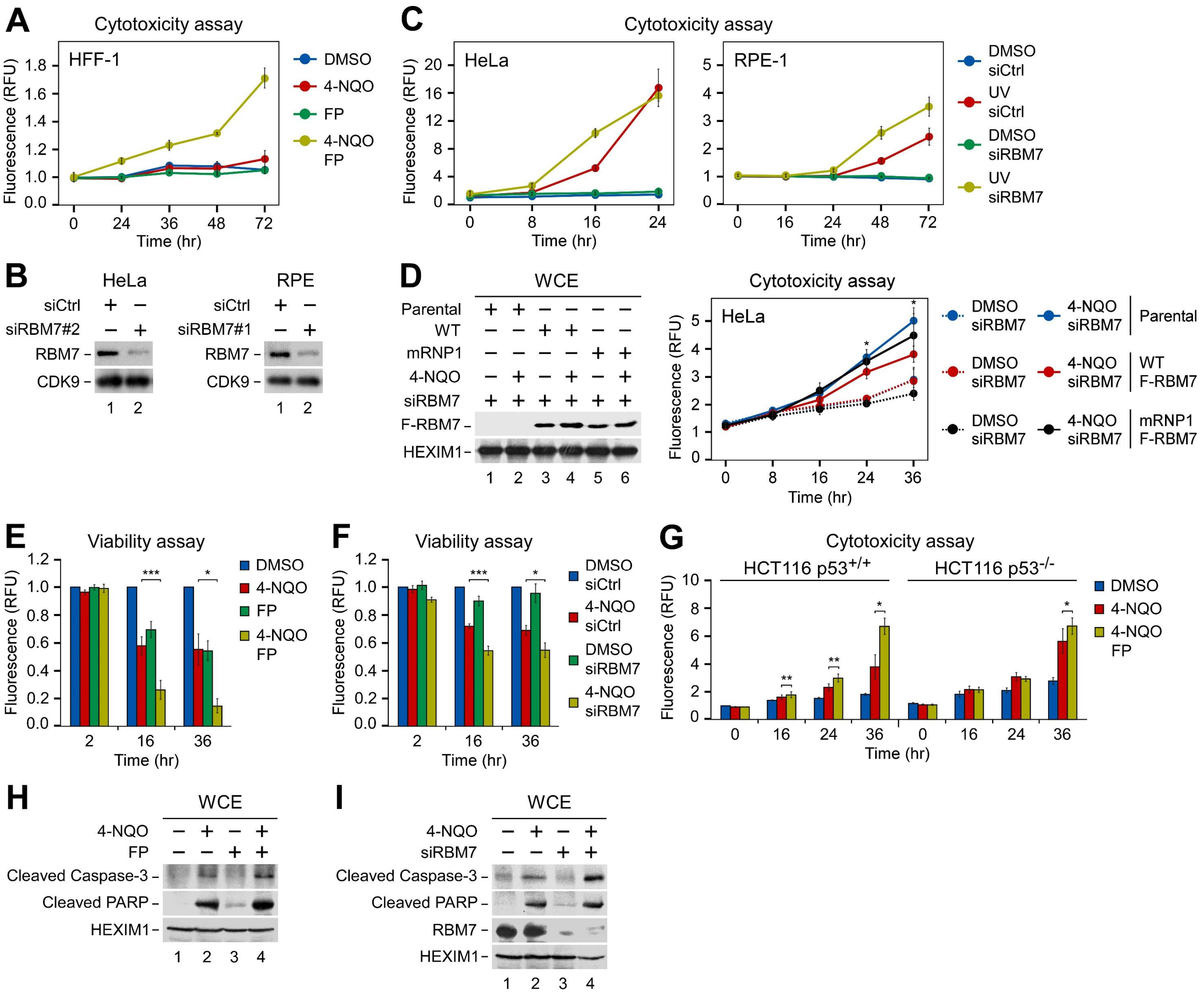


**Figure S6│ P-TEFb and RBM7 promote cell viability upon genotoxic stress.** Related to Figure 7.

(A,C) Hypersensitivity of HFF-1, HeLa and RPE-1 cells to genotoxic stress upon FP treatment (A) and RBM7 depletion (C). The cells were treated as indicated by the legends and examined at the time points indicated below the graphs. Two independent siRNAs (siRBM7 #2, HeLa cells; siRBM7 #1, RPE-1 cells) were used to deplete RBM7. Cytotoxicity results are presented as fluorescence values relative to the untreated control and plotted as the mean ± s.e.m. (n = 2).

(B) Western blotting analysis of RBM7 depletion in HeLa and RPE-1 cells using RNAi.

(D) Expression of F-RBM7 increases survival of 4-NQO-treated HeLa cells with depleted levels of endogenous RBM7. The parental, WT and mRNP1 F-RBM7-expressing HeLa cells were treated as indicated by the legends and examined at the time points indicated below the graphs. siRBM7 #2 was used to deplete RBM7. Cytotoxicity results are presented as fluorescence values relative to the untreated control and plotted as the mean ± s.e.m. (n = 3). *, $P < 0.05$, determined by Student's *t* test using 4-NQO siRBM7 data sets from parental and F-RBM7 WT HeLa cells. Levels of the F-RBM7 proteins in *RBM7* knockdown cells are shown on the left.

(E,F) FP treatment (E) and RBM7 depletion (F) decrease viability of 4-NQO-treated HeLa cells. The cells were treated as indicated by the legends and examined at the time points indicated below the graphs. siRBM7 #2 was used to deplete RBM7. Viability results are presented as fluorescence values relative to the untreated control and plotted as the mean ± s.e.m. (n = 3). *, $P < 0.05$; ***, $P < 0.001$, determined by Student's *t* test.

(G) Hypersensitivity of HCT116 $p53^{+/+}$ cells to genotoxic stress upon FP treatment. HCT116 cell lines were treated as indicated by the legend and examined at the time points indicated below the graphs. Cytotoxicity results are presented as fluorescence values relative to the untreated control in HCT116 $p53^{+/+}$ cells and plotted as the mean ± s.e.m. (n = 3) *, $P < 0.05$; **, $P < 0.01$, determined by Student's *t* test.

(H,I) Western blotting analysis of Caspase-3 and PARP cleavage using the cleaved products-specific antibodies. HeLa cells were treated with DMSO, 4-NQO, and FP for eight hours as indicated. siRBM7 #2 was used to deplete RBM7.

**Table S4. Specificity of CDK9, Pol II and Ser2-P ChIP-qPCR assays.** Related to Figure 6.

Enrichments of CDK9 ChIP values over IgG

| | *JUN* | *FOS* | *EGR1* | *EGR2* | *CDKN1A* | *MCL1* | *GADD45β* |
|---|---|---|---|---|---|---|---|
| **Ab - Treatment** | **TSS** | **TSS** | **TSS** | **TSS** | **TSS** | **TSS** | **TSS** |
| CDK9 - DMSO | 10,4 | 10,8 | 3,8 | 1,5 | 24,7 | 38,4 | 21,9 |
| CDK9 - 4-NQO | 31,2 | 16,0 | 267,3 | 6,6 | 3,1 | 60,9 | 18,0 |

Enrichments of CDK9 ChIP values over *FOS* intergenic region

| | *JUN* | *FOS* | *EGR1* | *EGR2* | *CDKN1A* | *MCL1* | *GADD45β* |
|---|---|---|---|---|---|---|---|
| **Ab - Treatment** | **TSS** | **TSS** | **TSS** | **TSS** | **TSS** | **TSS** | **TSS** |
| CDK9 - DMSO | 2,6 | 7,0 | 1,3 | 0,5 | 1,4 | 4,4 | 11,2 |
| CDK9 - 4-NQO | 7,5 | 17,1 | 12,3 | 2,2 | 2,0 | 8,8 | 5,5 |

Enrichments of Pol II and Ser2-P ChIP values over IgG

| | *JUN* | | *FOS* | | *EGR1* | | *EGR2* | | *CDKN1A* | | *MCL1* | | *GADD45β* | |
|---|---|---|---|---|---|---|---|---|---|---|---|---|---|---|
| **Ab - Treatment** | **TSS** | **INT** | **TSS** | **INT** | **TSS** | **INT** | **TSS** | **INT** | **TSS** | **INT** | **TSS** | **INT** | **TSS** | **INT** |
| Pol II - DMSO | 13,1 | 4,0 | 17,7 | 1,7 | 5,4 | 2,5 | 3,2 | 1,1 | 6,4 | 3,1 | 28,1 | 14,4 | 37,7 | 19,2 |
| Pol II - 4-NQO | 60,8 | 124,1 | 38,6 | 15,0 | 150,4 | 95,3 | 6,9 | 7,8 | 19,6 | 40,0 | 63,1 | 18,1 | 108,0 | 81,3 |
| Ser2-P - DMSO | 7,8 | 7,7 | 11,8 | 10,9 | 5,7 | 3,5 | 2,2 | 1,3 | 3,0 | 22,2 | 26,4 | 112,7 | 24,5 | 41,6 |
| Ser2-P - 4-NQO | 210,5 | 661,7 | 93,0 | 99,5 | 719,2 | 555,0 | 12,9 | 37,0 | 130,9 | 286,8 | 222,8 | 81,9 | 234,9 | 429,1 |

Enrichments of Pol II and Ser2-P ChIP values over *FOS* intergenic region

| | *JUN* | | *FOS* | | *EGR1* | | *EGR2* | | *CDKN1A* | | *MCL1* | | *GADD45β* | |
|---|---|---|---|---|---|---|---|---|---|---|---|---|---|---|
| **Ab - Treatment** | **TSS** | **INT** | **TSS** | **INT** | **TSS** | **INT** | **TSS** | **INT** | **TSS** | **INT** | **TSS** | **INT** | **TSS** | **INT** |
| Pol II - DMSO | 20,8 | 4,1 | 31,3 | 1,7 | 3,4 | 1,8 | 0,9 | 1,0 | 8,1 | 4,1 | 31,6 | 3,2 | 119,0 | 17,5 |
| Pol II - 4-NQO | 24,0 | 19,8 | 49,4 | 9,0 | 19,4 | 14,3 | 1,0 | 3,9 | 1,5 | 5,7 | 29,7 | 10,6 | 43,3 | 24,8 |
| Ser2-P - DMSO | 12,3 | 7,8 | 20,9 | 10,8 | 3,6 | 2,6 | 0,6 | 1,3 | 3,8 | 29,2 | 29,7 | 25,4 | 77,3 | 37,9 |
| Ser2-P - 4-NQO | 83,3 | 105,5 | 119,1 | 59,5 | 92,8 | 83,4 | 1,9 | 18,7 | 9,9 | 40,7 | 104,9 | 47,9 | 94,1 | 131,1 |

Enrichments of the CDK9, Pol II and Ser2-P chromatin occupancies over the normal IgG and *FOS* intergenic site controls are shown. Conditions with (red lines) and without (blue lines) 4-NQO are shown. Data shown are derived from mean % of input values corresponding to the data presented in Figure 6A, and are presented as fold-enrichments over the controls which were set to 1. (n = 3).
